# Aerial ULV control of *Aedes aegypti* with naled (Dibrom) inside simulated rural village and urban cryptic habitats

**DOI:** 10.1371/journal.pone.0191555

**Published:** 2018-01-19

**Authors:** Seth C. Britch, Kenneth J. Linthicum, Robert L. Aldridge, Mark S. Breidenbaugh, Mark D. Latham, Peter H. Connelly, Mattie J. E. Rush, Jennifer L. Remmers, Jerry D. Kerce, Charles A. Silcox, US Navy Entomology Center of Excellence Team

**Affiliations:** 1 United States Department of Agriculture, Agricultural Research Service, Center for Medical, Agricultural, & Veterinary Entomology, Gainesville, Florida, United States of America; 2 910 Airlift Wing, 757 Airlift Squadron, Aerial Spray Branch, Youngstown Air Reserve Station, Vienna, Ohio, United States of America; 3 Manatee County Mosquito Control District, West Palmetto, Florida, United States of America; 4 AMVAC Environmental Products, Newport Beach, California, United States of America; 5 Camp Blanding Joint Training Center, Starke, Florida, United States of America; 6 United States Navy Entomology Center of Excellence, Naval Air Station, Jacksonville, Florida, United States of America; New Mexico State University, UNITED STATES

## Abstract

We conducted aerial fixed wing ultra low volume (ULV) spray trials with naled to investigate penetration of exposed and simulated cryptic habitat within opened buildings, partially sealed buildings, and outdoor locations targeting sentinel adult *Aedes aegypti* mosquitoes in north central Florida. Mortality was observed in open and closed buildings and outdoors, even in mosquitoes placed in cryptic habitats. Observations on the impact of building type, mosquito exposure method such as placement in cryptic habitat, and spray nozzle size on mosquito mortality are described and analyzed.

## Introduction

Recent incursion and local transmission of Zika virus in the United States [1] has led to public health authorities conducting vector control activities against *Aedes aegypti* mosquitoes including aerial application of adulticides, namely, Dibrom® (naled) as part of an integrated vector management (IVM) program [2]. Perhaps the most significant obstacle to control of adult *Ae*. *aegypti* with aerially applied pesticides is reaching mosquitoes sequestered in the protected locations this species favors. This shortfall of aerial control, and for that matter truck and portable adulticide applications against *Ae*. *aegypti*, has been extensively documented in reviews [3,4], operational reports and summaries [5], and controlled experimental trials with a variety of adulticides, conditions, and application equipment [6,7].

A survey of primary and gray literature (S1 File) shows that the great majority of both operational and experimental ground and aerial ultra low volume (ULV) and thermal fog applications against *Ae*. *aegypti* has been conducted with three organophosphate pesticides: malathion (37 publications), fenitrothion (11 publications), and naled (7 publications). Little has been published regarding the ability of aerial ULV applications of naled to affect adult *Ae*. *aegypti* indoors, yet it is one of the most commonly used mosquito adulticides sprayed from the air by the US Air Force and by mosquito control districts in the US [8] (MSB, PHC pers. obs.). Coordination of public health pesticide use will benefit from a greater understanding of the capability of naled to reduce indoor or outdoor sequestered populations of *Ae*. *aegypti*, given the rapid development of Zika virus transmission seen in 2016. Florida, in the US, has experienced local transmission of dengue [9] and has been the first in the US to experience local transmission of both chikungunya and Zika viruses [1,10] in recent years, making an analysis of naled particularly applicable to the Florida programs.

Of the 7 publications identified for naled (S1 File), 6 demonstrate that various fixed-wing, helicopter, truck, and portable aerosol applications may be highly effective against a variety of wild and colony *Ae*. *aegypti* populations in outdoor open areas in a range of ecological conditions. Only a single publication [11] presents data–from an aerial ULV application–showing that naled may effectively reach indoor locations. This publication describes experimental investigations of naled conducted by the US Centers for Disease Control and Prevention (US CDC) against sentinel adult *Ae*. *aegypti* mosquitoes positioned indoors in protected locations during operational aerial ULV applications in response to dengue outbreaks in 1987 and 1988 in San Juan, Puerto Rico. The US CDC 1987 application [12] described in [11] was conducted at the maximum label rate of 1 oz/A with a C-130A fixed wing aircraft modified with a spray system and wing tip spray booms, which resulted in 35–100% (mean 74%) uncorrected mortality in indoor sentinel cages across 5 trials. On the other hand, the US CDC 1988 application described in [11] was conducted at the maximum label rate but with a US Navy PAU-9 spray system [13,14] mounted on a UH-1 helicopter, and results indicated that the indoor sentinels were unaffected by the spray. Unfortunately, the most recent of the 8 naled documents [2] describes a similar fixed wing operational scenario as [11] conducted in Miami, Florida, but with no experimentally placed sentinel mosquitoes to investigate penetration of protected locations such as residential structures.

If we expand our consideration of naled to include studies investigating efficacy of its principal insecticidally active breakdown product DDVP (dichlorvos) [15] against adult *Ae*. *aegypti*, two more publications come to light [16,17]. Both studies include an indoor efficacy component; however the later study describes only efficacy of dichlorvos applied indoors with a portable thermal fogger [17]. On the other hand, the earlier study investigated efficacy of an aerial fixed wing ULV application of dichlorvos against sentinel *Ae*. *aegypti* placed in several indoor locations, and concluded that the application was effective with 70–84% uncorrected mortality at 4 hr post-spray depending on the level of exposure of the room to the outdoors [16].

Considering the importance of naled to aerial ULV mosquito control in Florida and elsewhere and the highly present need to control *Ae*. *aegypti* in protected locations, we designed experimental trials with naled enhanced with advances in aerial ULV application systems that could improve on the encouraging historical results recorded in [11] and [16]. Aerial ULV systems now benefit from advances in technology not available at the time of the prior studies. Among these are: 1) more efficient applications capable of delivering an aerosol cloud with a smaller volume median diameter (VMD; i.e., 50% of the spray volume is made up of droplets of that diameter or less); 2) GPS-based tracking and spray guidance software such as Wingman GX (ADAPCO, Sanford, FL) [18], which integrates real time weather into guidance of the aircraft, and 3) AgDISP [19,20] that calculates the offset necessary to ensure that the targeted area is reached by the aerosol cloud and may place spray swaths with greater precision. The C-130H modular aerial spray system (MASS) has been improved by moving the spray booms from the wing tips to the fuselage [21] which may better energize spray clouds with natural vortices produced in flight to drive pesticide droplets to the target zone. We conducted trials in Florida with a combination of several of these new technologies in simulated urban and rural environments designed for military training which provided an array of options for positioning sentinel mosquitoes and droplet capture apparatus. Our objective was to determine the extent that aerial applications of naled with a US Air Force C-130H with a standard operational configuration can reach adult *Ae*. *aegypti* sequestered in protected outdoor locations or protected locations within buildings in a temperate/sub-tropical environment.

## Methods and materials

### Location

**Fig 1:**
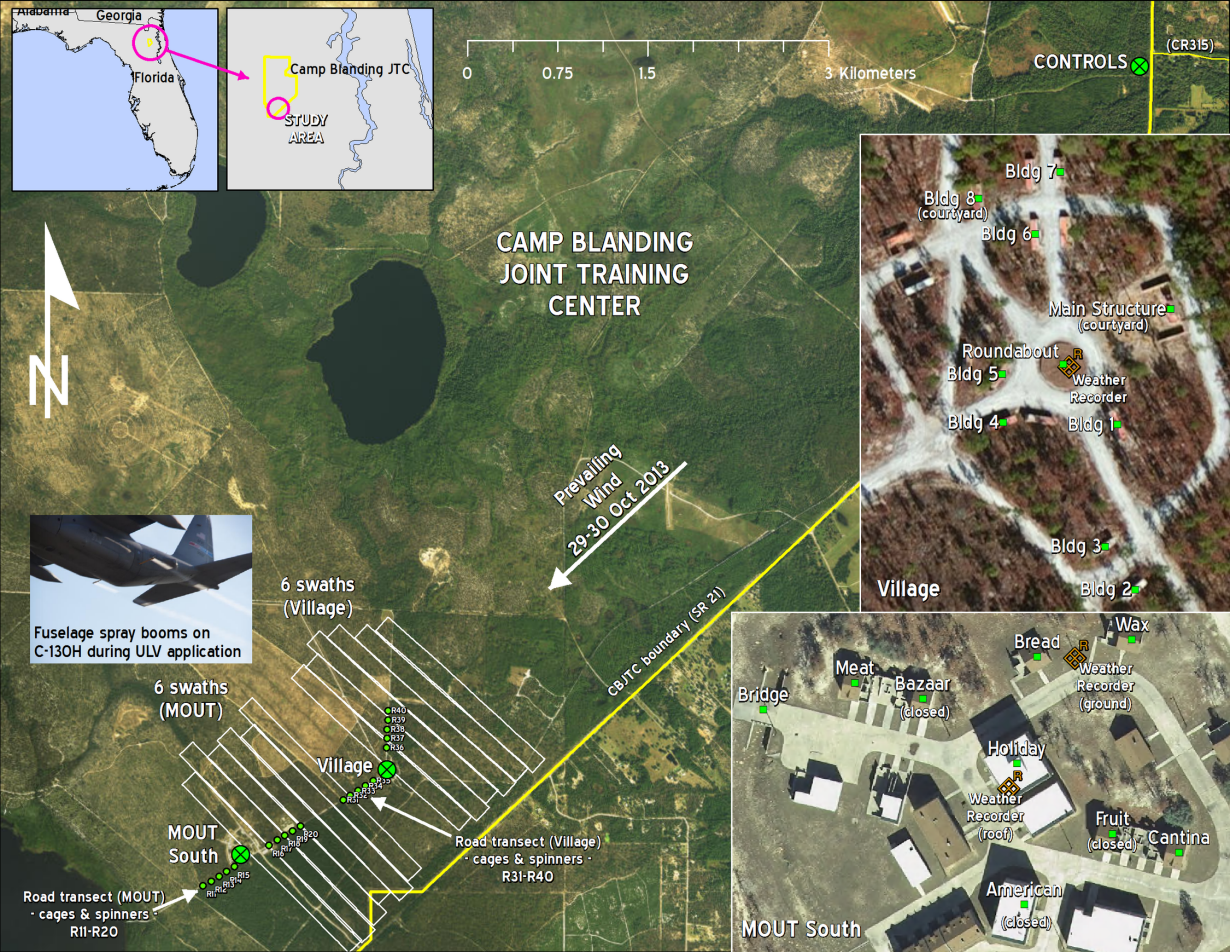
Overview image of the study area. Image shows the position of Controls near the intersection of State Road 21 and County Road 315 (upper right) and the MOUT South, Village, and two road Transect spray areas with positioning of weather recorders. Each Transect spray area consists of 10 sentinel/spinner positions, with 5 positions placed on each side of either MOUT South (R11–R20) or Village (R31–R40) spray areas. Inset Florida state maps at upper left show location of the Camp Blanding Joint Training Center (yellow boundary) and indicate position of study area. The overview also shows the approximate locations of the two sets of 6 upwind swaths and the prevailing wind direction for 29–30 October 2013. Inset images on the mid- and lower right side show detailed views of MOUT South and Village with building names and indications to show which ones were closed up or had courtyard walls present. Inset photo at mid-left shows spray trails from C-130H fuselage booms during one of the applications. Additional detailed maps are in Figure Sets A–J in S1 Fig. Maps were created using ArcGIS software by Esri under license as described in S2 File.

**Fig 2:**
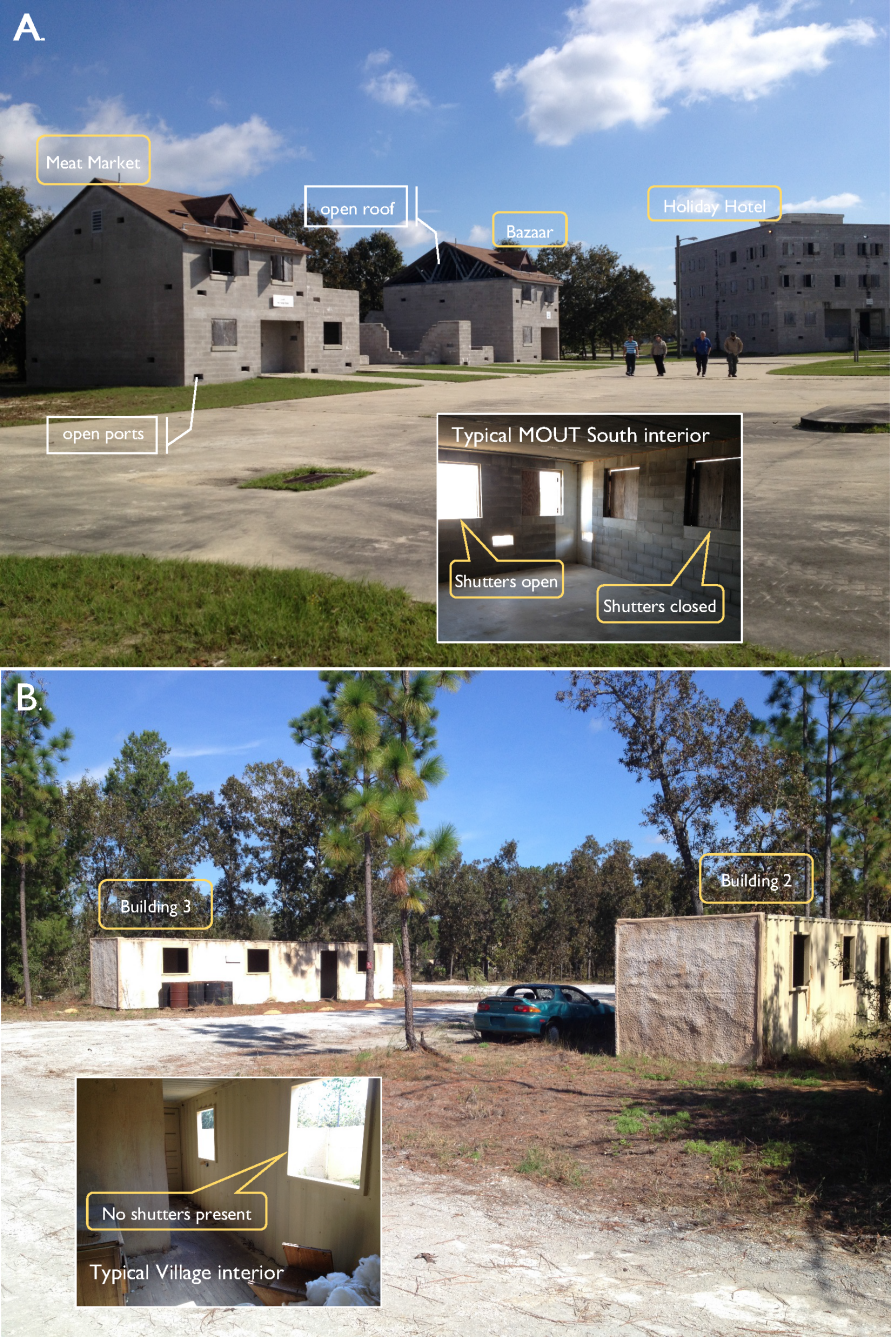
Representative views of MOUT South and Village structures, building interiors, and surrounding habitat. (A) Meat Market, Bazaar, and Holiday Hotel buildings at MOUT South. Inset shows typical interior, with shutters opened and closed. (B) Buildings 2 and 3 at the Village. Note lack of doors or windows, and inset shows typical interior with window openings lacking shutters.

Spray trials were conducted on 29–30 October 2013 in urban warfare training areas at Camp Blanding Joint Training Center (CBJTC) in Starke, Florida. The CBJTC is the primary training base for the Florida Army National Guard consisting of approximately 73,000 acres located 50 miles southwest of Jacksonville. The urban warfare training areas available for field trials at CBJTC were the MOUT (Military Operations in Urban Terrain) South complex, approximately 40 acres centered on 29.853318 N, 81.974948 W, and the Village site, approximately 20 acres centered on 29.859241 N, 81.962419 W (Fig 1). The MOUT site is a close quarter combat training area consisting of several multi-story concrete buildings typical of urban areas worldwide. The Village site is a similar training area but constructed to resemble a small rural village. Each of these sites provided diverse simulated residential and commercial building structures suitable for indoor *Ae*. *aegypti* control studies. The structures presented options for varied exposure to the outside environment, and therefore varied levels of challenge to ULV spray cloud droplet penetration to sentinel mosquitoes and droplet capture apparatus. The structures at the MOUT site could be left open or made partially sealed by using built-in shutters and doors; however, none of them could be completely sealed due to lack of window glass, a variety of engineered openings (cinder block sized, approx. 16-in x 8-in) in the walls, and larger openings of variable size in some of the roofs (Fig 2). Doors, window glass, or shutters were not present in the openings in the Village site structures; doorways and windows were not practical to seal and the structures were left open and unmodified during spray trials (Fig 2). Overall, these contrasting sites could be regarded as covering much of the relative structural living conditions seen in developing communities and those with mostly modernized structures but without air conditioning, resulting in the need to have windows open.

Roadways connecting the two treatment sites were also used for placement of sentinel mosquitoes and droplet capture materials along transects perpendicular to the spray flights to survey for overall efficacy and to confirm the treatment zone (Fig 1). We selected an additional site (29.911570 N, 81.897720 W) approximately 4.7 miles upwind of the farthest spray swath for placement of untreated control materials (Fig 1). Separate teams of personnel linked by radio or mobile phones were positioned at each of the two treatment sites, the road transect sites, and the control site to streamline handling of sentinel mosquitoes and droplet capture apparatus, and to prevent cross-contamination.

### Aerial spray

The USAF C-130H fixed wing platform equipped with Wingman GX differential GPS tracking and AgDISP spray drift modeling guidance was provided by the Aerial Spray Branch of the 910 Airlift Wing, 757 Airlift Squadron from the Youngstown Air Reserve Station, Vienna, OH. The ULV equipment on the C-130 consisted of a 2-Module SP2G flat fan modular aerial spray system (MASS), with two stainless steel ULV fuselage booms (Fig 1 inset). Different nozzle configurations and application rates were used on each day: on 29 October, TeeJet 8001 nozzles (Spraying Systems Company, Wheaton, IL; 12 on each boom and oriented straight down) sprayed 0.84 fl oz/A; on 30 October, 8 flat fan TeeJet 8003 nozzles (4 on each boom and oriented straight down) sprayed 1.00 fl oz/A.

On both days we applied naled in the form of neat Dibrom Concentrate (87.4% naled; AMVAC Chemical Corporation, Los Angeles, CA) adulticide at the targeted maximum label rate of 1.0 fl oz/A over both the MOUT and Village test areas. Each target area received 1 of the 6 upwind 500 ft swaths on each day from the normal Air Force operational altitude of approximately 150 ft above ground level (AGL), and perpendicular to the direction of the prevailing wind at release height (Fig 1). Although theoretically only 1 of the 6 swaths would be expected to impact each treatment area, since each area was ≤500 ft across, the 5 additional upwind swaths at each area were applied to ensure naled reached target treatment areas regardless of meteorological shifts and to mimic operational standard for mosquito control. The 150 ft AGL release height is also the minimum safe application height and higher application heights were not used in order to maximize presence of aircraft vortex energy to force naled into the treatment area. We used the AgDISP technology to guide estimation of appropriate offsets, taking into account meteorology (i.e., wind speed, wind direction, temperature, and humidity) at the release height and at the target site to ensure appropriate flight paths for optimal delivery of insecticide. Meteorology was measured and recorded in real time with aircraft instrumentation at release height, on the roof of the Holiday Hotel (Fig 1) using Airmar LB150 Ultrasonic WeatherStation Instrument (AIRMAR Technology Corp.; Milford, NH), and on the ground at the MOUT South and Village sites using Kestrel 4500NV Weather Trackers with wind vane kits (Nielsen-Kellerman; Boothwyn, PA) (Fig 1). Camp Blanding range boundaries and other flight path restrictions were supervised by CBJTC Range Control and coordinated with flight crews.

### Sentinel mosquitoes

No native/wild population of *Ae*. *aegypti* was present at the two study sites, so we evaluated the efficacy of the aerial application by placing cages of sentinel adult *Ae*. *aegypti* mosquitoes in key open and protected locations in the 4 field sites (Fig 1): the MOUT South and Village sites, along road Transect sites perpendicular to the spray lines, and at the upwind Control site. Sentinel *Ae*. *aegypti* adult mosquitoes were drawn from the susceptible Orlando strain reared since 1952 under controlled insectary conditions of 27±2°C, 30–70% relative humidity, and a photoperiod of 12:12 (light:dark) hr at the USDA Agricultural Research Service Center for Medical, Agricultural, and Veterinary Entomology (CMAVE) in Gainesville, FL. Pupae from the CMAVE colony were reared to 2–3 day old adults in 14.5-in x 14.5-in x 18-in screened population cages with 10% sucrose solution-soaked cotton balls as a nutrient source. Each morning before aerial sprays were conducted we used these colony specimens to prepare 130 adult *Ae*. *aegypti* sentinel mosquito cages in the CMAVE laboratory. We removed approximately 100 adult *Ae*. *aegypti* at a time from the population cages using a mechanical aspirator and anaesthetized them under CO_2_ for 4 min. The anaesthetized specimens were spread onto a sheet of white paper and we used the Wynn-Gun aspirator [22] to transfer 20 female *Ae*. *aegypti* into each sentinel cage. This process was repeated until all 130 cages were prepared. We used field sentinel mosquito cages identical to those described in [23], and supplied each cage with a cotton ball soaked in 5% sucrose solution to reduce stress.

We divided the sentinel cages into four insulated 48 qt plastic picnic coolers lined with moistened towels to maintain a low-stress environment for the mosquitoes and to facilitate storage and transport of the cages to the 4 field sites. To prevent cross-contamination we supplied a second similarly configured picnic cooler at each site to store sentinel cages post-spray, and transport them back to the CMAVE laboratory for observation.

**Fig 3:**
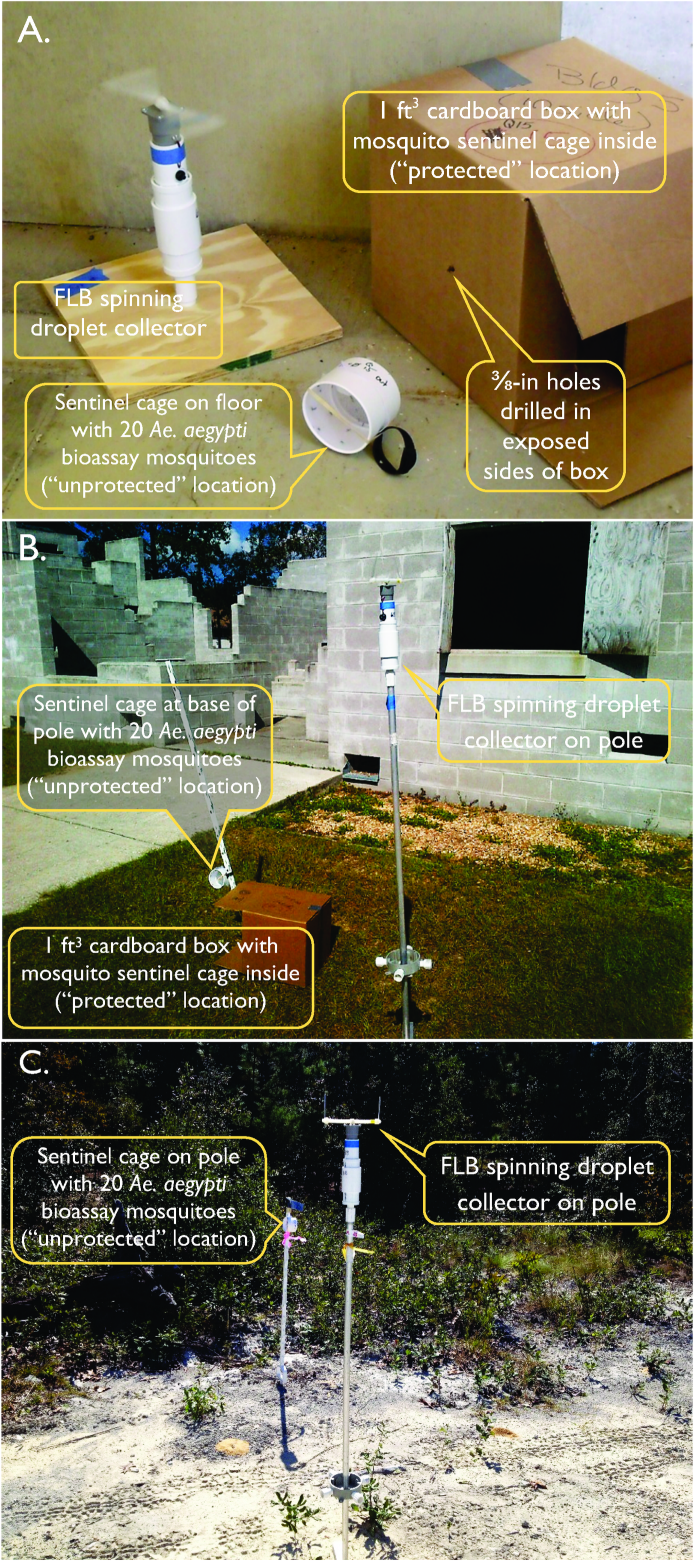
**Example of indoor (A), outdoor (B), and road Transect (C) arrangements of adult *Aedes aegypti* sentinel cages with adjacent FLB spinners**. Indoor and outdoor examples were photographed at MOUT South structures. “Protected” sentinel mosquito locations consist of 1 ft^3^ cardboard boxes with ⅜-in holes drilled in all exposed sides, with the boxes left partially opened and placed on their sides. “Unprotected” locations consist of placement of sentinel mosquito cages indoors directly on the floor of buildings, or outdoors at the base of a tread-in post.

We dispersed sentinel cages throughout the locations indicated in the MOUT South and Village inset maps in Fig 1 using the system of indoor and outdoor sentinel cage positions shown in S1 and S2 Tables. Each sentinel cage position consisted of two sentinel cages, one unprotected and one protected. At each sentinel cage position indoors, we placed one cage unprotected on the floor and one cage protected in a 1 ft^3^ partially opened cardboard box laying on its side with 3/8-in holes drilled through the center of each side and the bottom (Fig 3A). At each outdoor sentinel cage position we placed one cage unprotected approximately 3-in off the ground at the foot of a plastic Sentinel Tread-In Post (Jeffers; Dothan, AL) and one cage protected in a box as described for indoor locations (Fig 3B). With certain exceptions, detailed below, each of the 17 buildings marked in the Fig 1 MOUT and Village inset maps had one sentinel position indoors at the ground floor level and one sentinel position outdoors within a few meters of the front door. The sentinel cages placed in the open on the floor or near the ground on a pole measured efficacy of naled reaching unprotected locations indoors or outdoors, respectively. Cages in boxes measured efficacy in protected locations, simulating refugia such as the interior of closets or spaces under furniture for indoor locations, or simulating refugia in vegetation or within human-placed objects such as discarded appliances or containers for outdoor locations.

As indicated in Fig 1, selected buildings at the MOUT site were left open and some were partially sealed by closing shutters and doors. Indoor sentinel cage positions were located on both the first and second floors in the American Hotel and the Holiday Hotel, but only on the first floors of the remaining buildings. One additional location at the MOUT site, the Bridge, was selected for outdoor sentinel cages with a slight variation in exposure consisting of a sentinel cage position beneath the bridge and another sentinel cage position on top of the bridge.

At the Village site, indoor sentinel cage positions were located on both the first and second floors of the Main Structure, but only on the first floors of the remaining buildings (Fig 1). The outdoor locations at the Main Structure and Building No. 8 consisted of 8 ft high walled courtyards, but the outdoor locations at the remaining structures were open yards with no walls. One additional location at the Village site, the Roundabout, was selected for placement of an outdoor sentinel cage position.

Thus for each site, this layout of sentinel cages provided 10 cages indoors protected in boxes, 10 indoors exposed on the floor, 10 outdoors in boxes, and 10 outdoors exposed on poles–for a total of 80 sentinel cages across the MOUT and Village sites for each spray trial.

Along each of the two road Transect sites, one transecting MOUT South and one transecting the Village site, we placed 5 upwind and 5 downwind sentinel cages spaced approximately 350 ft apart as detailed in S3 Table and Fig 1. We placed each sentinel cage approximately 48-in above the ground at the top of a tread-in post (Fig 3C). No cages were placed at ground level or in boxes at the Transect sites. Thus for the Transect sites, this layout of sentinel cages provided a total of 20 cages outdoors unprotected on poles for each spray trial.

At the upwind Control site (Fig 1), we placed 10 sentinel cages at 5 outdoor locations spaced approximately 10 ft apart. Each Control location consisted of a tread-in post with a sentinel cage approximately 3-in off the ground at the foot of the post and another sentinel cage at the top of the post approximately 48-in off the ground. Sentinel cages at the Control site were handled in the same manner as those in treated areas.

We initiated deployment of uniquely labeled sentinel cages at the 4 treatment sites and the Control site approximately 30 min before each spray application, and marked each cage with the number of pre-spray dead or indicated zero if none dead at the time of placement. The number of pre-spray dead was subtracted from total dead prior to analysis of mortality data. The first swath of each spray set began within 10 min of completion of deployment of sentinel cages on both days. We left sentinel cages undisturbed for 1 hr post-spray and then collected and marked each cage with the number dead at 1 hr, and supplied each cage with a fresh 5% sucrose solution-soaked cotton ball. All post-spray cages were handled with disposable gloves to prevent cross-contamination. We returned sentinel cages to the CMAVE laboratory in designated post-spray coolers (separate coolers for treatment and Control sites) and transferred cages from coolers to plastic trays stored at room temperature and humidity to later record mortality at 4 hr and 12 hr post-spray. Treated and control mosquitoes were left in original field-exposed sentinel cages throughout the experiment.

### Droplet capture

We intended to evaluate technical efficacy of the aerial application by collecting pesticide droplets with ⅛-in x 3-in Teflon^®^-coated acrylic rods mounted on Florida Latham-Bonds (FLB) [24] slide spinners positioned adjacent to selected sentinel mosquito locations at the 4 treatment sites and the Control site (Figs 1 and 3; selected locations indicated in S3 and S4 Tables). These narrow acrylic rods are used to improve the very poor collection efficiency of small droplets (i.e., <20 um in diameter) observed when using standard 1-in x 3-in microscope slides [24,25]. We used the DropVision system (Leading Edge Associates, Inc.; Fletcher, NC) to measure droplet densities and droplet spectra of droplets captured on the rods. The spread factor [26] for Dibrom Concentrate was supplied by the manufacturer to correct the flattened drop to a sphere for accurate measurement. At the MOUT South site, FLB spinners were positioned outdoors and indoors on the first floors of selected buildings and on the roof of the Holiday Hotel. At the Village site, spinners were positioned outdoors and indoors at selected buildings and on the roof of the Main Structure. Indoor spinners were placed on mounts at floor level (slides held ~15-in from the floor) approximately 1 ft from sentinel cages. Outdoor spinners were placed at the top of 5 ft poles approximately 4 ft from sentinel cages. Spinners were positioned adjacent to selected sentinel cages on both Transect sites, and adjacent to selected sentinel cage positions at the Control site. In parallel with timing of sentinel mosquito cages, we designated specific teams of personnel to deploy and retrieve FLB spinner sampling rods. These personnel used disposable gloves and custom-designed sampling rod storage boxes to retrieve, store, and transport rods without disturbing droplets or cross-contaminating materials. Care was taken to minimize production of airborne dust near spinners during sample periods.

### Data analysis and mapping

All adult *Ae*. *aegypti* sentinel mosquito mortality data from treatment areas were normalized with Abbott’s formula [27] using mortality data from control areas. Mortality and droplet density data were uploaded to georeferenced sentinel cage positions in ArcGIS 10.3 (ESRI; Redlands, CA) and processed with inverse distance weighting to produce interpolated surfaces representing spray efficacy [23,28]. Basic and comparative statistical data were derived from all corrected mortality data and droplet collection data using Wizard 1.8 (Evan Miller, http://www.wizardmac.com; Chicago, IL). We designed these statistical comparisons to evaluate the relative efficacy of 8001 and 8003 nozzles across an array of combinations of indoor and outdoor protected and unprotected locations. Statistical (*t*-test) comparisons of Abbott-corrected 1 hr and 4 hr mortality were also conducted to derive a measure of both knockdown and long-term efficacy of the aerial naled treatments. Where 1 hr and 4 hr mortality were not significantly different, this would indicate maximum mortality, or knockdown, at 1 hr post-spray; i.e., the peak mortality that could be expected with that formulation under the given conditions took place relatively quickly. On the other hand, increasing values of significant difference between the 1 hr and 4 hr mortality would indicate diminishing knockdown coupled with increasingly greater long-term effects; i.e., the pesticide could be considered to have a delayed peak effect under the given conditions. Finally, we intended to analyze mortality and droplet data with linear regression to determine the extent droplet density could predict target insect mortality.

## Results

### Pesticide spray and meteorology

Dibrom Concentrate was applied over the MOUT, Village, and road transect field sites on 29 and 30 October 2013 with the C-130H aerial ULV platform with the parameters listed in Table 1. Meteorological conditions at release height, rooftop level, and ground level, and spray timing for both days, are presented in Table 2. Rooftop level on the Holiday Hotel was approximately 40 ft AGL, which was comparable to treetop level in the treatment area. Conditions were generally warm and mild, with winds mostly under 9 mph and stable from the northeast permitting a series of 6 adjacent upwind swaths at both sites on both days. These were operationally reasonable environmental conditions for actual aerial mosquito control.

**Table 1:** Aerial ULV spray application parameters.

Application Date	29 October	30 October
**Nozzles**	2 booms, 12 SS**8001** each boom oriented straight down	2 booms, 4 SS**8003** each boom oriented straight down
**Application Rate**	0.84 fl oz/A (0.086 lb A.I./A)	1.00 fl oz/A (0.100 lb A.I./A)
**Spray System Flow Rate**	1.50 gal/min	1.81 gal/min
**Acres treated** **(total volume naled applied)**	911 A (5.9 gal)	890 A (7.0 gal)
**Aircraft Speed**	230.2 mph (200 knots)	230.2 mph (200 knots)
**Swath Width**	500 ft	500 ft
**Offset of First Swath**	50 ft (MOUT South)	175 ft (MOUT South)
	150 ft (Village)	275 ft (Village)
**Release Height**	150 ft (AGL)	150 ft (AGL)

These parameters are from the 29–30 October 2013 experimental trials over the MOUT South, Village, and road transect sites, Camp Blanding, Florida. AGL = above ground level. Offset of First Swath indicates distance from the first upwind structure in each simulated populated area to the edge of the first of the six swaths applied to each target area.

**Table 2:** Spray timing and meteorology at release height.

				Release Height 150 ft AGL		Rooftop (MOUT) ~40 ft AGL				MOUT South (Wax Candle) Ground Level				Village (Roundabout) Ground Level			
Site	Pass no.	Spray duration (sec)	Time of spray	Wind Dir. (°)	Wind Speed (knots)	Wind Dir. (°)	Wind Speed (knots)	Temp. (°F)	%RH	Wind Dir. (°)	Wind Speed (knots)	Temp. (°F)	%RH	Wind Dir. (°)	Wind Speed (knots)	Temp. (°F)	%RH
**MOUT** **South** **(29 Oct)**	1	20	1253 h	064°	6	052°	6.7	80.0	48	358	3.0	76.6	64.6	-	-	-	-
	2	21	1257 h	062°	6	060°	10.0	80.6	48	62	5.0	77.2	62.3	-	-	-	-
	3	20	1259 h	059°	6	058°	6.2	80.7	48	62	3.0	79.6	58.6	-	-	-	-
	4	19	1304 h	055–077°	7	070°	6.1	81.1	47	36	5.0	79.6	58.7	-	-	-	-
	5	20	1307 h	080°	7	116°	7.7	78.7	49	85	3.6	82.2	54.4	-	-	-	-
	6	20	1312 h	065°	6–8	059°	5.9	81.0	48	51	1.3	81.5	55.0	-	-	-	-
**Village** **(29 Oct)**	1	20	1322 h	060–070°	8–9	057°	5.2	81.4	47	-	-	-	-	289	3.9	78.2	56.8
	2	18	1326 h	062–070°	6	069°	7.2	82.4	45	-	-	-	-	359	2.5	79.7	54.3
	3	20	1330 h	063–075°	7	047°	4.5	82.4	44	-	-	-	-	36	1.2	79.8	54.3
	4	20	1333 h	075°	7	074°	6.4	81.5	45	-	-	-	-	55	3.5	78.9	53.4
	5	19	1337 h	063–080°	7	061°	8.0	81.4	43	-	-	-	-	105	2.8	81.9	50.0
	6	18	1341 h	068°	7	062°	7.2	82.3	44	-	-	-	-	38	3.2	79.9	52.5
**MOUT** **South** **(30 Oct)**	1	21	0954 h	045°	9–10	054°	3.1	74.2	59	56	1.8	74.1	72.7	-	-	-	-
	2	21	0958 h	053°	7	080°	4.6	75.4	58	61	5.2	73.1	74.4	-	-	-	-
	3	18	1002 h	055°	7	055°	4.0	75.5	56	83	1.7	74.2	72.4	-	-	-	-
	4	21	1006 h	054°	7	055°	5.5	75.1	55	76	3.3	73.9	71.2	-	-	-	-
	5	20	1009 h	050–055°	8–11	062°	4.8	75.7	55	71	3.0	73.8	71.6	-	-	-	-
	6	21	1013 h	046–051°	9–10	082°	6.4	76.2	55	82	4.3	73.9	71.2	-	-	-	-
**Village** **(30 Oct)**	1	18	1025 h	054–058°	5	072°	2.4	77.5	53	-	-	-	-	56	2.5	75.3	68.5
	2	20	1028 h	060°	4	054°	5.4	75.9	53	-	-	-	-	28	3.0	75.2	69.2
	3	19	1031 h	057–064°	3	054°	4.3	76.8	53	-	-	-	-	71	4.3	75.4	69.6
	4	18	1035 h	054°	6	064°	5.4	76.6	54	-	-	-	-	72	3.4	75.8	68.7
	5	14	1038 h	054°	6	084°	6.3	77.6	54	-	-	-	-	342	5.0	74.9	70.3
	6	16	1042 h	054°	6	075°	6.5	78.5	53	-	-	-	-	167	1.7	76.7	67.1

Data are from MOUT South Holiday Hotel rooftop level (approx. 40 ft AGL), and at ground level at each site during aerial ULV sprays conducted 29–30 October 2013 at Camp Blanding, Florida. Wind direction is in degrees relative to magnetic north; %RH = percent relative humidity. AGL = above ground level.

### Caged sentinel mosquito mortality

Sentinel adult *Ae*. *aegypti* mosquito mortality data from treatment areas for 29–30 October are summarized statistically in Tables 3 and 4; full data are presented in S1–S3 Tables. Sentinel mosquito mortality was not significantly different between 4 hr and 12 hr at any location, so only 12 hr data are presented in Table 3. For both 29 and 30 October, mortality was not significantly different between open and closed buildings at MOUT South, nor between buildings with and without courtyards at the Village site. Therefore sentinel mosquito mortality data were pooled across these location categories at each site prior to statistical comparisons. Mean mortality at 12 hr post-spray in untreated control sentinel *Ae*. *aegypti* mosquitoes was 1.5% on 29 October and 2.9% on 30 October and these values were used to normalize mortality in treatment areas with Abbott’s formula.

**Table 3:** Statistical (*t*-test) comparisons of Abbott-corrected 12 hr mortality.

Site	Location		29 Oct (8001)	30 Oct (8003)	29 Oct *vs*. 30 Oct
**MOUT South**	indoors *vs*. outdoors	in box	~32% reduction indoors*	N.S.	-
		on floor/pole	~66% reduction indoors***	~25% reduction indoors*	-
	box *vs*. floor/pole	indoors	N.S.	N.S.	-
		outdoors	~55% reduction in box***	~37% reduction in box**	-
	all indoors *vs*. all outdoors		~49% reduction indoors***	N.S.	-
	indoors	in box	-	-	~34% higher on 30 Oct*
		on floor	-	-	~41% higher on 30 Oct*
	outdoors	in box	-	-	N.S.
		on pole	-	-	N.S.
	all indoor		-	-	~38% higher on 30 Oct***
	all outdoor		-	-	N.S.
	all indoor + outdoor		-	-	~23% higher on 30 Oct**
**Village**	indoors *vs*. outdoors	in box	~40% reduction indoors*	N.S.	-
		on floor/pole	~27% reduction indoors***	N.S.	-
	box *vs*. floor/pole	indoors	~51% reduction in box***	~85% reduction in box***	-
		outdoors	~37% reduction in box**	~77% reduction in box***	-
	all indoors *vs*. all outdoors		~33% reduction indoors**	N.S.	-
	indoors	in box	-	-	N.S.
		on floor	-	-	~21% higher on 30 Oct**
	outdoors	in box	-	-	~40% higher on 29 Oct*
		on pole	-	-	N.S.
	all indoor		-	-	N.S.
	all outdoor		-	-	N.S.
	all indoor + outdoor		-	-	N.S.
**MOUT *vs*. Village**	indoors	in box	N.S.	~39% higher at MOUT**	-
		on floor	~39% higher at Village**	N.S.	-
	outdoors	in box	N.S.	~39% higher at MOUT*	-
		on pole	N.S.	N.S.	-
	all indoor		~24% higher at Village*	N.S.	-
	all outdoor		N.S.	N.S.	-
	all indoor + outdoor		N.S.	N.S.	-

Mortality data are across locations at MOUT South and Village sites following 29–30 October 2013 aerial ULV applications of naled against adult sentinel *Ae*. *aegypti*. Mortality was not significantly different between open and closed buildings at MOUT South, nor between buildings with and without courtyards at the Village, so data were pooled across building types at each site. N.S., not significant (*P* > 0.05)

*, *P* ≤ 0.05

**, *P* ≤ 0.01

***, *P* ≤ 0.001.

**Table 4:** Statistical (*t*-test) comparisons of Abbott-corrected 1 hr and 4 hr mortality.

Site	Location		29 Oct (8001)	30 Oct (8003)
**MOUT South**	indoors	in box	N.S.	~33% higher at 4 hr*
		on floor	~20% higher at 4 hr*	~63% higher at 4 hr***
	outdoors	in box	N.S.	~57% higher at 4 hr***
		on pole	~29% higher at 4 hr*	N.S.
	all indoor		~12% higher at 4 hr**	~48% higher at 4 hr***
	all outdoor		N.S.	~38% higher at 4 hr**
	all indoor + outdoor		~20% higher at 4 hr*	~43% higher at 4 hr***
	**Transect near MOUT**	on pole	~56% higher at 4 hr***	N.S.
**Village**	indoors	in box	N.S.	N.S.
		on floor	~57% higher at 4 hr***	~56% higher at 4 hr***
	outdoors	in box	N.S.	N.S.
		on pole	~42% higher at 4 hr**	N.S.
	all indoor		~36% higher at 4 hr***	~29% higher at 4 hr*
	all outdoor		~33% higher at 4 hr*	N.S.
	all indoor + outdoor		~34% higher at 4 hr***	N.S.
	**Transect near Village**	on pole	~34% higher at 4 hr*	~15% higher at 4 hr**
**MOUT** **& Village Combined**	indoors	in box	N.S.	~18% higher at 4 hr*
		on floor	~39% higher at 4 hr***	~60% higher at 4 hr***
	outdoors	in box	~25% higher at 4 hr*	~34% higher at 4 hr**
		on pole	~35% higher at 4 hr***	~14% higher at 4 hr*
	all indoor		~24% higher at 4 hr***	~38% higher at 4 hr***
	all outdoor		~30% higher at 4 hr**	~24% higher at 4 hr*
	all indoor + outdoor		~27% higher at 4 hr***	~31% higher at 4 hr***
	**Transects Combined**	on pole	~44% higher at 4 hr***	~11% higher at 4 hr**

Data are across locations at MOUT South, Village, and Transect sites following 29–30 October 2013 aerial ULV applications of naled against adult sentinel *Ae*. *aegypti*. This analysis provides a measure of both knockdown and long-term efficacy of the aerial naled treatments. Mortality was not significantly different between 4 hr and 12 hr mortality at any location, thus comparisons limited to 1 hr vs. 4 hr mortality. N.S., not significant (*P* > 0.05) indicates maximum mortality at initial 1 hr knockdown

*, *P* ≤ 0.05

**, *P* ≤ 0.01

***, *P* ≤ 0.001 indicate diminishing knockdown coupled with increasingly greater long-term effects.

Ants were present in mosquito sentinel cages in several locations indicated in S1 and S2 Tables at the time of cage retrieval with variable numbers of sentinel mosquitoes missing. After observing the behavior of ants and surviving mosquitoes in these cages and noting that (a) remaining mosquitoes were all alive in cases where fewer than 20 mosquitoes were present with ants, (b) several cages with ants had all 20 mosquitoes present, and (c) ants did not interact with the living mosquitoes, we hypothesized that ants had removed only dead mosquitoes and the number missing was equal to the number of mosquitoes killed by the pesticide application.

**Fig 4:**
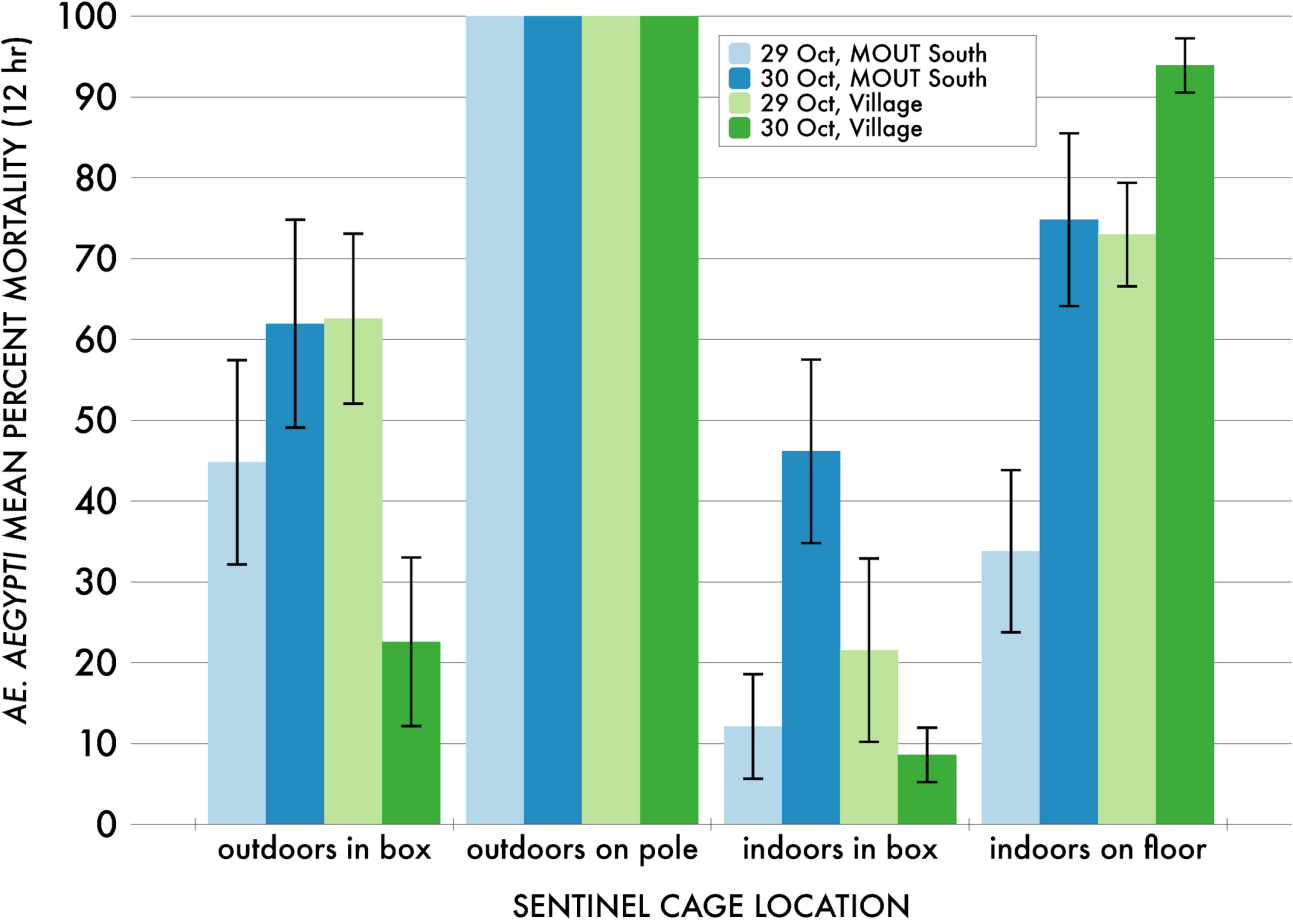
Mean 12 hr sentinel *Ae*. *aegypti* percent mortality in MOUT South and Village outdoor and indoor protected (“in box”) and unprotected (“on pole” or “on floor”) locations across both days. Non-zero mortality values in all locations across both days confirmed that the aerially applied material reached throughout the MOUT South and the Village sites and penetrated buildings and experimental refugia. Each histogram for each of the four cage location types at each site for each trial was based on percent mortality data from 20 sentinel cages.

Mean 12 hr sentinel *Ae*. *aegypti* mortality along both Transect sites and in most MOUT South and Village outdoor unprotected locations across both days was very high (94.9–100%; Fig 4) and confirmed that the aerially applied material reached throughout the MOUT South and the Village sites. However, mean 12 hr mortality across the MOUT South and Village sites in outdoor protected (i.e., in-box) locations (22.6–62.6%) and in both protected (8.6–46.2%) and unprotected (33.8–93.9%) locations indoors was generally lower and highly variable across both days (Fig 4), but confirmed that pesticide penetrated buildings and experimental refugia on both days.

**Fig 5:**
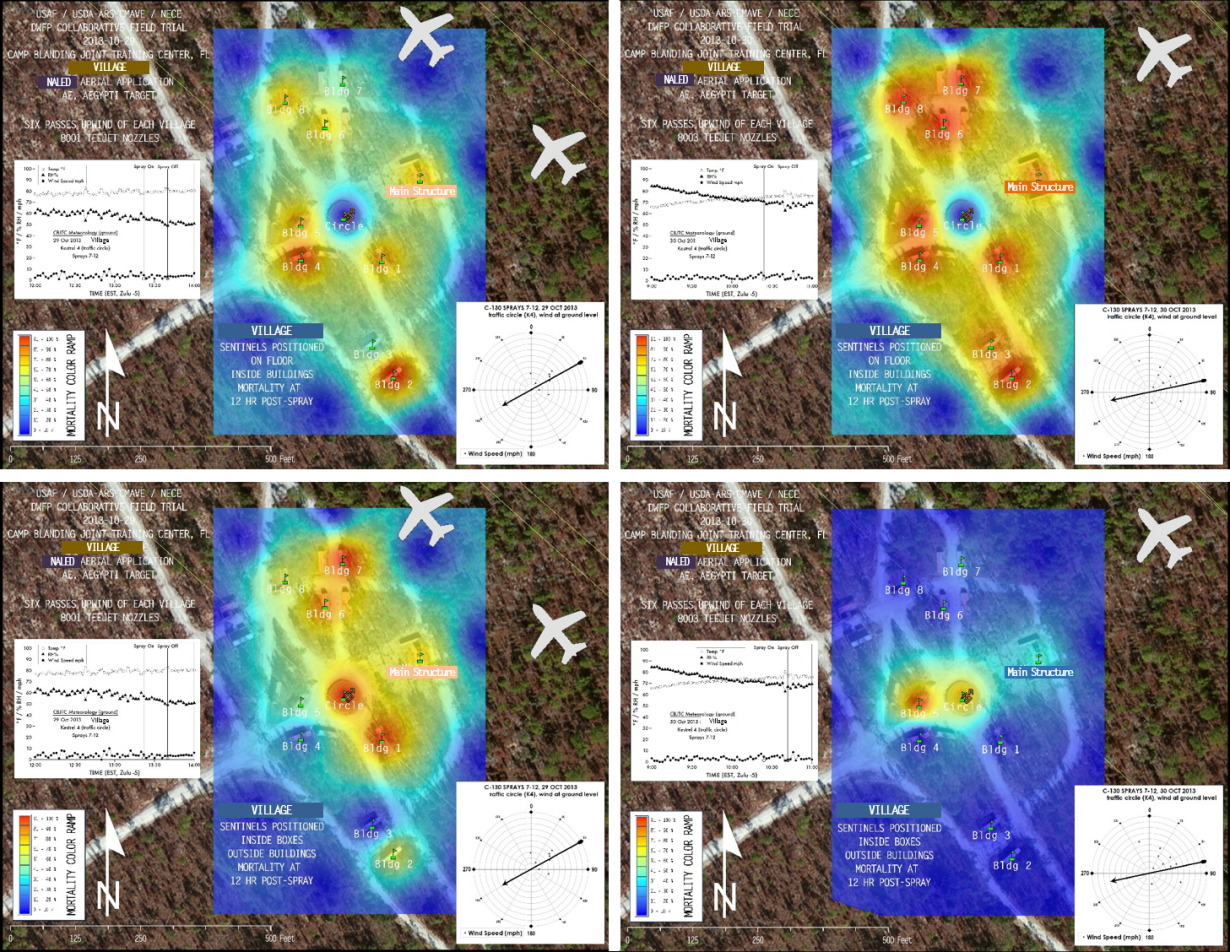
Efficacy maps of 12 hr interpolated mortality data. These maps represent spray efficacy for (A) indoor unprotected (“outside boxes”) and (B) outdoor protected (“inside boxes”) sentinel cage locations at Village on (left) 29 October (8001 nozzles) and (right) 30 October (8003 nozzles). These interpolated “heat maps” show a range of mortality from very low (blues and light greens) to very high (oranges and reds). The aircraft silhouette symbol shows the actual size, position, and flightpath of the first upwind swath of the C-130H relative to the map scale. Additional efficacy maps of interpolated mortality data for all sentinel cage locations in all trials are shown in Figure Sets A–D in S1 Fig. Maps were created using ArcGIS software by Esri under license as described in S2 File.

Comparing the 29 October and 30 October applications (Table 3) compares results from 8001 and 8003 nozzles, respectively, with some caveats. Application rates were lower with the 8001 nozzles (0.84 fl oz/A) on 29 October, which was an artifact of equipment limitations not present with the larger 8003 nozzles (1.0 fl oz/A) on 30 October (Table 1). Also, the timing of the applications was different each day: the 29 October application took place 1253–1341 h; whereas the 30 October application took place about 3 h earlier 0954–1042 h, which could potentially have meant differences in atmospheric stability (turbulence) in addition to the meteorological variations summarized in (Table 2). Unstable, turbulent conditions are primarily created by the sun’s heating of the surface and peak in the afternoon hours [29]. Thus the 29 October application could have been challenged by more turbulent conditions compared to 30 October, which could have disproportionately affected smaller droplets by moving them away from the target areas. Indeed, mortality patterns on 29 October with the smaller droplet spectrum and lower flow rate associated with the 8001 nozzles could suggest a lower penetration of indoor locations and simulated refugia locations at both sites compared to the 30 October application with the 8003 nozzles. However, although the overall efficacy of the 8003 application at MOUT South across all sentinels was ~23% higher than the 8001 application there, there was no significant difference between the overall efficacy of the 8001 and 8003 applications at the more open Village site. Additionally, although there was overall ~21% higher penetration of indoor unprotected locations at the Village site with the 8003 application compared to the 8001 application (Fig 5A), the 8001 application showed ~40% higher efficacy in outdoor protected locations at this site (Fig 5B). Additional efficacy maps of 1hr, 4 hr, and 12 hr interpolated mortality data, similar to those shown in Fig 5, representing spray efficacy for all sentinel cage locations in all trials are shown in Figure Sets A and B in S1 Fig (MOUT South) and Figure Sets C and D in S1 Fig (Village).

Overall efficacy across all sentinel locations was not significantly different at MOUT South versus Village sites within each treatment day (Table 3). However, efficacy in all indoor unprotected locations at the Village site was ~39% higher than similar locations at MOUT South on 29 October with the 8001 nozzles. Conversely, efficacy in all protected locations indoors and outdoors at MOUT South was ~39% higher than similar locations at the Village site on 30 October with the 8003 nozzles.

### Knockdown

Mortality was not significantly different between 4 hr and 12 hr within each location category (i.e., indoors in box, indoors on floor, outdoors in box, or outdoors on pole), thus knockdown analysis was limited to 1 hr compared to 4 hr mortality. Knockdown (i.e., no significant differences between 1 hr and 4 hr mortality) was observed on 29 October with the 8001 nozzles at both indoor and outdoor protected locations at both the MOUT South and Village sites (Table 4), although mortality in these location types was generally much lower than unprotected locations (Table 3; S1 Table). Knockdown was also observed on 29 October at MOUT South when all outdoor mortality data were pooled, and when mortality data at all indoor protected locations were pooled across both MOUT South and the Village site (Table 4). On 30 October with the 8003 nozzles, knockdown was observed in outdoor unprotected locations at MOUT South and the nearby Transect. On 30 October at the Village site knockdown was observed in indoor and outdoor protected locations, as well as outdoor unprotected locations, and in pooled data across all outdoor sentinels and across all outdoor and indoor sentinels combined (Table 4). In all other location types and location groupings at both sites across both days, a range of delayed effects was observed, indicated by significantly higher mortality at 4 hr compared to 1 hr (0.05 ≥ *P* ≤ 0.001).

### Droplet densities, droplet spectra

Droplet data including the droplet spectrum for each location partitioned into DV_10_, DV_50_ (the volume median diameter, or VMD), and DV_90_ which denote, respectively, the 10%, 50%, and 90% partitions of droplet sizes across the observed spectrum, are shown in S4 and S5 Tables. Droplet collecting rods from all sites on 29 October and rods from the Village site and nearby Transect on 30 October were analyzed within 2 hr post-spray. However, most of the rods from the 30 October application over the MOUT South site and nearby Transect were read 24 hr later on 31 October. Comparison of 31 October re-analyses of the few rods analyzed within 2 hr of the 30 October application over the MOUT South site, i.e., Wax Candle indoor and outdoor, Holiday Hotel Roof B, and R18, indicated that the VMD (i.e., DV_50_) had decreased from 7.5–8.0 μm to 5.9–6.6 μm and that the DV_90_ had decreased from 12.0–15.0 μm to about 9.8–11.5 μm. These changes in diameter suggest shrinkage of droplets, most likely from evaporation in storage [30]. Droplet data in S4 and S5 Tables from the MOUT Site and nearby Transect for 30 October are presented from the 31 October analysis, and therefore likely underestimate droplet size at the time of application by 1.5–3.5 μm. Similar to the interpolated mortality data represented in Fig 5, interpolated droplet density data and mapped bar graphs of droplet densities for all locations in all trials are shown in Figure Sets E–F in S1 Fig (MOUT South) and Figure Sets G–H in S1 Fig (Village).

The mean VMD and droplet densities across both Transect sites were 5.8 μm and 16.7 droplets/mm^2^ (d/mm^2^) on 29 October and 6.4 μm and 34.5 d/mm^2^ on 30 October. The mean VMD and droplet densities across both MOUT South and Village sites were 5.9 μm and 13.5 droplets/mm^2^ on 29 October and 6.3 μm and 29.3 droplets/mm^2^ on 30 October for all indoor locations and 5.9 μm and 24.5 droplets/mm^2^ on 29 October and 6.5 μm and 47.3 droplets/mm^2^ on 30 October for all outdoor locations. Control site mean VMD and droplet densities were 5.9 μm and 19.2 droplets/mm^2^ and 6.4 μm and 28.9 droplets/mm^2^ on 29 and 30 October, respectively. Thus, the mean VMD and droplet density values measured at the Control site nearly 5 mi upwind of the last spray swath each day (Fig 1) were not significantly different from those for each corresponding day across all outdoor FLB spinners in the treatment areas (S4 and S5 Tables). Consequently, unlike the Abbott-corrections conducted to normalize sentinel mosquito mortality, we did not conduct corrections of droplet capture data relative to droplets collected at the Control site because it would have resulted in unrealistic droplet values such as zero or negative values throughout the data set. We concluded that there was a concentration of airborne pollutant droplets–for example, from power plants, factories, or engine exhaust from the nearby state highway–at the Control site that was not distinguishable from the likely combination of similar airborne pollutant droplets with droplets of applied pesticides in the treatment areas. In this study we did not add fluorescent tracer dye to the naled so there was no way to differentiate these two droplet populations on the slides, so we did not analyze mortality and droplet data with linear regression to determine the extent droplet density could predict target insect mortality.

## Discussion

In this study we investigated the capability of naled (Dibrom) applied from a fixed wing ULV spray platform in a warm temperate ecological zone to penetrate indoor and outdoor protected and unprotected habitat harboring adult *Ae*. *aegypti* mosquitoes. We found that these aerial applications of naled resulted in nearly 100% mortality in sentinel caged mosquitoes placed in outdoor unprotected locations and it had varying capability to penetrate opened or partially-sealed buildings or artificial refugia placed within these buildings or outdoors. For the most part, the patterns of mortality across both the MOUT South and Village sites were not unexpected: indoor locations tended to have lower mortality compared to outdoor locations; both indoor and outdoor protected locations tended to have lower mortality than unprotected locations; and indoor efficacy was higher at Village site structures compared to those at the MOUT South, because buildings were smaller and with larger openings at the Village site (Tables 3 and 4; S1–S3 Tables).

Despite some capability to penetrate structures in this study, there are several potential limitations that should be considered if planning real-world aerial adult *Ae*. *aegypti* control operations with naled. The first consideration brings forward the habitat setting and meteorology of the study sites. The structures and immediately adjacent roadways in both the MOUT South and Village sites were reasonable simulations of some types of dwellings in dengue-endemic regions. The Village site, for example, could represent some small real-world villages that are positioned within canopy habitat. However, the habitat immediately surrounding the MOUT South site consisted of pine-dominated forest canopy which could have presented a different form of challenge to pesticide drift than real-world urban areas with road and alley corridors among structures over a much larger area. Depending on meteorology at the time of application in a real-world urban area, road and alley corridors could provide channels for natural air movements to draw pesticide plumes around and through buildings [31] in contrast to forest canopy which may interrupt plume movement [32]. On the other hand, heat trapped in the air surrounding buildings in urban areas could produce atmospheric layers that may repel droplet plumes and potentially reduce penetration. Meteorological conditions in dengue-endemic areas (for example those documented in [12]; Table 5) could be expected to be warmer and more humid than the mild autumnal weather recorded during the sprays at CBJTC, which also favored our experimental trials with winds consistently perpendicular to the spray line. Also, possible turbulence related to the time of day that may have contributed to the differences in efficacy observed between the 29 October and 30 October applications could present a different regime of effects on ULV plumes depending on humidity and temperature in the tropics and subtropics, compared to temperate Florida.

**Table 5:** Results compared across the 3 known fixed wing aerial application studies targeting sentinel adult *Ae*. *aegypti* with naled or dichlorvos.

								Abbott-corrected adult *Ae*. *aegypti* mortality averaged across all comparable sentinels in each study					
								Outdoor			Indoor		
Study	Conditions	Location	Spray system	Flight	Formulation	Application	Swath	4 hr	12 hr	24 hr	4 hr	12 hr	24 hr
**Merk et al. 1971 [16]**	11 Aug 1970 Morning 61°F 72%RH wind 4.5–5.3 mph (3.9–4.6 kt)	Illarsaz, Switzerland	Pilatus Porter PC-6 Micronair AU 3000	125 ft AGL 107 mph (93 kt)	Dichlorvos, 95% technical grade	3.6 fl oz/A VMD ~48 μm r 0.73/mm^2^	656 ft 4 swaths	93.3%	-	-	75.3%	-	-
**US CDC 1987 [12]**	28 Jul-8 Aug 1987 0600–0930 h 73–91°F *%RH not available* wind 6.9–10.3 mph (6–9 kt)	San Juan, Puerto Rico	C-130A IPSS Wing booms 8005 (28 Jul) 8003 (5–8 Aug)	150 ft AGL 230 mph (~200 kt)	Dibrom 14 (naled, 85%)	1 fl oz/A VMD unknown r unknown	1000 ft multiple swaths	-	-	94.2%	-	-	71.0%
**Present study**	29–30 Oct 2013 0945–1345 h 73.1–82.2°F 50.0–74.4%RH wind 1.4–5.8 mph (1.2–5.2 kt)	Camp Blanding Joint Training Center, Starke, Florida	C-130H MASS Fuselage booms 8001 (29 Oct) 8003 (30 Oct)	150 ft AGL 230 mph (~200 kt)	Dibrom Concentrate (naled 87.4%)	0.84–1 fl oz/A VMD unknown r unknown	500 ft 12 swaths	97.7%	100%	-	61.4%	68.9%	-

Droplet VMD and density (r) and indoor and outdoor Abbott-corrected mortality values are averaged across all measurements within each study. To make the studies more comparable, mortality data from the present study do not include protected locations indoors or outdoors because neither of the other studies placed sentinels in protected locations. Meteorological data are presented as a range across all applications within each study. Meteorological data were not published in [12] and so were obtained from the National Centers for Environmental Information at https://www.ncdc.noaa.gov/cdo-web/search. Meteorological data for [16] and the present study were recorded locally at the time of application.

Another consideration concerns the limitations of the simulated dwelling structures. All structures at MOUT South, including those partially closed, and the Village were well ventilated with permanent openings and thus highly permeable. In fact, mortality was not significantly different between open and closed buildings at MOUT South. Similarly, mortality and droplet data suggested that courtyard walls at the Village site did not hinder movement of pesticide (S2 and S4 Tables), which could have moved into building openings at an angle not inhibited by the 8 ft courtyard walls. Future studies could investigate whether a truck or other ground level application could be more impeded by these walls. Although we used small cardboard boxes in an attempt to simulate indoor and outdoor adult mosquito refugia, real-world refugia would present substantially greater challenge. For example, although some boxes were placed in closet nooks present in some MOUT South structures, these nooks did not have closet doors and no clothing was present, and other indoor boxes were near main doors or other points of ventilation. This scenario is unlike typical closed-up households in dengue-endemic areas that would present a much more elevated challenge to penetration. In field trials in an earlier related study, even hand-fogging inside closed structures in Thailand did not provide100% control of sentinel adult *Ae*. *aegypti* placed in simulated refugia [33].

The limitations of the experimental design should also be taken into account. The trials on both days were conducted with multiple passes of adjacent swaths intended to partially simulate an operational application such as the one described in [11] so it is not possible from this study to assess the efficacy of a single pass, nor to identify the effective offset required for each pass under the given meteorological conditions and surrounding habitat. We strived to keep spray parameters similar despite changes in nozzle size and the constantly changing environmental conditions across the two spray days. However, the different flow rates shown in Table 1 reflect limitations of available equipment at smaller droplet spectra produced by 8001 nozzles resulting in 1.1 gal more material being applied over 21 fewer acres on the second day with the 8003 nozzles. Also, guidance from the AgDISP system more than tripled the offset of the first swath to 175 ft at MOUT South and nearly doubled the offset to 275 ft at the Village site on 30 October with the 8003 nozzles (Table 1). Thus there were different application factors each day which means that the two trials should not be considered replicates for absolute comparison of the two nozzle sizes; rather, our trials provided a range of potential efficacies given the habitat, nozzle sizes, and meteorology. This is the standard problem with aerial spray trials that are separated by more than just a few minutes.

Another important consideration is that the sentinel adult mosquitoes used in the study were colony-reared, highly bottlenecked, and susceptible organisms from a lineage more than 60 yr old. Patterns of mortality in these mosquitoes, though providing an experimental baseline of efficacy, likely do not adequately represent the capability of aerially applied naled against natural populations of adult *Ae*. *aegypti*. Regardless of adult sentinels used in any study, real-world aerial adulticiding may suppress some percentage of the current adult *Ae*. *aegypti* population but must be coupled with an IVM program including source reduction and larviciding to offset recruitment of adult mosquitoes from untreated areas and development of new cohorts from unaffected larvae within the treated area will soon repopulate the treated area [34].

Some incidental potential experimental errors consisted of ant presence in some cages, in particular on the 30 October trial (S1 and S2 Tables), and the sugar-water cotton ball falling off of some cages. For the ant presence, we made the decision to simply calculate mortality based on a count of living mosquitoes. We observed that ants did not attempt to catch or otherwise interact with living mosquitoes in these cages. However, the larger species seen, possibly scavenger types, were observed in some cases cutting up mosquitoes that were already dead or moribund so that they could carry them off. The majority of ants seen were smaller species, evidently attempting to feed on the sugar water in the cotton balls attached to sentinel cages. Some cages on pick up were found to have lost their sugar-water cotton balls, but we hypothesized that this had a very low or zero contribution to mortality. All sentinel mosquitoes had been stored in coolers with abundant hydration and nutrition, were exposed to the mild outdoor environment for fewer than 90–120 minutes, and were returned to coolers before storage in a lab environment for mortality checks. Cages that had lost cotton balls but had zero mortality at pick up and at 12 hr (data not shown) demonstrate that this was not a relevant factor in mortality in this study.

Indoor and outdoor efficacy observed in the present study are comparable to results from the two earlier known fixed wing aerial application studies targeting sentinel adult *Ae*. *aegypti* with naled or dichlorvos (Table 5). Considering the available and comparable data across the three studies, however, we are somewhat limited in how we may analyze their similarities. We have to ignore the mortality from cages placed within boxes indoors and outdoors in the present study because the 1971 and 1987 studies do not indicate that cages were placed within protected locations indoors or outdoors. The 1971 study reports only 4 hr mortality, and the 1987 study reports only 24 hr mortality. Data are reported per sentinel cage for a single trial in the 1971 study but only as a mean across all sentinel cages for each of five treatment dates in the 1987 study. We used the indoor and outdoor means of the mortality data from the single trial in the 1971 study and indoor and outdoor means across the five treatment dates from the 1987 study to compare to mean indoor and outdoor mortalities from the present study. We used the reported control mortality data from the 1971 and 1987 studies to conduct Abbott correction of their mortality data, because neither of these prior studies applied this correction. Spray parameter and meteorological differences are present among the three studies, yet the outcomes in terms of indoor and outdoor efficacy are reasonably comparable (Table 5). One notable difference is the higher indoor efficacy at 4 hr in the 1971 study, possibly because the application rate was 3.6 times higher per acre, with a potentially more insecticidally active [35] compound; i.e., DDVP which is only present in the atmosphere after a delay during a naled application, because oxidation of naled to DDVP has to take place first [15]. On the other hand, the outdoor efficacy in the 1971 study was the lowest of the three studies which may suggest that DDVP, with the property of rapid vaporization, is more effective when trapped in more sheltered locations, and may account in part for the efficacy of naled in indoor and outdoor protected locations observed in the present study.

Recording mortality frequencies at different time periods post-spray allowed us to investigate the relative capability of naled to induce rapid knockdown across increasing levels of protected environments in the treatment area. At the MOUT South area on 29 October (8001 nozzles) it may at first seem counterintuitive that there was a delayed effect with maximum mortality after 4 hr in unprotected locations both indoors and outdoors, yet a more stable effect of maximum mortality after only 1 hr in protected locations indoors and outdoors. It could be the case that indoor and outdoor unprotected areas receive higher cumulative doses, but the process of accumulation consists of a series of smaller doses received over time as each swath is applied and pesticide drifts into the target area, delaying mortality in the majority of exposed individuals. Each more distant swath contributes less and less to this accumulation but the smallest droplets may drift the farthest and nevertheless add up.

It should be noted that although the process of knockdown as we have defined it means that there is maximum mortality by 1 hr, this maximum for a particular location may still be significantly lower than the 4 hr maximum mortality at another location that took hours longer to produce but is ultimately a much higher mortality. Similarly, we can observe significantly higher mortality at 4 hr, yet the percentage mortality at 1 hr was already high, such as observed at outdoor sentinels along both road Transect sites (Table 4; S3 Table). However, in protected locations indoors and outdoors, the significant difference between 1 hr and 4 hr tells a different story, because the 1 hr mortality was very low or zero while the 4 hr mortality was statistically significantly greater but still very low compared to sentinels in, say, outdoor unprotected areas (Table 4: 30 October, MOUT South). On the other hand, some protected locations showed little difference between 1 hr and 4 hr mortality, suggesting that if there was any mortality at all it would happen right away (Table 4: 29 and 30 October, Village).

Protected locations may only really get a single dose consisting of a portion of the first swath that is substantially smaller than the dose an unprotected location is exposed to from that same swath. In this scenario, the subsequent second through sixth more distant swaths do not contribute enough droplets to penetrate the protected locations, compared to unprotected locations that continue to receive some exposure from subsequent swaths leading to higher overall mortality in unprotected areas. Future trials with single swath applications could provide more clarity on this potential process.

Future studies should also investigate the possibility that pesticide borne in droplets is not the only mode of delivery of pesticide to target insects. It is well known that evaporation from droplets takes place as they move through the atmosphere from their point of production [36]. The vapor produced from the droplets must consist of the solvent (and possibly other inactive ingredients), the active ingredient or its derivatives, or a combination of all the volatile constituents of the formulation. Other processes that affect droplets and vapor products include the oxidation or other reaction of the active ingredient with compounds already present in the atmosphere such as CO_2_, or physical breakdown for instance from UV radiation from sunlight [37]. Certainly, naled is known to break down in the air and soil into vaporous DDVP [15,38] which is highly effective against mosquitoes. Chemical analysis by [39] indicated no naled present on screening of sentinel cages placed 10 m from a ground thermal fog application of naled at 157 g ai/hectare. Similarly, malathion, another widely used ULV formulation, oxidizes to malaoxon [40] which may also be active as a vapor [41] potentially toxic to mosquitoes in the treatment area. In earlier ground-based ULV field experiments we have directly observed sentinel mortality in areas where dye-labeled pesticide (surveyed with a spectrophotometer from droplet capture ribbons) as well as non-labeled pesticide active ingredient (surveyed with GC/MS from droplet capture ribbons) were not detected (SCB, KJL, RLA unpubl. data).

While adult mosquito sentinels in outdoor unprotected locations may be more affected by the liquid (droplet) phase of aerosol pesticides, the vapor phase of the pesticide, or vaporous products of its oxidation, may have a greater contribution to mortality in sentinels in more sheltered areas such as indoor locations or locations within small refugia. In protected locations, the droplets or vapor that do penetrate are more likely to linger, because of restricted air flow, and though much reduced compared to unprotected areas, will kill some relatively smaller part of the sentinel population. In unprotected locations, droplets (and/or vapor) will be in higher quantity but be more subject to being carried away by wind currents and replaced by fresh air which will keep some individuals alive longer, but due to accumulation of decreasing concentrations as the swaths are applied but at increasing distances away, will eventually die as their defensive metabolic capabilities are worn down. For example, sentinels at the outdoor transect near MOUT South on 29 October (8001 nozzles and lower application rate) ultimately exhibited 100% mortality, but this was on average 56% less at 1 hr across sentinels at this location (Table 3). On the other hand, at this same location on 30 October (8003 nozzles and higher application rate) we also observed 100% mortality but this occurred as rapid knockdown within 1 hr. Mortality on 30 October in indoor protected and unprotected locations at MOUT South was significantly higher than 29 October (Table 3) yet with maximum mortality only reached at 4 hr,

We had initially hypothesized that the smaller VMD (8001 nozzles, 29 October) would be more effective at reaching indoor or sheltered areas, yet the 30 October MOUT South mortality data potentially support a hypothesis that an initially larger VMD and droplet density (8003 nozzles) could reach sheltered areas more effectively–possibly because ~16% more material was available for production of a vapor phase. Another possibility is that an initially larger VMD makes the pesticide cloud more robust (less likely to be dispersed or overshoot) as it travels to the target area and ultimately more small droplets are produced because larger droplets become small droplets as they evaporate; whereas, a cloud with a smaller initial VMD could evaporate to vapor and disperse before it reached the target area. Additionally, the time-of-day differences in the applications could have meant that more atmospheric turbulence and resultant off-target drift was present during the afternoon application, possibly compounding with the effects of smaller droplets evaporating more quickly and resulting in overall lower efficacy on 29 October in these indoor sheltered areas at MOUT South. Sequential placement in time and space of sentinels and droplet collection apparatus in future investigations could provide information on the relative contribution of timing and accumulation of droplet density, and dynamics of droplet evaporation, to knockdown compared to long-term efficacy.

Regarding aerial ULV measures against *Ae*. *aegypti* in the United States, peridomestic populations of *Ae*. *aegypti* may be more prevalent than *Ae*. *aegypti* living inside homes because dwellings tend to be more closed off from the outdoors compared to dwellings for instance in dengue-endemic areas such as Puerto Rico (but see: [42], dengue epidemic in a small Florida town with high *Ae*. *aegypti* presence inside homes). Efforts to reach indoor protected locations may not be as critical in US areas such as Florida because most homes are closed against the outside environment, with screens and air conditioning in use throughout the day with only incidental incursion of *Ae*. *aegypti* adults. Also, US homes tend not to have indoor standing water. The apparent positive contribution to the reduction of *Ae*. *aegypti* by aerial ULV applications of naled in Miami in late 2016 in response to focal transmission of Zika virus [43] suggests that naled is reaching, at least, outdoor protected locations where *Ae*. *aegypti* are expected to be sequestered. The present study suggests that naled can reach the interior of a home when the structure is relatively open and situated in meteorological conditions favorable for aerial ULV application. Subsequent trials with other products containing different active ingredients should also be conducted for comparative efficacy purposes.

Although we did observe success penetrating indoor areas, this capability had been previously demonstrated and we did not generally observe higher efficacy compared to similar prior studies (Table 5). The present study in addition to [12,16] all indicate that levels of control in indoor or sheltered locations attained with aerial pesticide application should not be expected to match control in outdoor, exposed locations. Adulticiding of course should not be considered a sole recourse to reduce populations of medically important mosquitoes such as *Ae*. *aegypti*; any aerial or ground-based space spray control efforts against *Ae*. *aegypti* must be coupled with an array of IVM strategies, to include traditional as well as emerging and innovative technologies, for long-term, sustainable suppression of this species. Multiple (sequential) spray applications of naled from a C-130H or similar platform timed to best impact adult mosquitoes based on local population dynamics appears to be an effective component of an IVM campaign against *Ae*. *aegypti*.

Finally we should consider for aerial operations the differences in what it means to attempt to control an endophilic species with focal population structure such as *Ae*. *aegypti* compared to targeting vast, sylvatic, and highly mobile and delocalized populations of mosquitoes such as *Ae*. *taeniorhynchus* or *Psorophora columbiae*. Unlike mosquito species traditionally targeted by operational mosquito control, activity patterns of *Ae*. *aegypti* are not as tied to photoperiod, wind currents, or temperature [44]. In one sense this attribute potentially makes traditional ULV spray periods such as dusk less important and frees us to conduct ULV operations when the conditions optimize ULV itself. Also, populations of *Ae*. *aegypti* are much smaller and geographically focused, and generally far more sequestered at any given time of day. *Aedes aegypti* spend more time resting and waiting for human presence and less time flying since they are already positioned, by nature, near their hosts when they emerge from immature habitat as adults. Because of this, the traditional model which requires ULV droplets to impinge on flying *Ae*. *aegypti* to be effective, may need to be expanded to include the possible contribution of vapor products from ULV reaching these mosquitoes at rest in indoor or outdoor cryptic refugia. Specifically targeting endophilic *Ae*. *aegypti* may also be enhanced by shortening the lane separation of the application from the typical 1000 ft to 500 ft, as in this study, to provide a downward push of droplets from the wake turbulence inherent from the C-130H aircraft, with the resulting energy potentially moving small droplets indoors.

## Supporting information

S1 Fig Naled efficacy (mortality) 29–30 October at MOUT South and Village sites and naled droplet densities 29–30 October at MOUT South, Village, and Transect sites. (PDF)

S1 Table Abbott-corrected sentinel adult *Ae*. *aegypti* mosquito percent mortality at 1, 4, and 12 hr post-spray following the 29–30 October aerial naled applications over the MOUT South site. Doors and windows were propped open at all structures unless indicated; Wax Candle and Bread Market structures had no doors or window shutters present and were left open. Percent mortality at 1 hr marked in bold with dagger indicates ants present at time of sentinel cage pickup.
(PDF)

S2 Table Abbott-corrected sentinel adult *Ae*. *aegypti* mosquito percent mortality at 1, 4, and 12 hr post-spray following the 29–30 October aerial naled applications over the Village site. All structures were unmodified with windows and doors completely open. Percent mortality at 1 hr marked in bold with dagger indicates ants present at time of sentinel cage pickup.
(PDF)

S3 Table Abbott-corrected sentinel adult *Ae*. *aegypti* mosquito percent mortality at 1, 4, and 12 hr post-spray following the 29–30 October aerial naled applications over the Transect sites. All Transect sentinels were placed outdoors unprotected in the open at the top of 48-in high poles.
(PDF)

S4 Table Droplet analysis data from the 29–30 October aerial naled applications over the MOUT South, Village, and control sites. The droplet spectrum for each location is partitioned into DV10, DV50 (VMD), and DV90. The symbol r denotes droplet density in droplets per mm^2^.
(PDF)

S5 Table Droplet analysis data from the 29–30 October aerial naled applications over the Transect and control sites (all outdoors, unprotected). The droplet spectrum for each location is partitioned into DV10, DV50 (VMD), and DV90. The symbol r denotes droplet density in droplets per mm^2^.
(PDF)

S1 File Supplementary references. (PDF)

S2 File Cite ArcGIS basemap. Esri guidance on citing basemaps and maps created in ArcGIS.
(PDF)
