# Supplementary material for: Aerial ULV control of Aedes aegypti with naled (Dibrom) inside simulated rural village and urban cryptic habitats
Source: PLoS One. 2018 Jan 19;13(1):e0191555. doi: 10.1371/journal.pone.0191555 (PMC5774805; doi:10.1371/journal.pone.0191555)

**SET A**

S1–S12 Figs. Naled efficacy (mortality), MOUT 29 Oct.

**Figure S1**
29 October 2013
8001 nozzles
MOUT South
1 hr mortality
Outdoors **IN BOX**

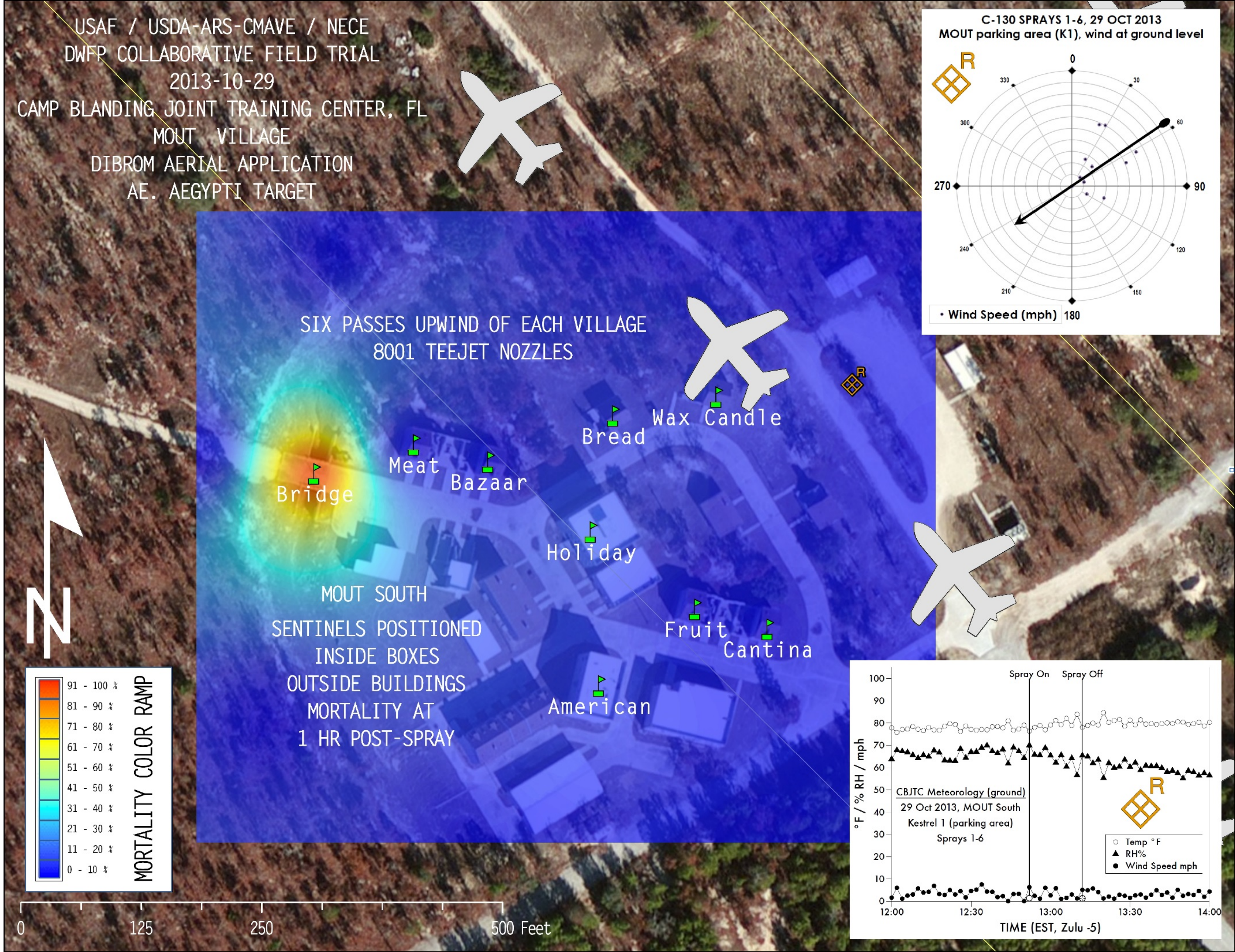

**Figure S2**
29 October 2013
8001 nozzles
MOUT South
4 hr mortality
Outdoors **IN BOX**

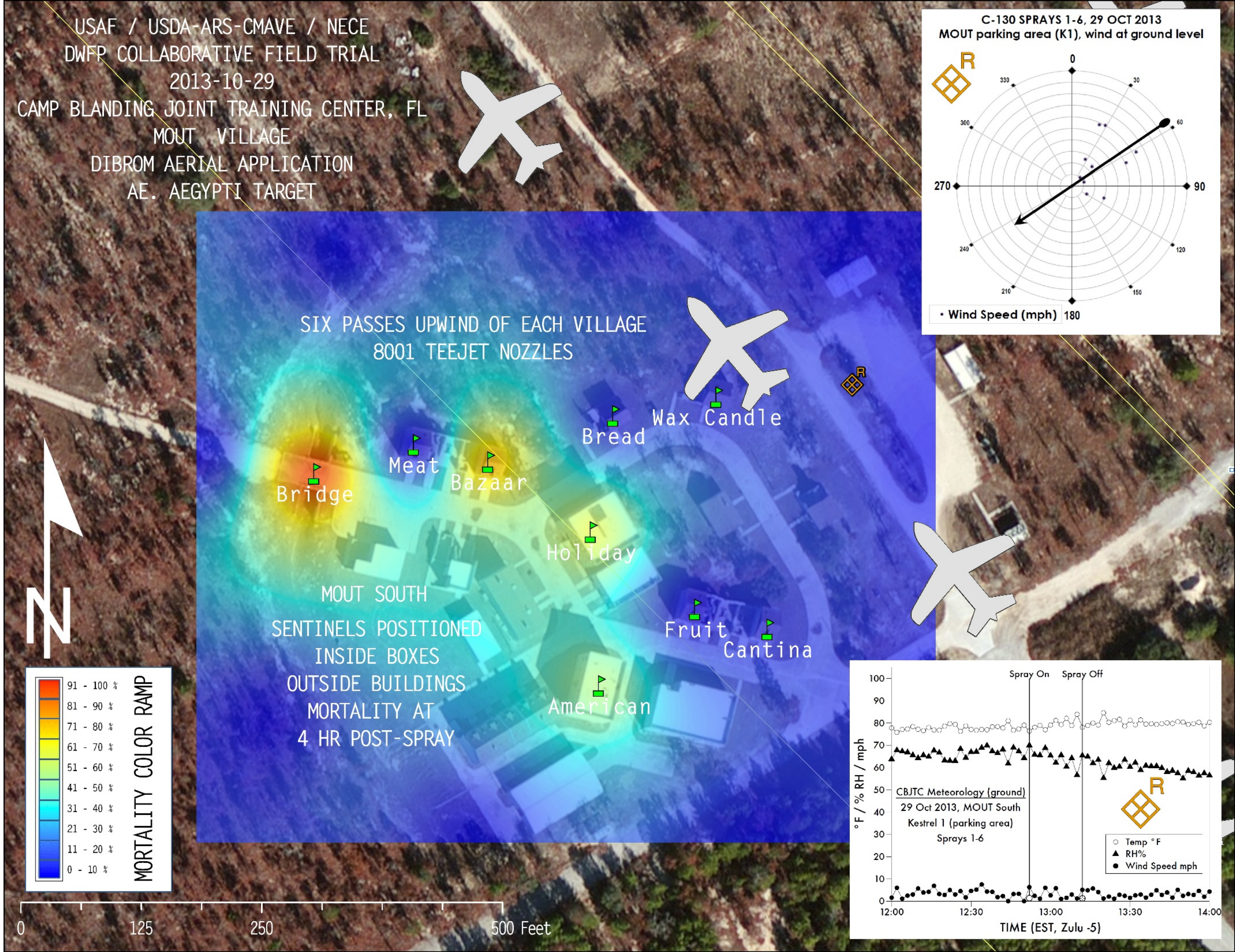

# Figure S3

29 October 2013

8001 nozzles

MOUT South

12 hr mortality

Outdoors **IN BOX**

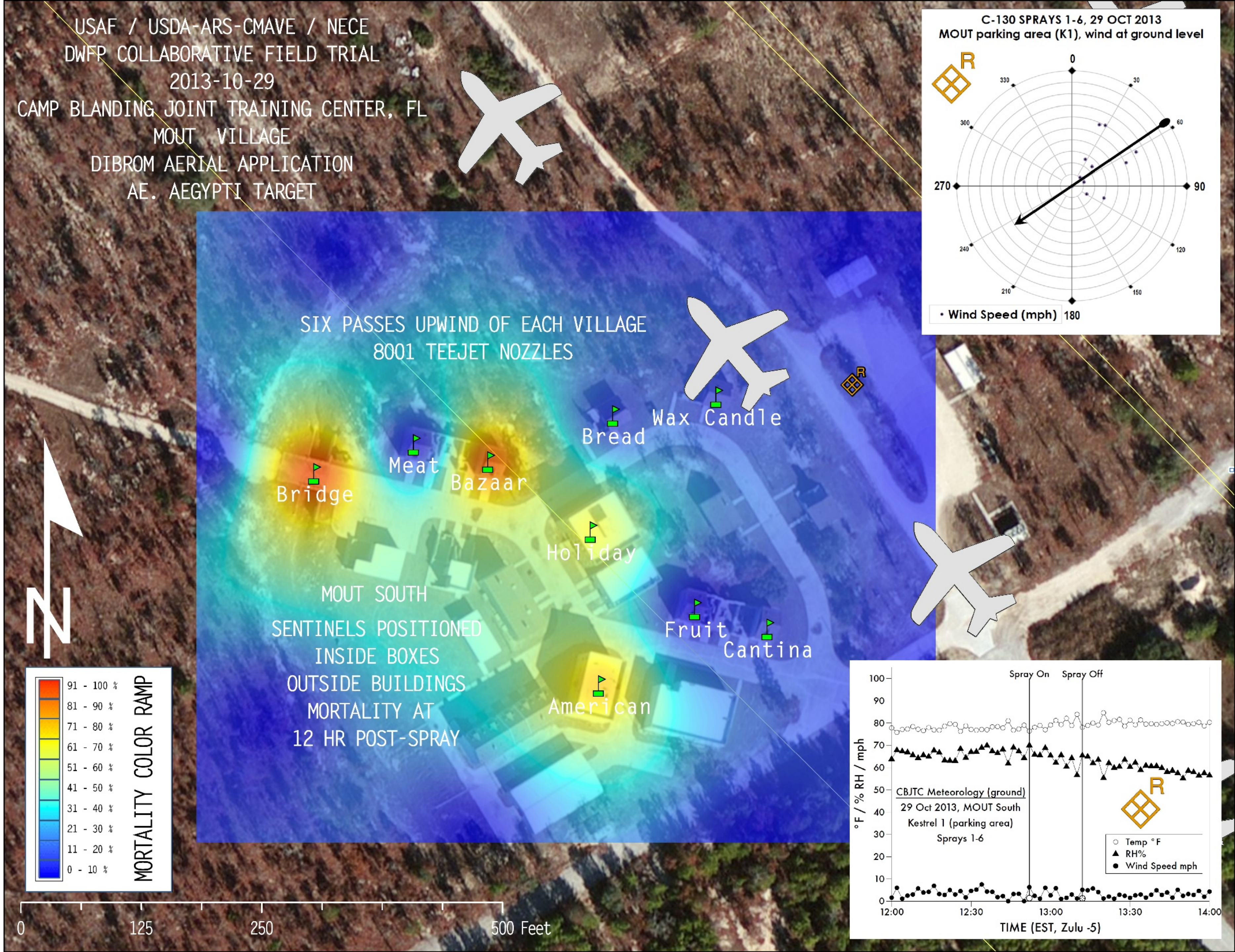

**Figure S4**
29 October 2013
8001 nozzles
MOUT South
1 hr mortality
Outdoors **ON POLE**

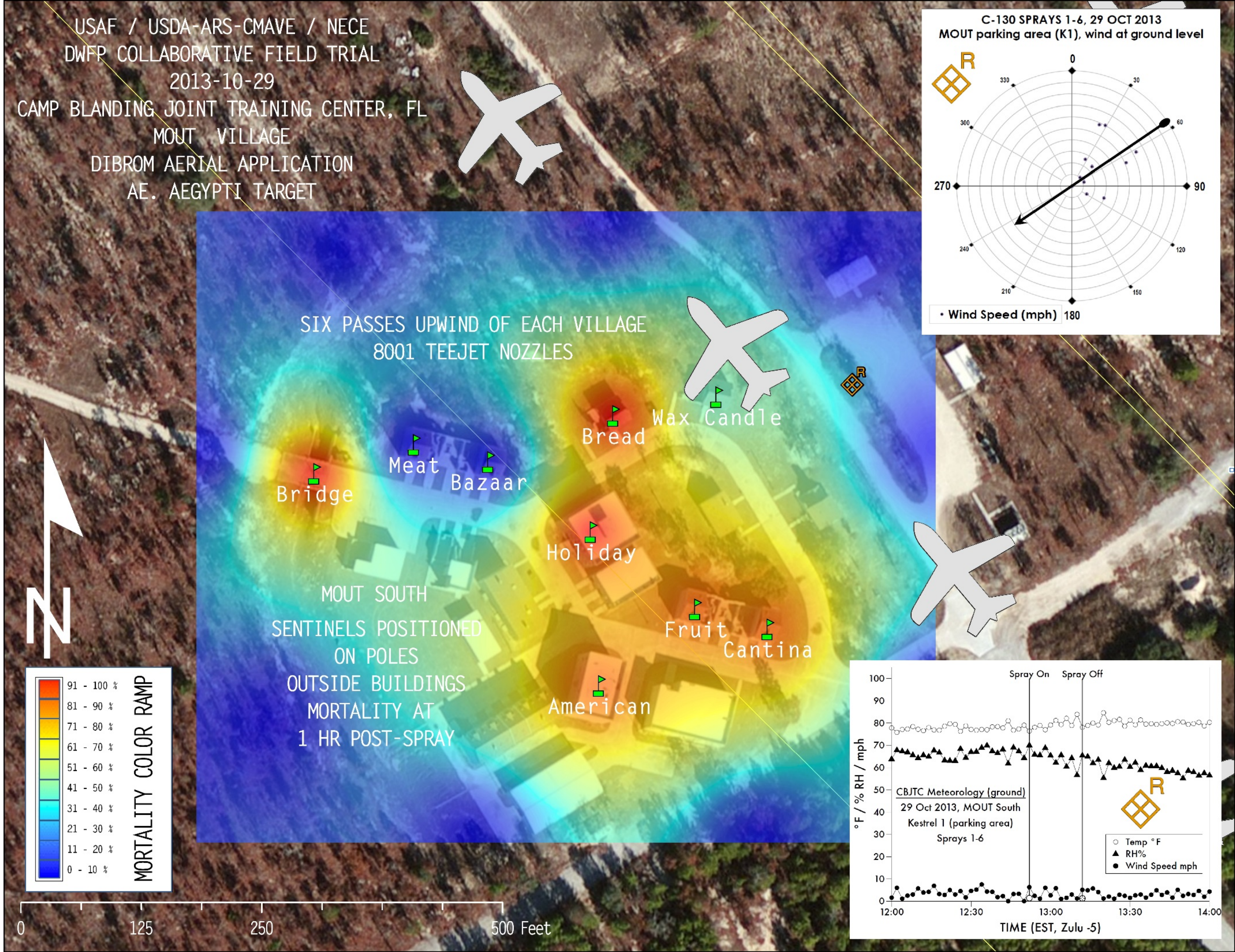

**Figure S5**
29 October 2013
8001 nozzles
MOUT South
4 hr mortality
Outdoors **ON POLE**

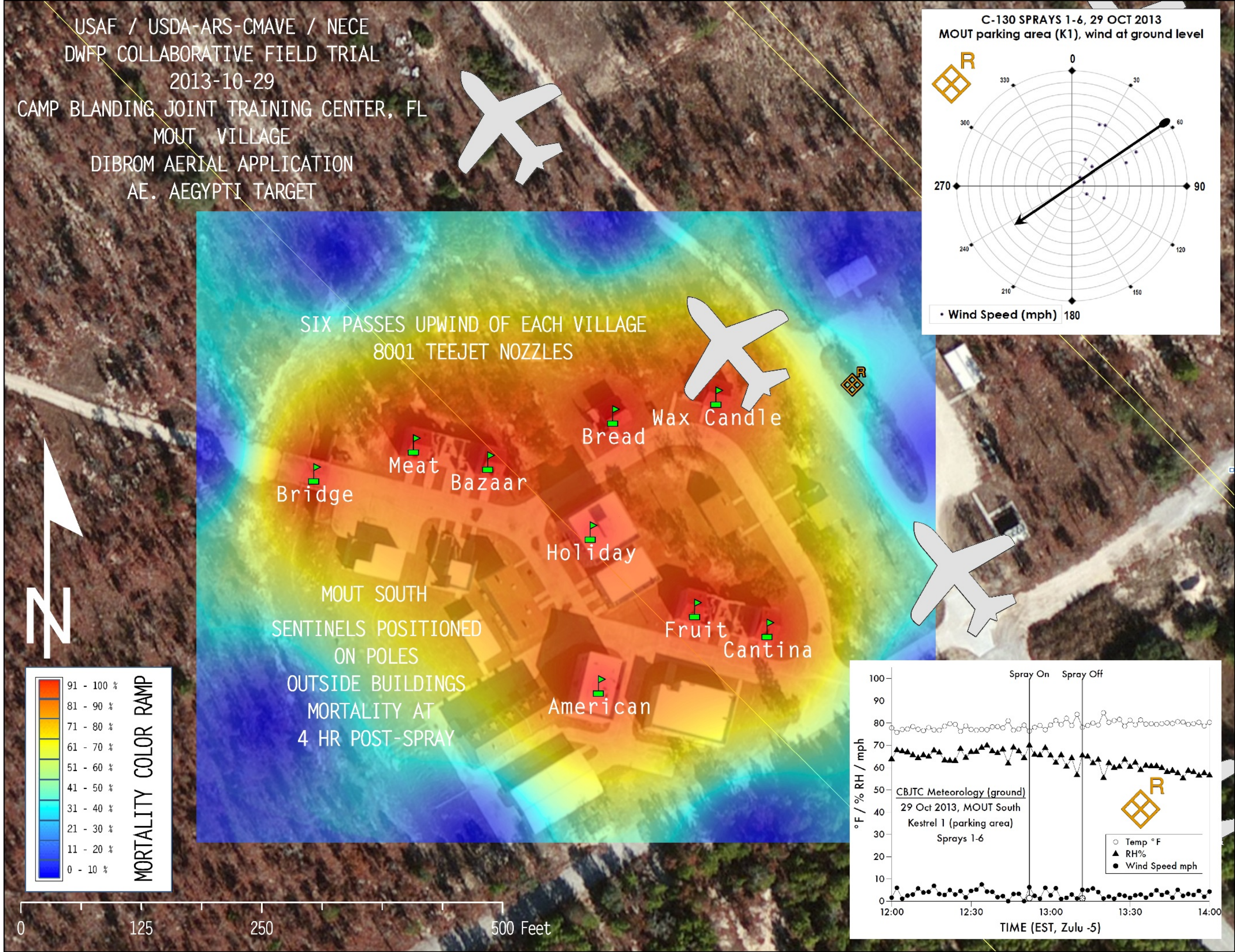

**Figure S6**
29 October 2013
8001 nozzles
MOUT South
12 hr mortality
Outdoors **ON POLE**

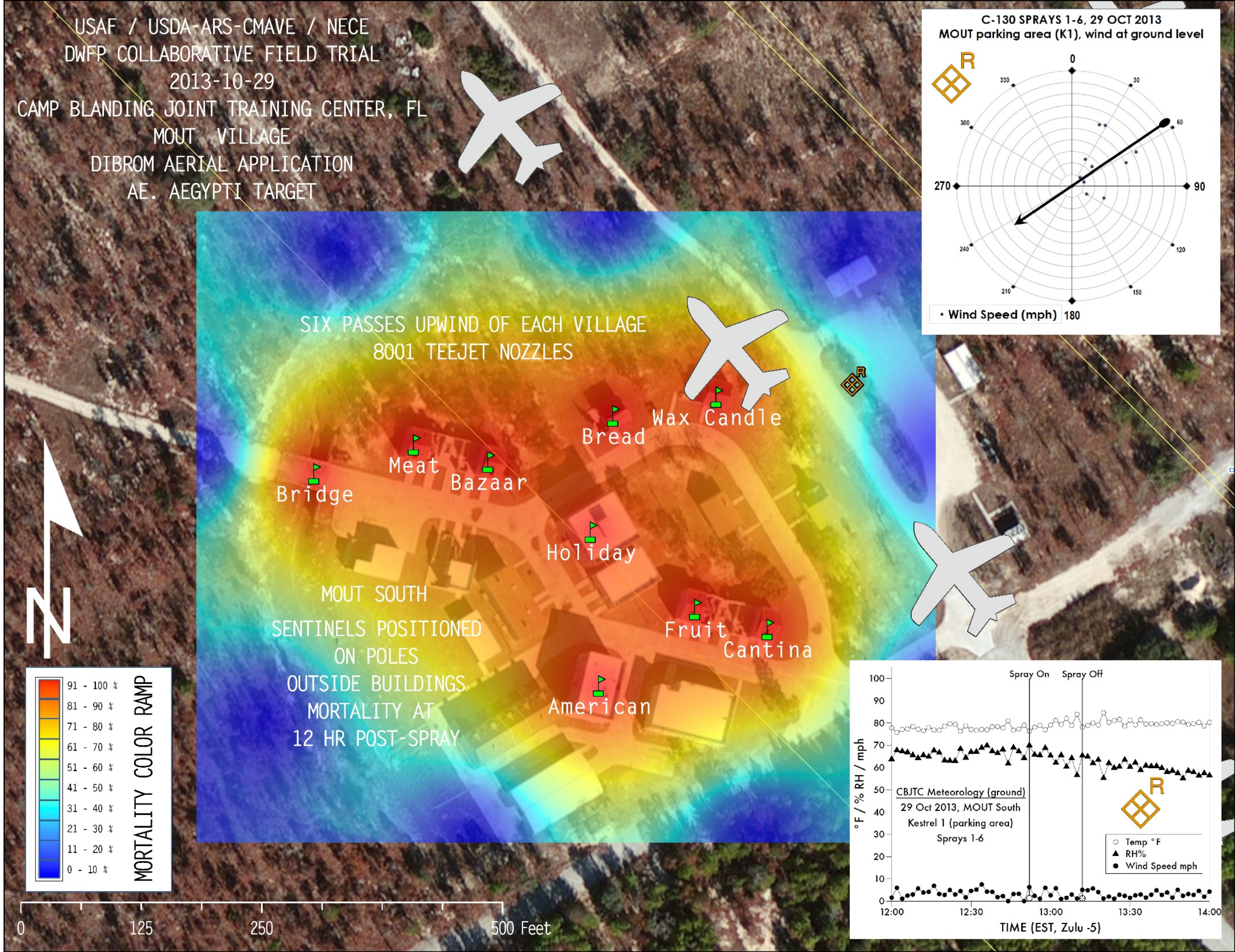

**Figure S7**
29 October 2013
8001 nozzles
MOUT South
1 hr mortality
Indoors **IN BOX**

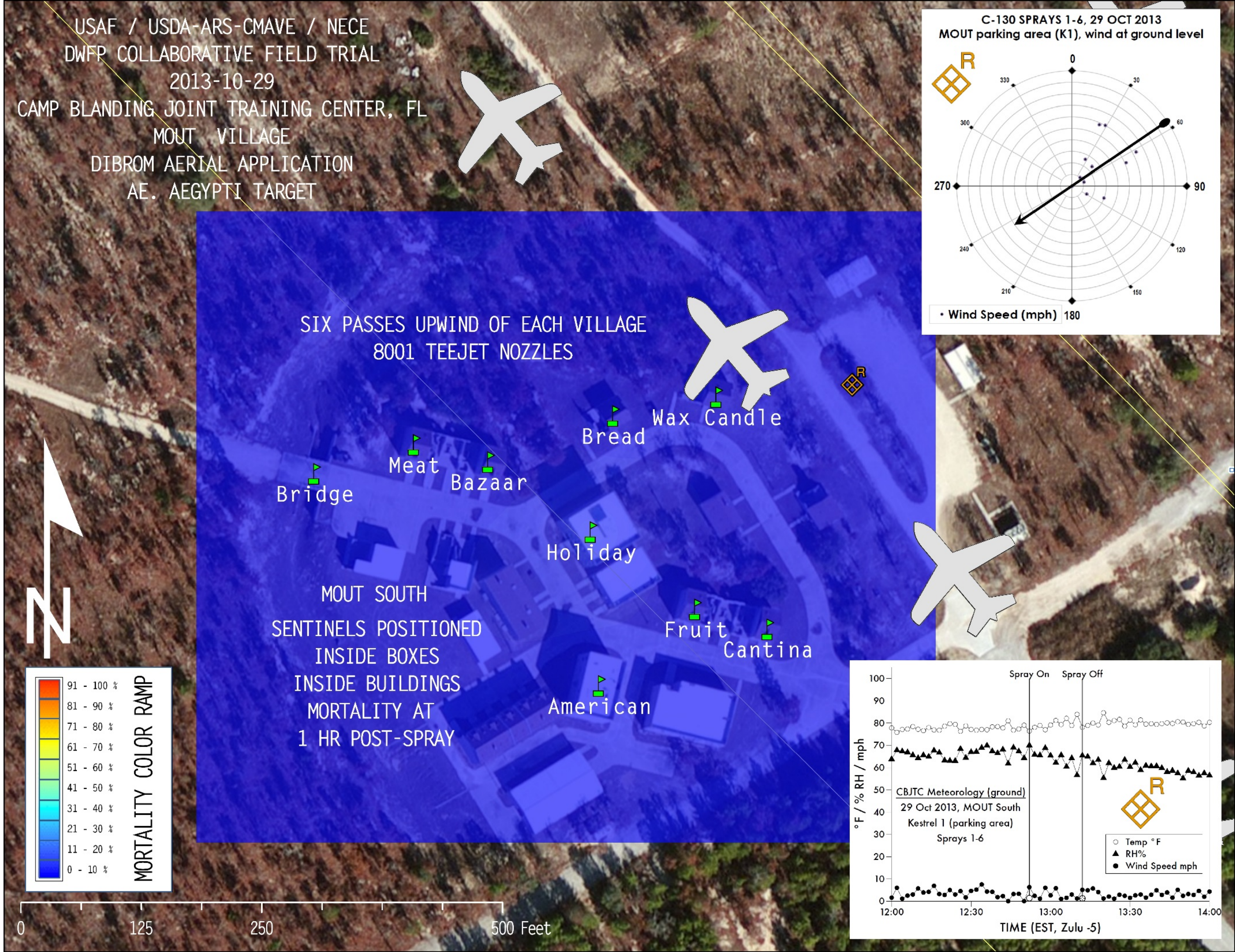

# Figure S8

29 October 2013

8001 nozzles

MOUT South

4 hr mortality

Indoors **IN BOX**

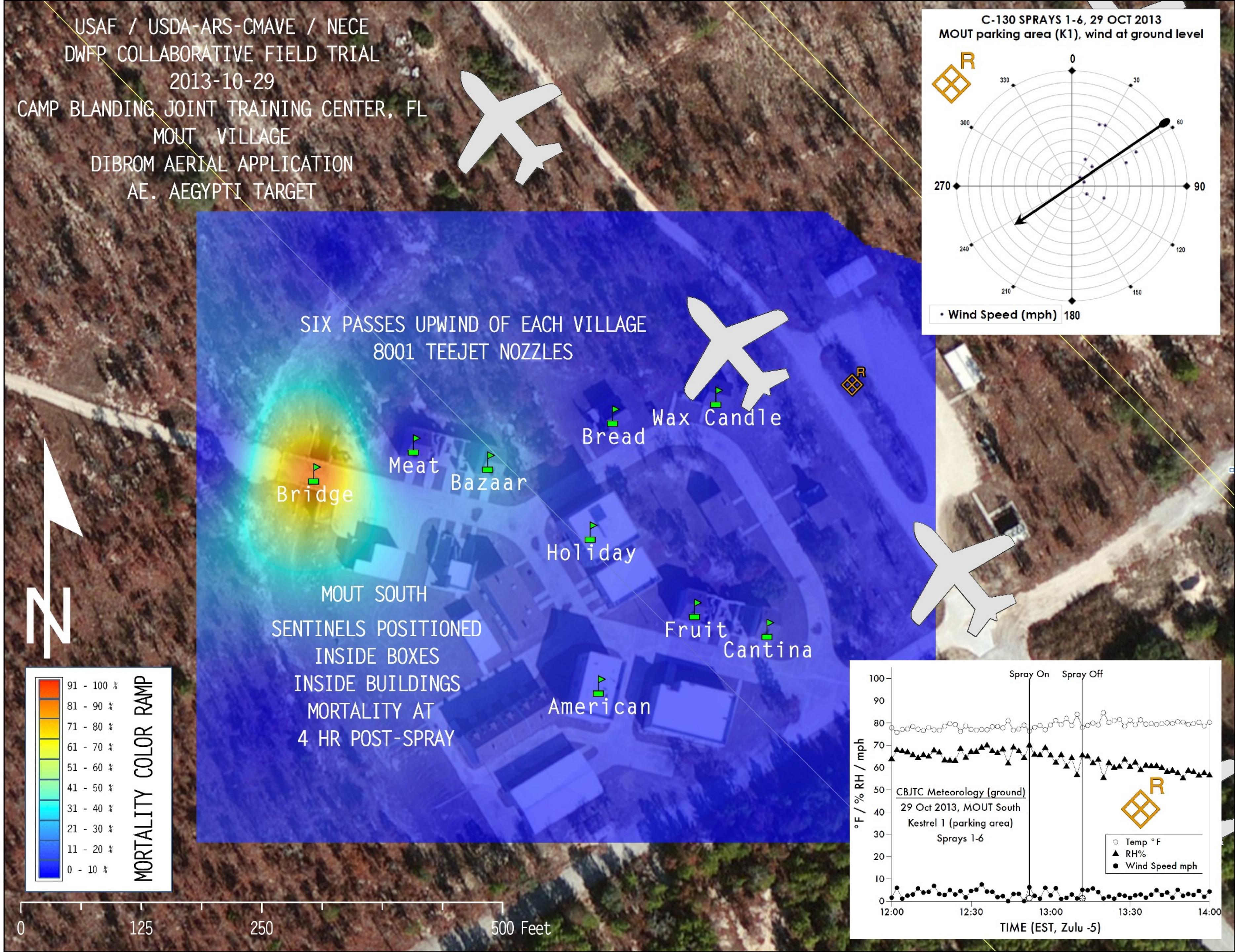

**Figure S9**
29 October 2013
8001 nozzles
MOUT South
12 hr mortality
Indoors **IN BOX**

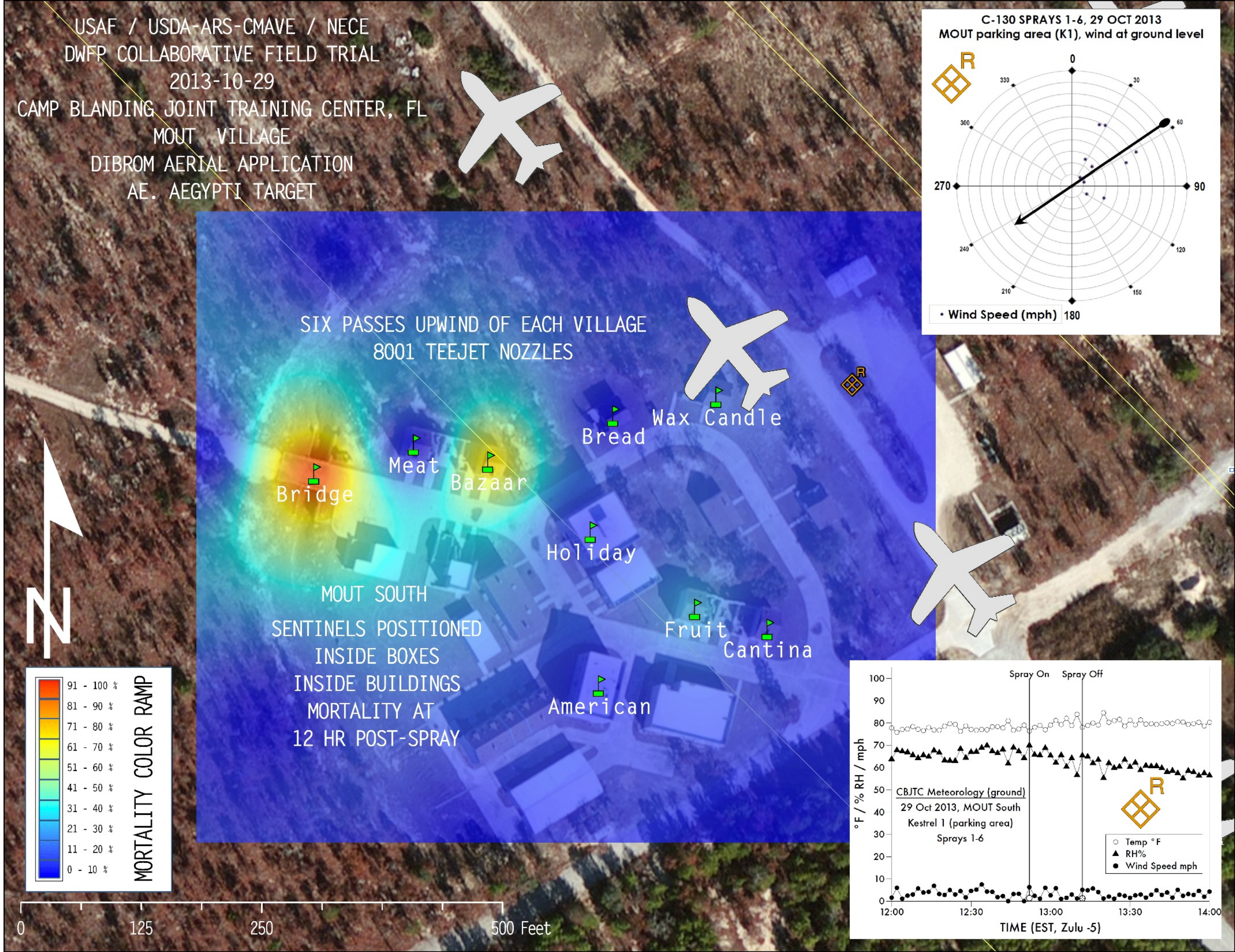

**Figure S10**
29 October 2013
8001 nozzles
MOUT South
1 hr mortality
Indoors **ON FLOOR**

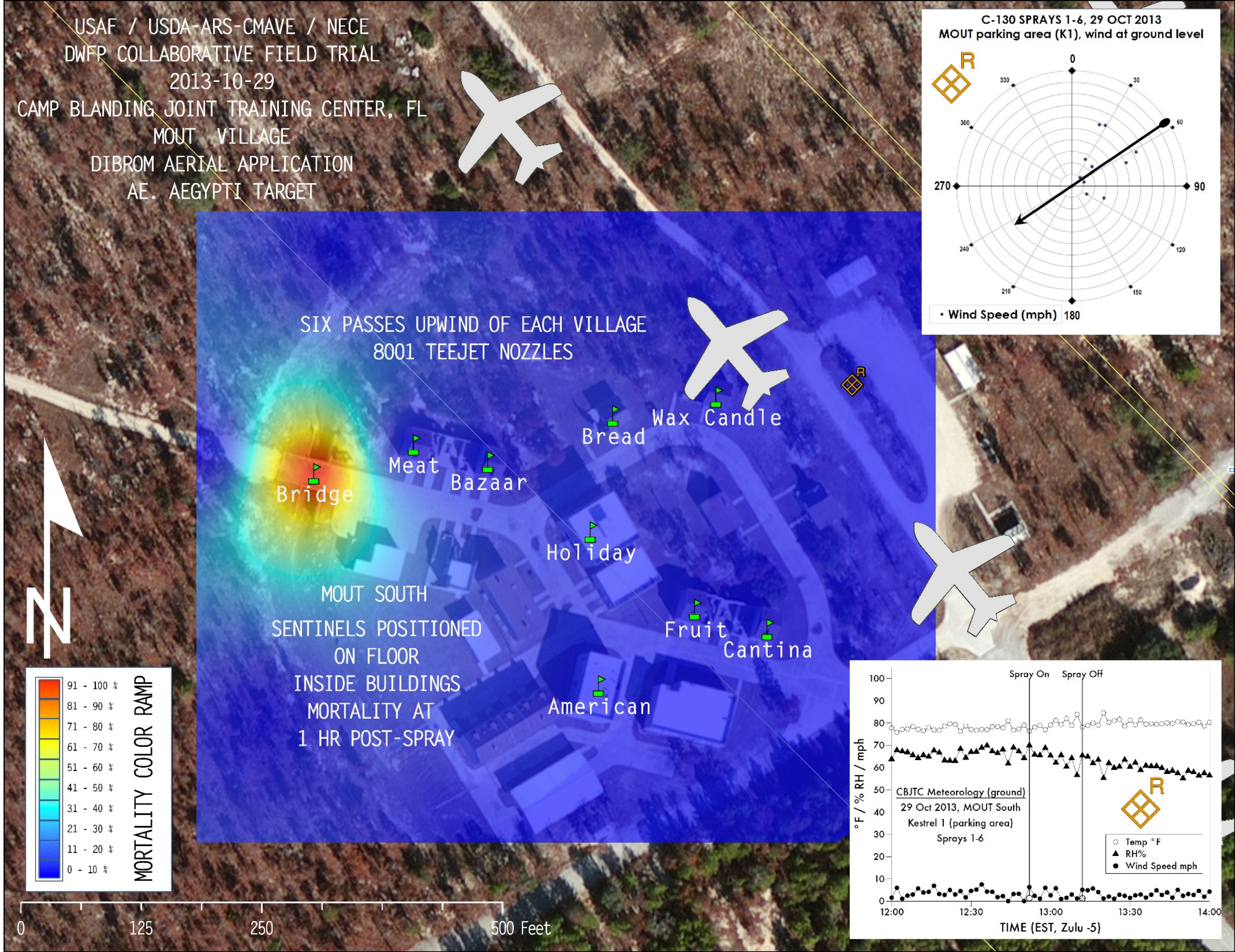

**Figure S11**

29 October 2013

8001 nozzles

MOUT South

4 hr mortality

Indoors **ON FLOOR**

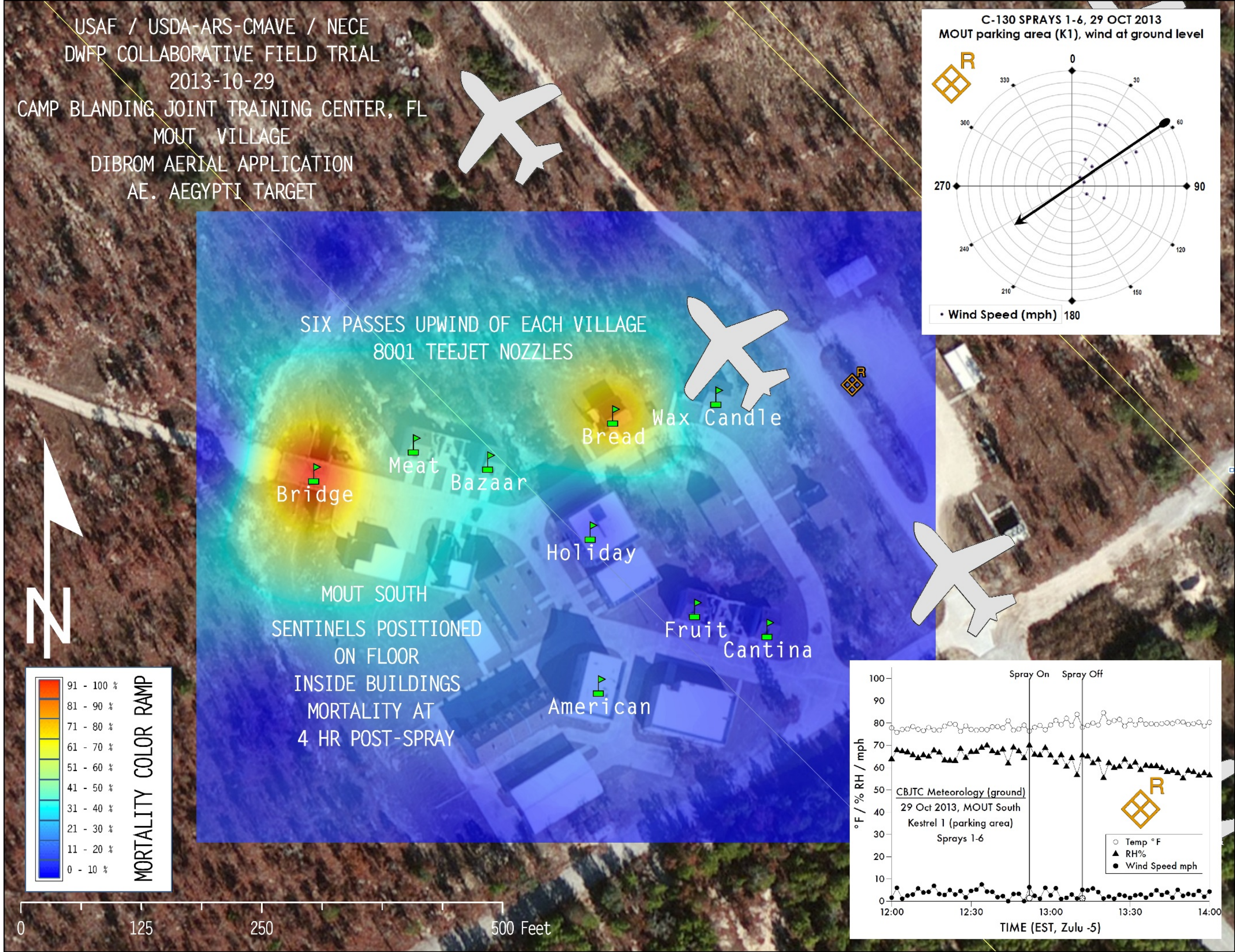

**Figure S12**
29 October 2013
8001 nozzles
MOUT South
12 hr mortality
Indoors **ON FLOOR**

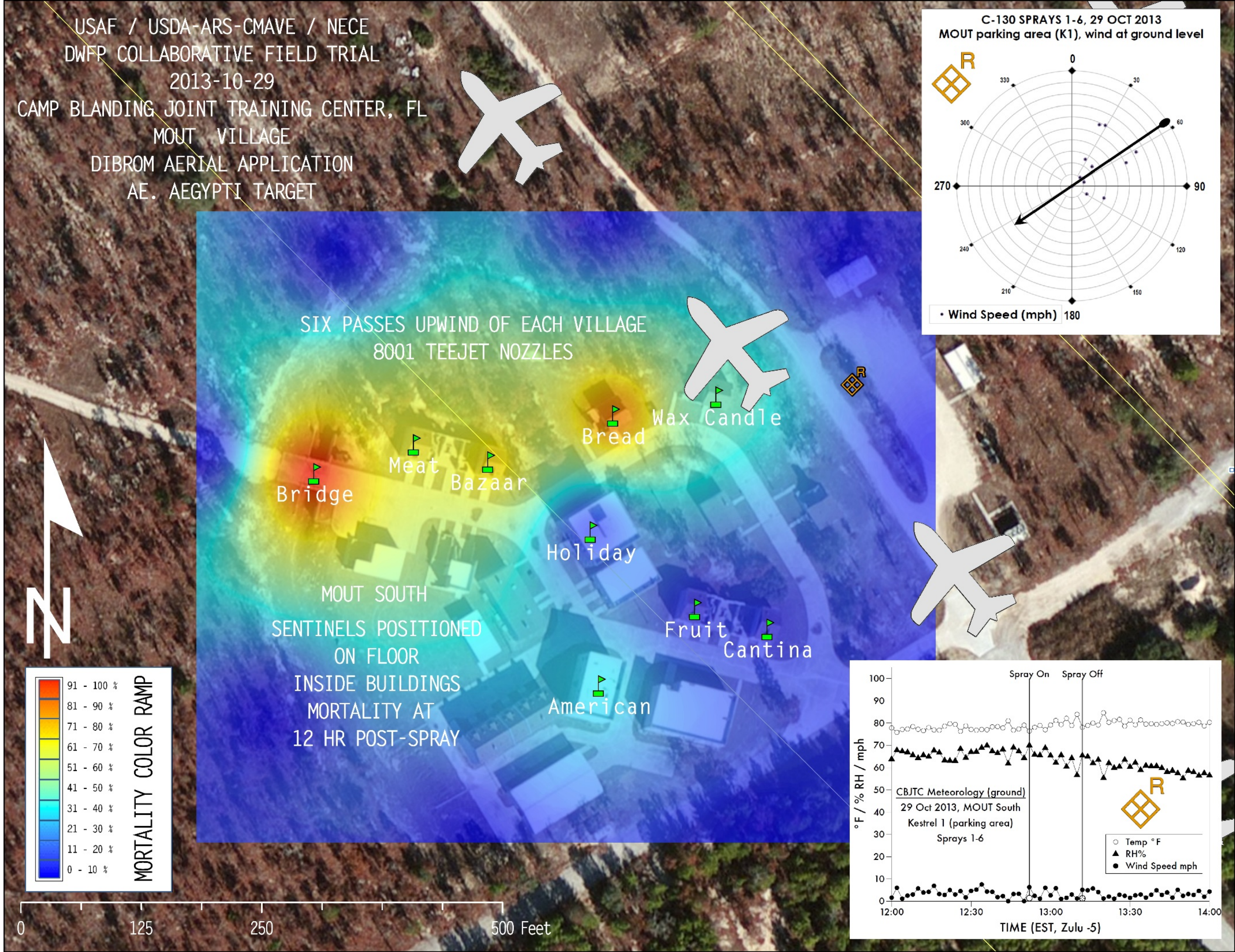

**SET B**

S13–S24 Figs. Naled efficacy (mortality), MOUT 30 Oct.

**Figure S13**
30 October 2013
8003 nozzles
MOUT South
1 hr mortality
Outdoors **IN BOX**

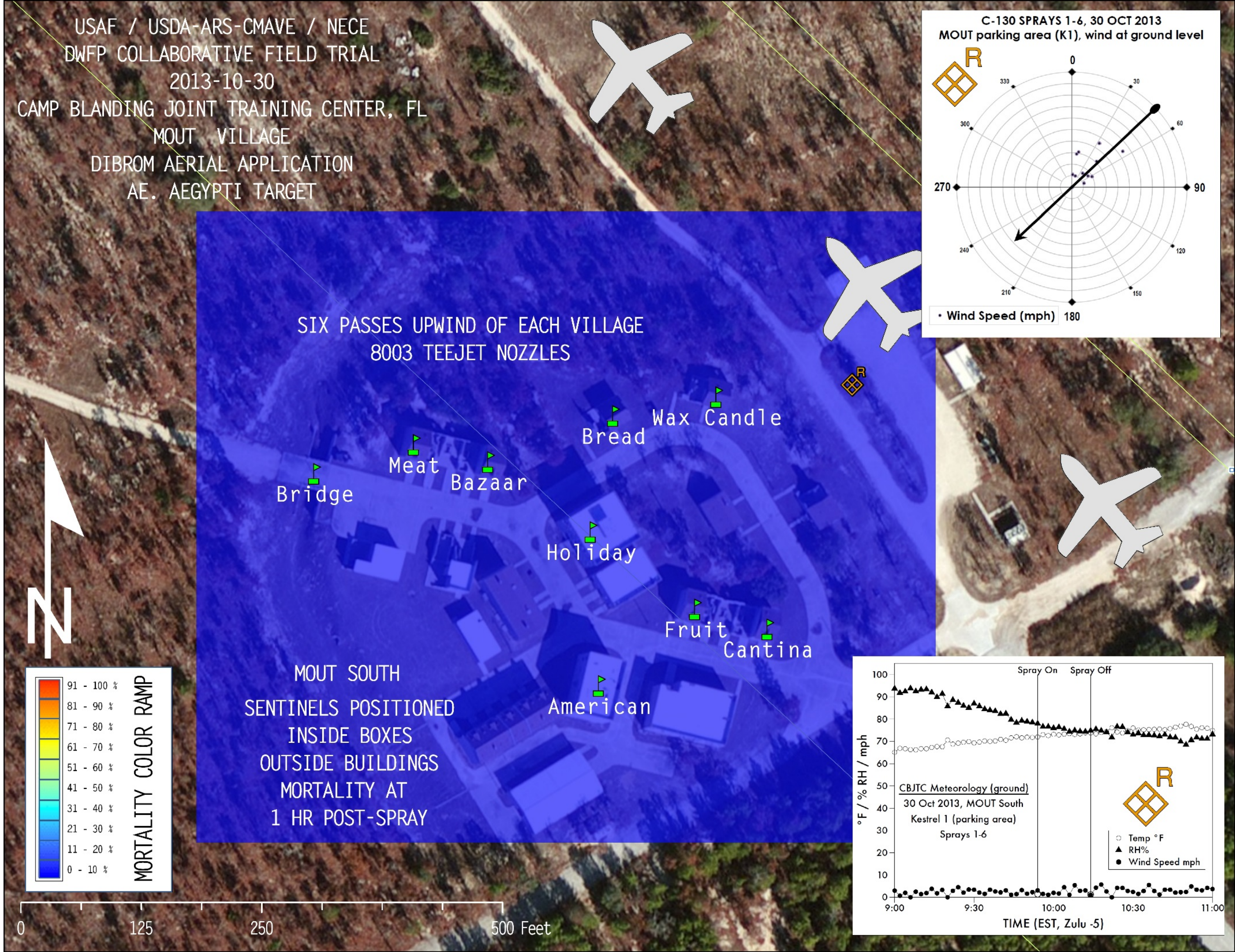

**Figure S14**
30 October 2013
8003 nozzles
MOUT South
4 hr mortality
Outdoors **IN BOX**

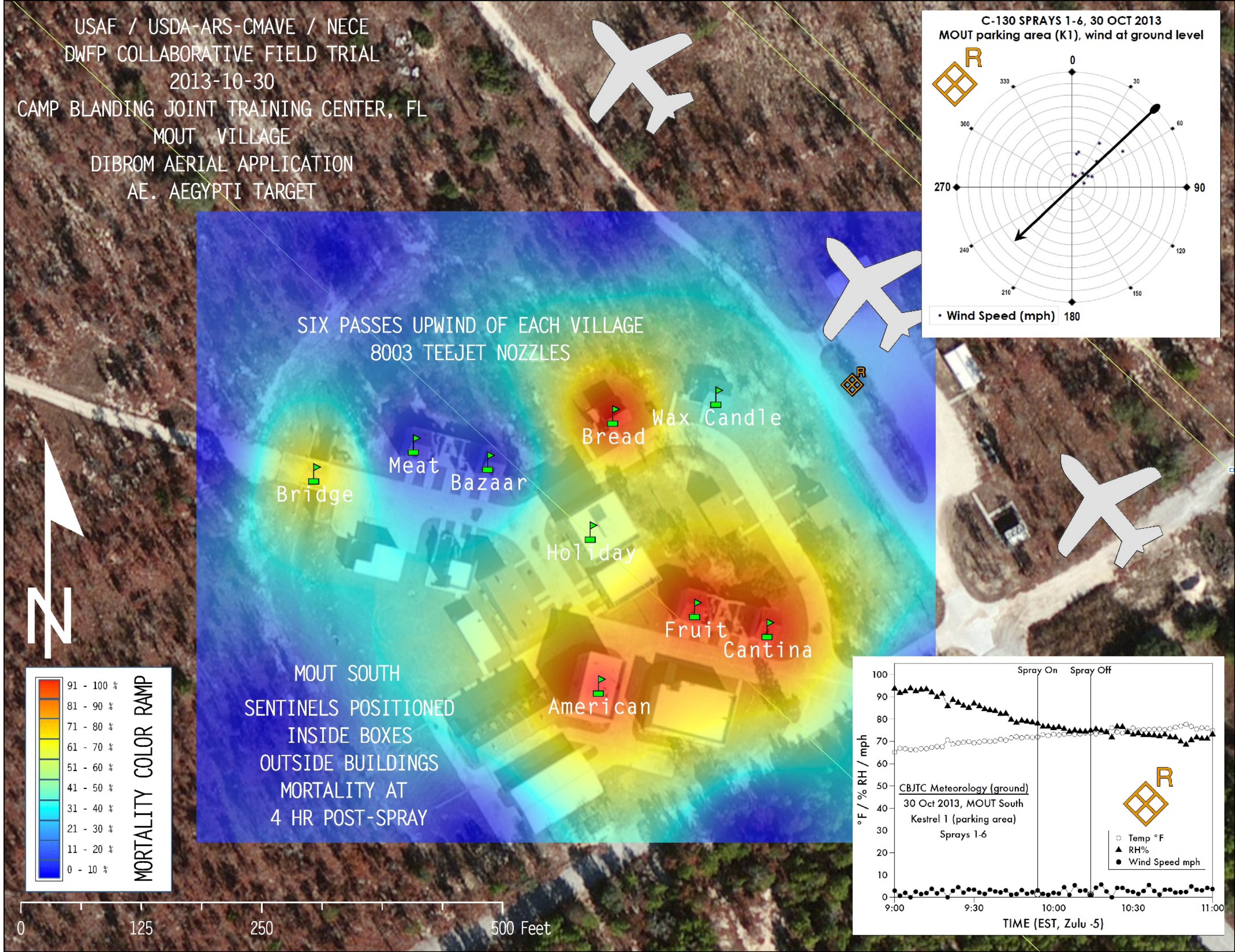

**Figure S15**
30 October 2013
8003 nozzles
MOUT South
12 hr mortality
Outdoors **IN BOX**

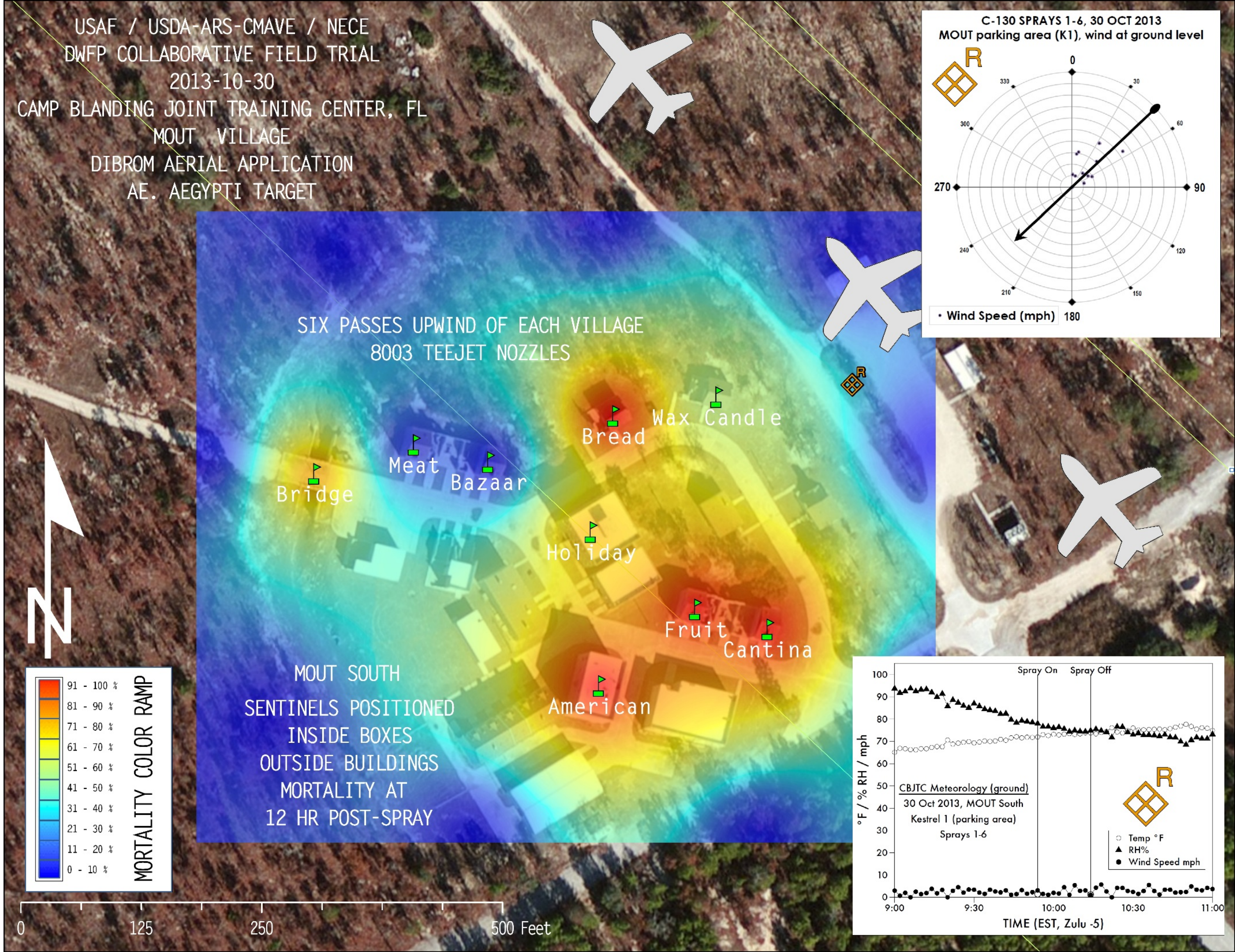

# Figure S16

30 October 2013

8003 nozzles

MOUT South

1 hr mortality

Outdoors **ON POLE**

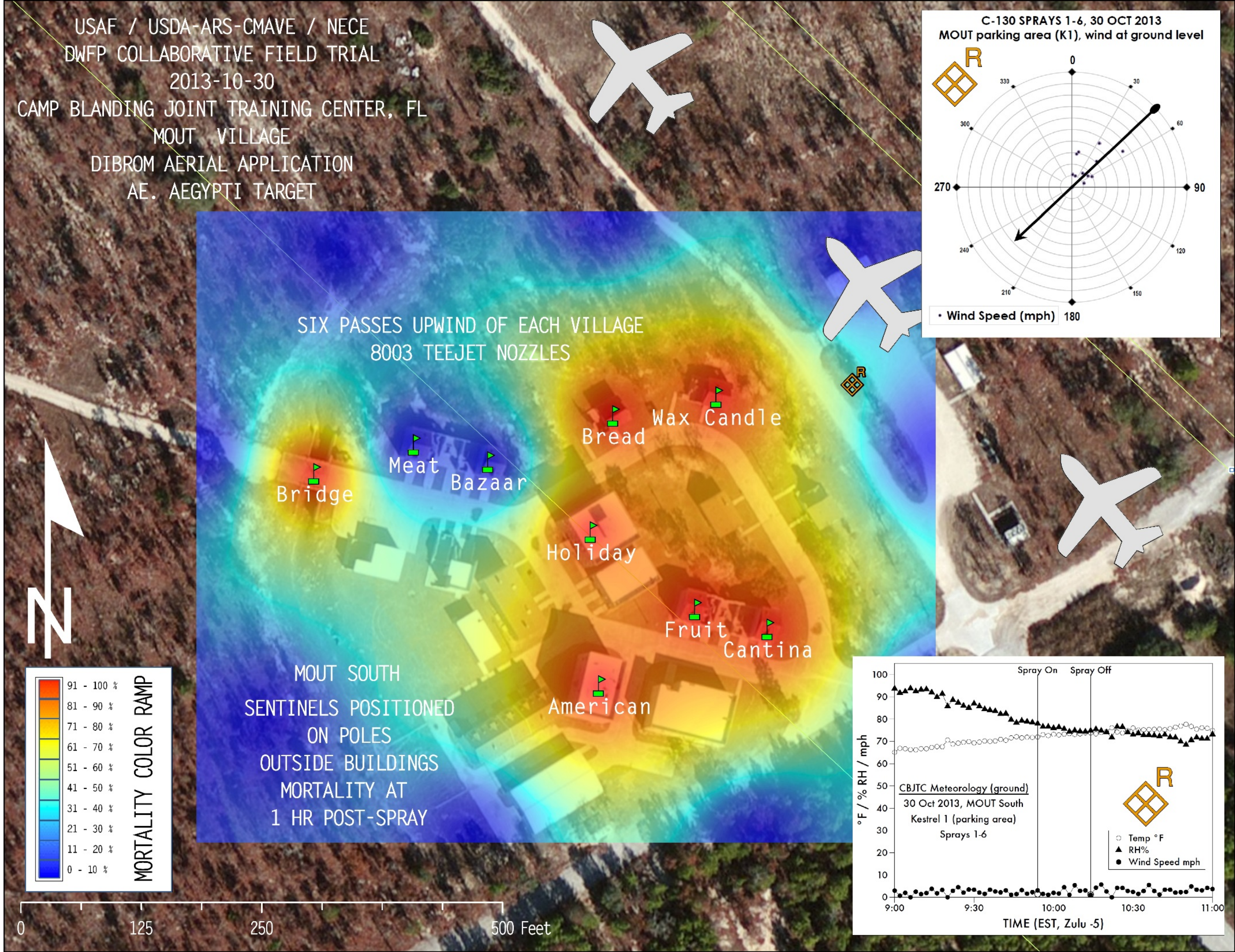

**Figure S17**
30 October 2013
8003 nozzles
MOUT South
4 hr mortality
Outdoors **ON POLE**

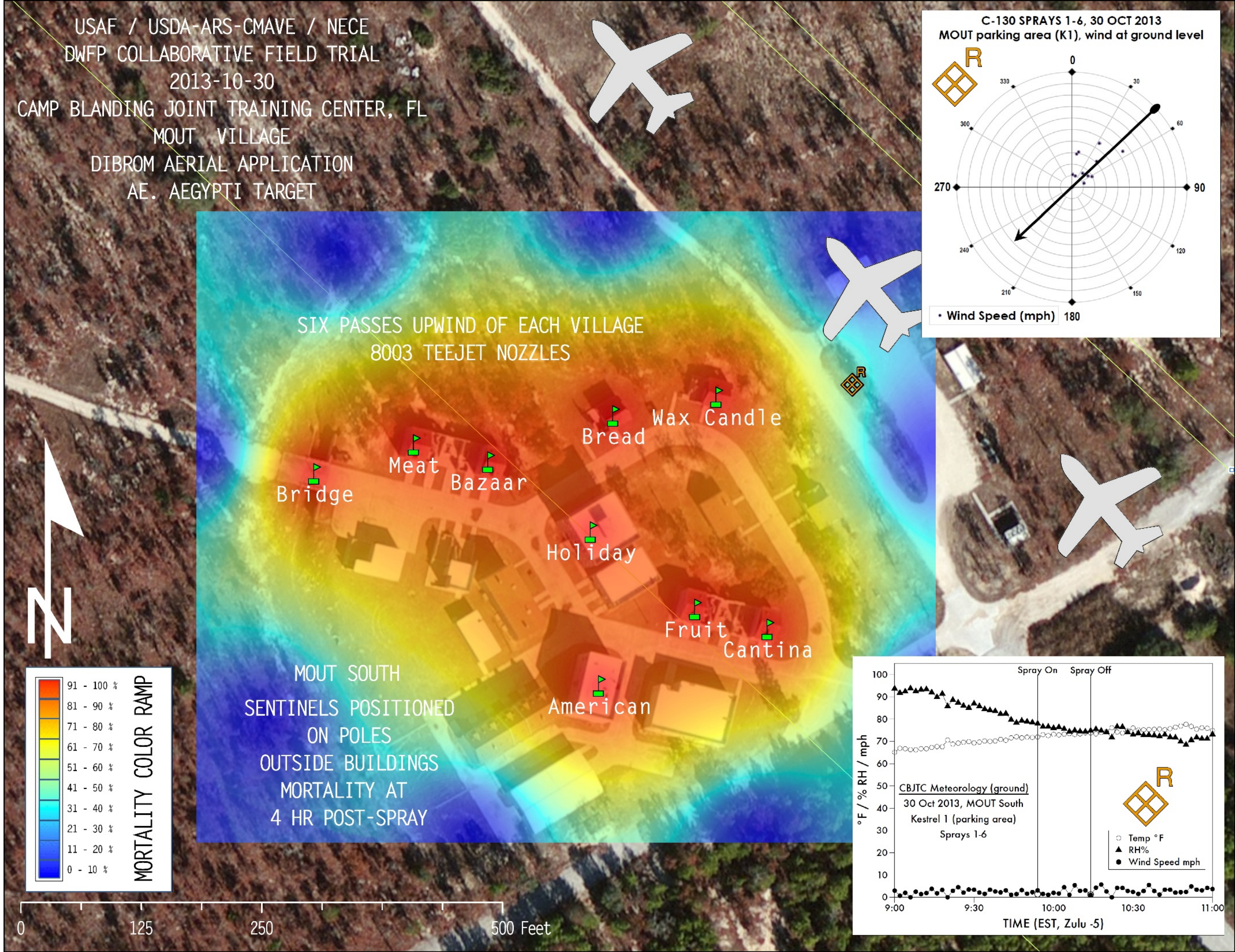

**Figure S18**

30 October 2013

8003 nozzles

MOUT South

12 hr mortality

Outdoors **ON POLE**

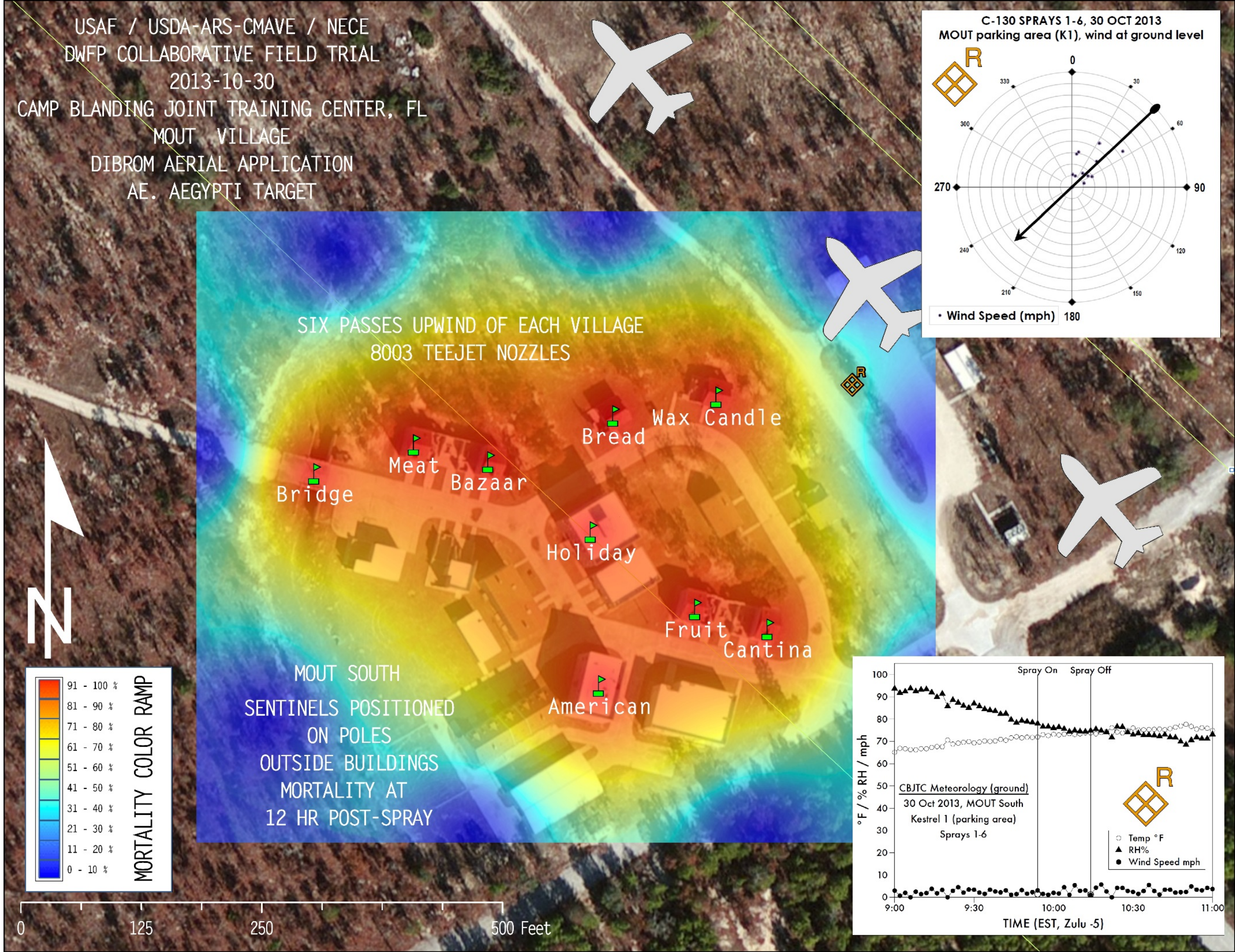

**Figure S19**
30 October 2013
8003 nozzles
MOUT South
1 hr mortality
Indoors **IN BOX**

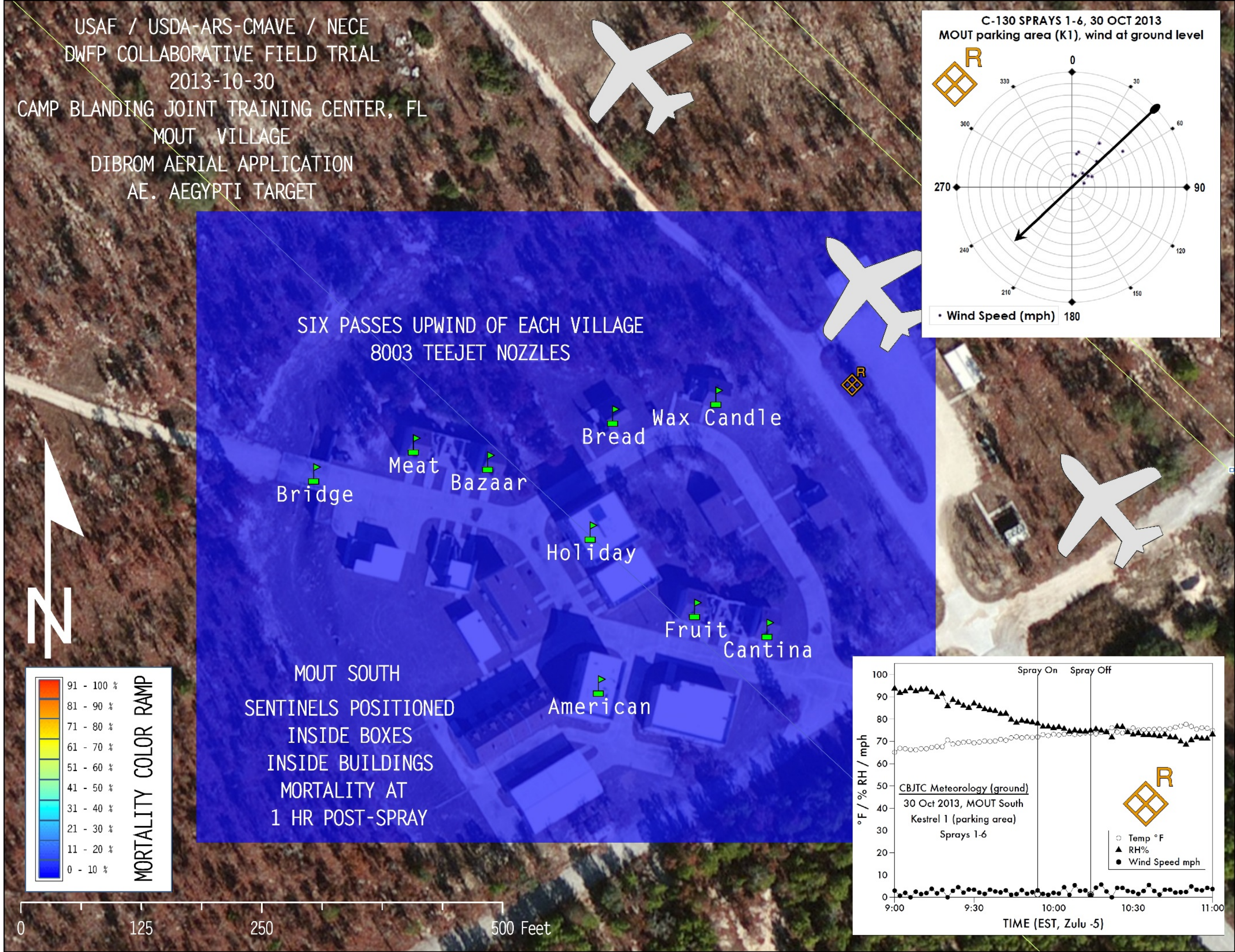

**Figure S20**
30 October 2013
8003 nozzles
MOUT South
4 hr mortality
Indoors **IN BOX**

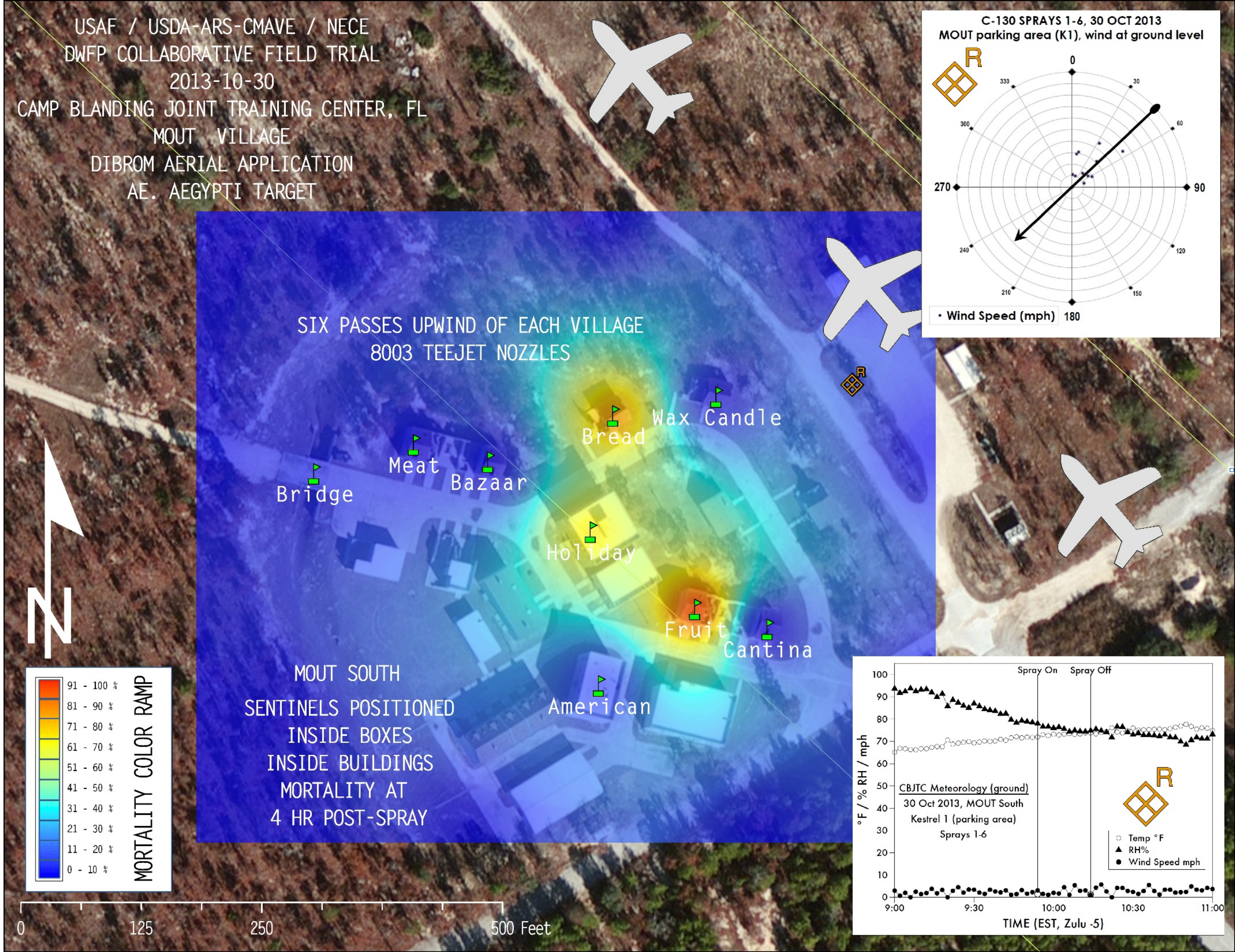

# Figure S21

30 October 2013
8003 nozzles
MOUT South
12 hr mortality
Indoors **IN BOX**

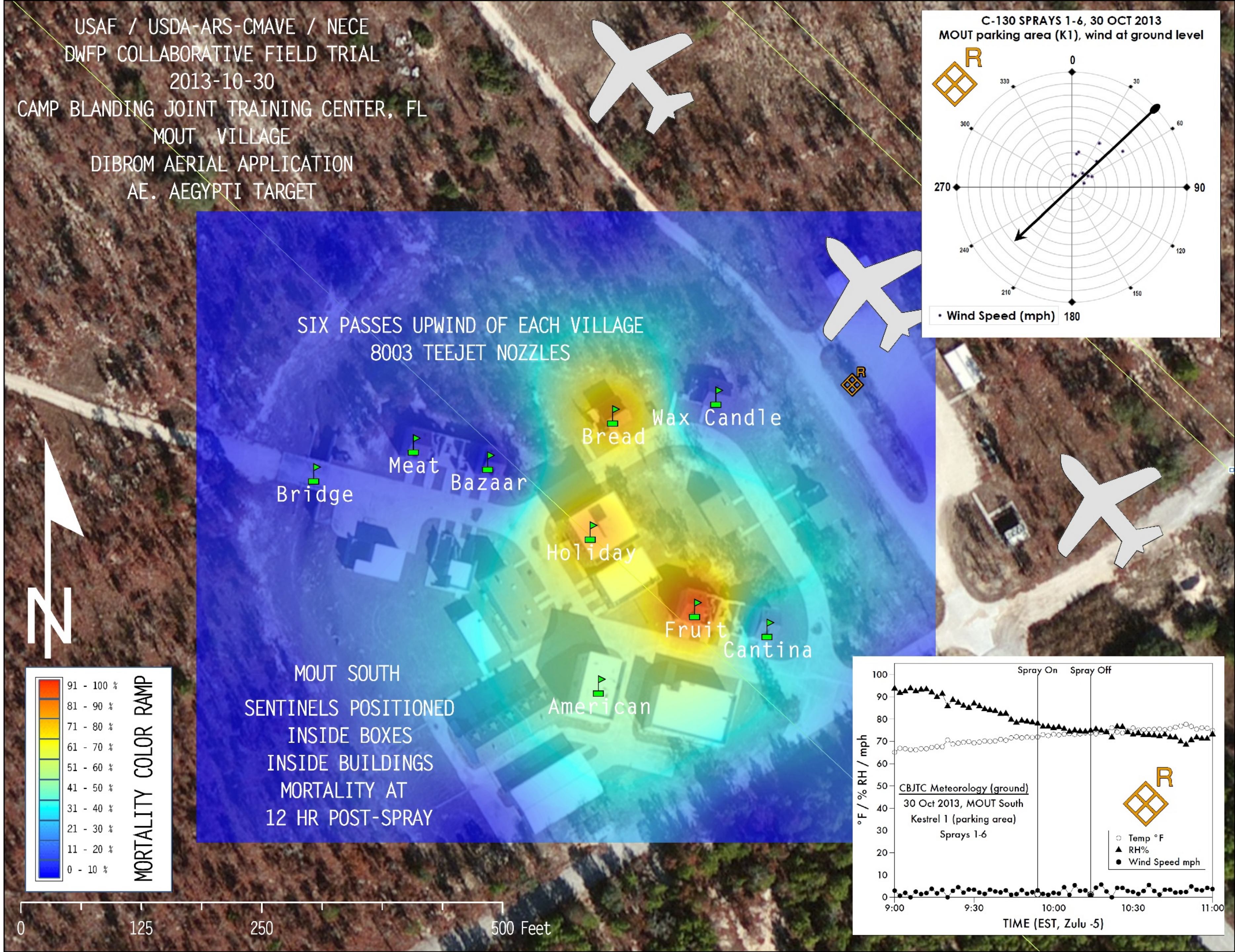

**Figure S22**
30 October 2013
8003 nozzles
MOUT South
1 hr mortality
Indoors **ON FLOOR**

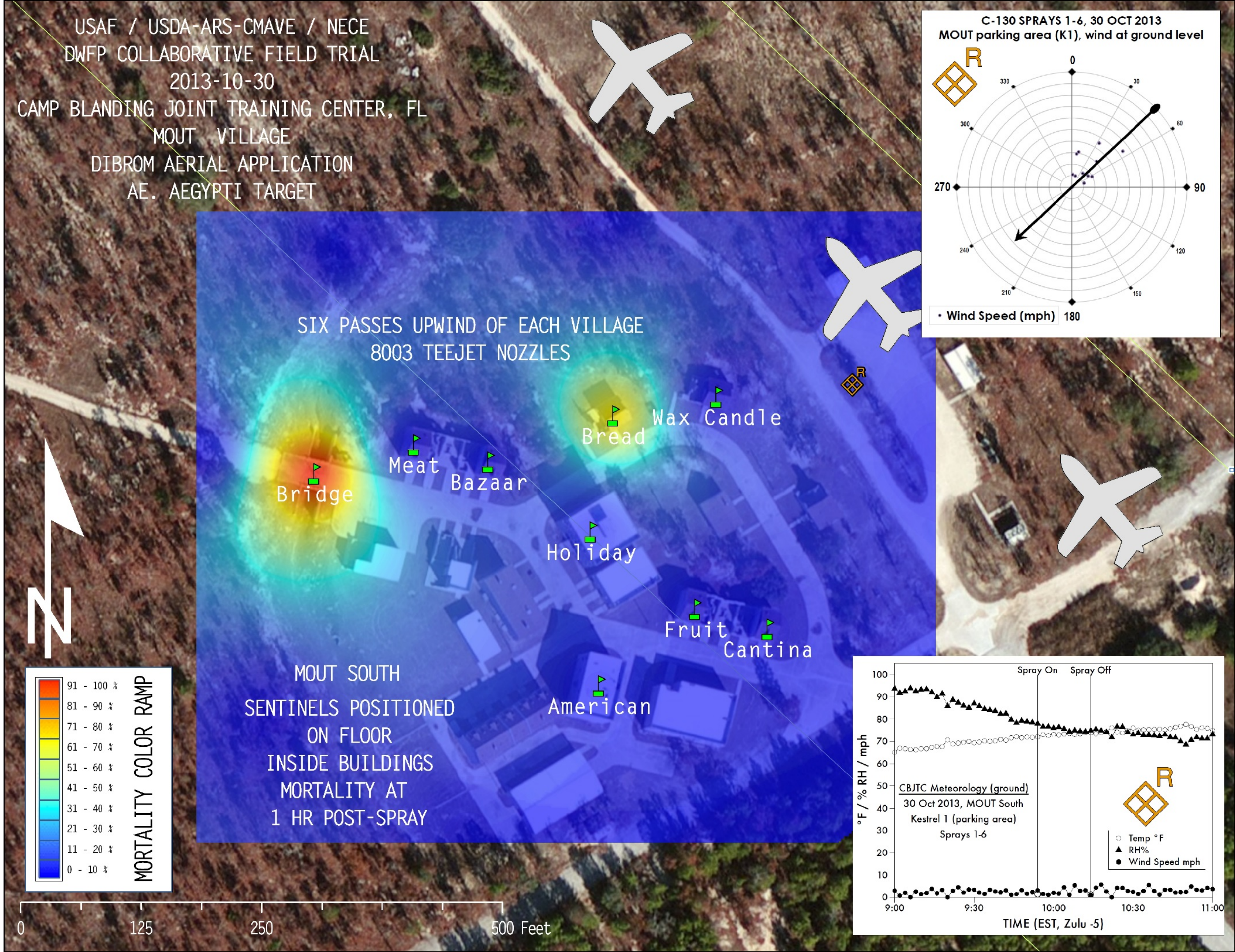

**Figure S23**
30 October 2013
8003 nozzles
MOUT South
4 hr mortality
Indoors **ON FLOOR**

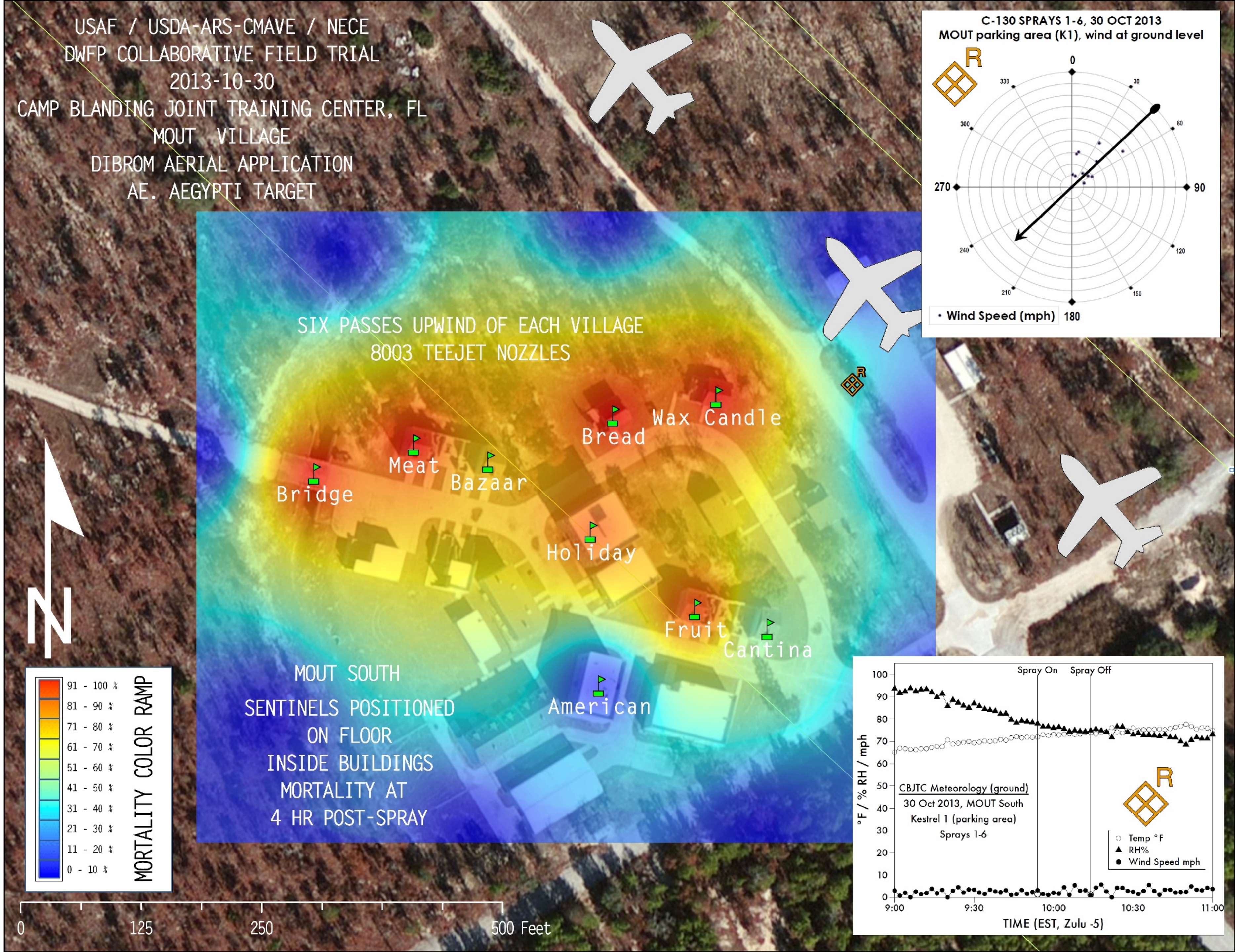

**Figure S24**

30 October 2013

8003 nozzles

MOUT South

12 hr mortality

Indoors **ON FLOOR**

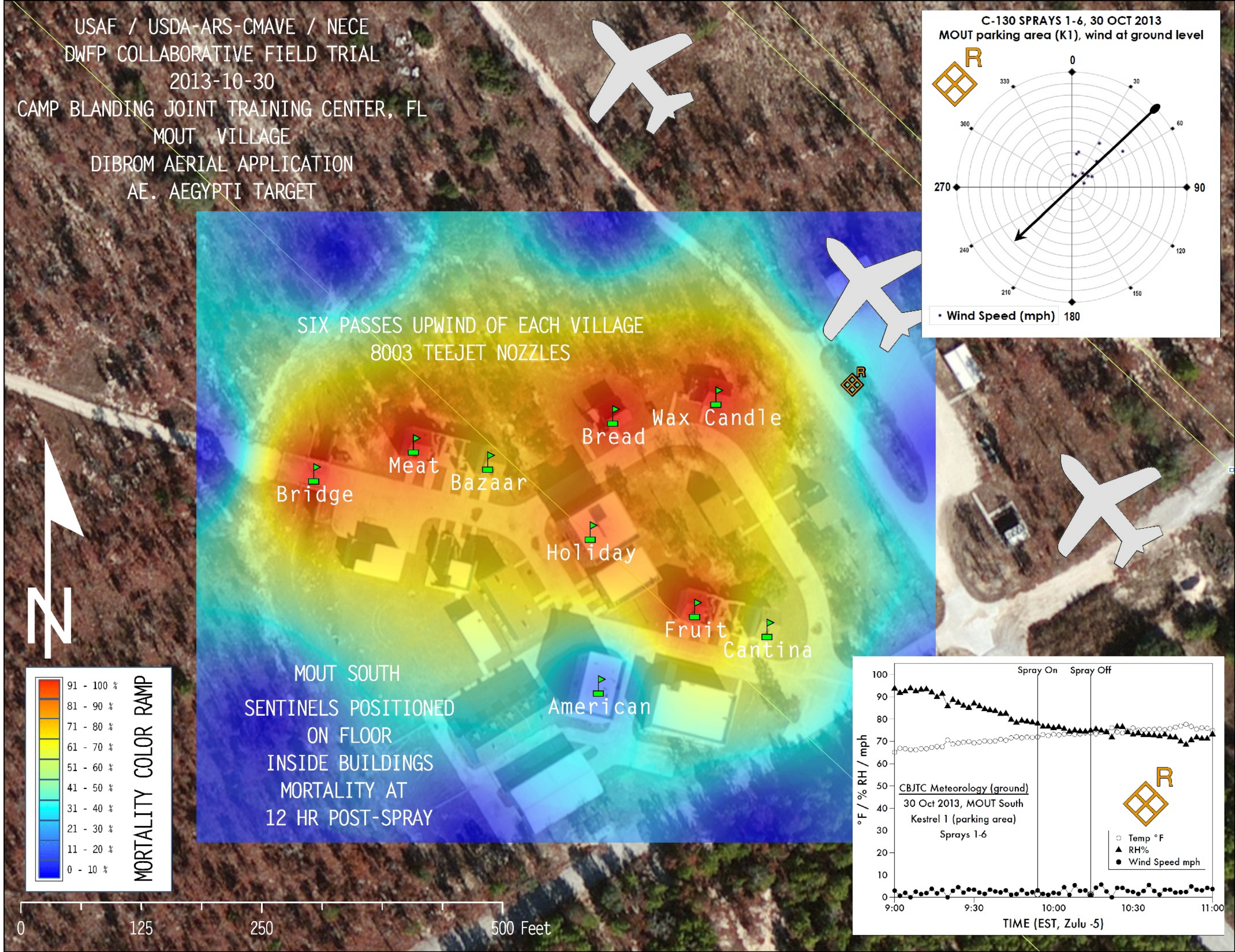

**SET C**

S25–S36 Figs. Naled efficacy (mortality), Village 29 Oct.

**Figure S25**
29 October 2013
8001 nozzles
Village
1 hr mortality
Outdoors **IN BOX**

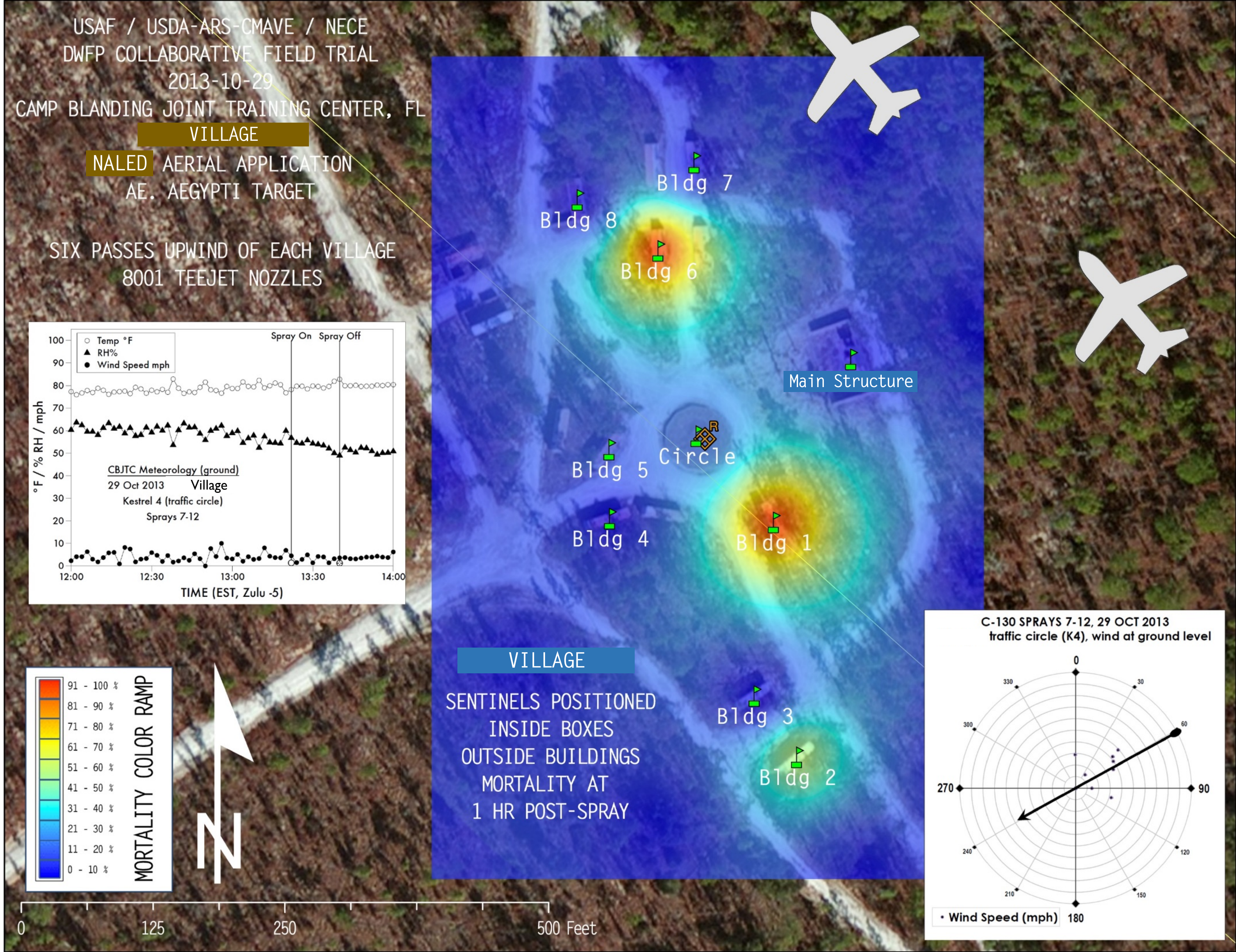

**Figure S26**
29 October 2013
8001 nozzles
Village
4 hr mortality
Outdoors **IN BOX**

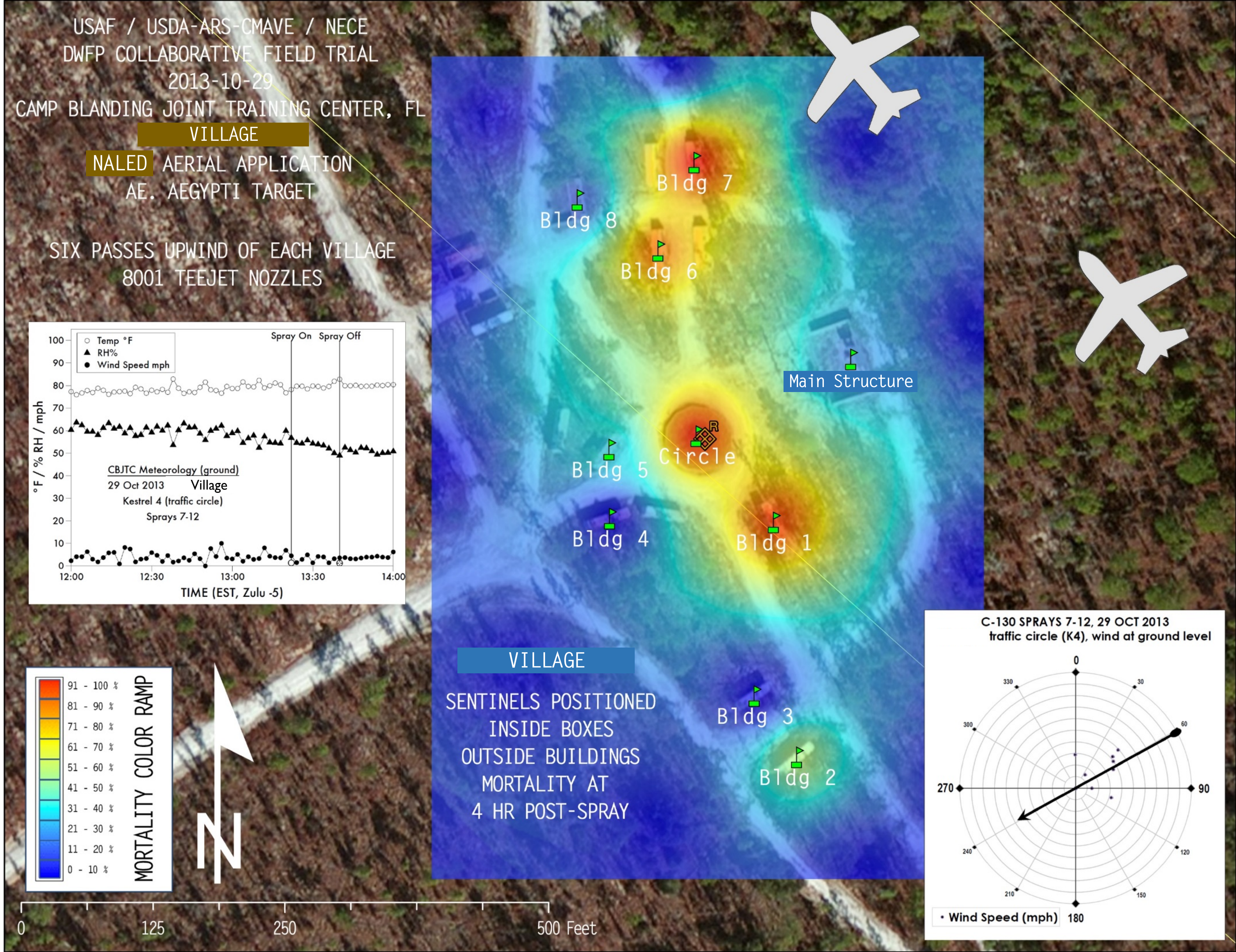

**Figure S27**
29 October 2013
8001 nozzles
Village
12 hr mortality
Outdoors **IN BOX**

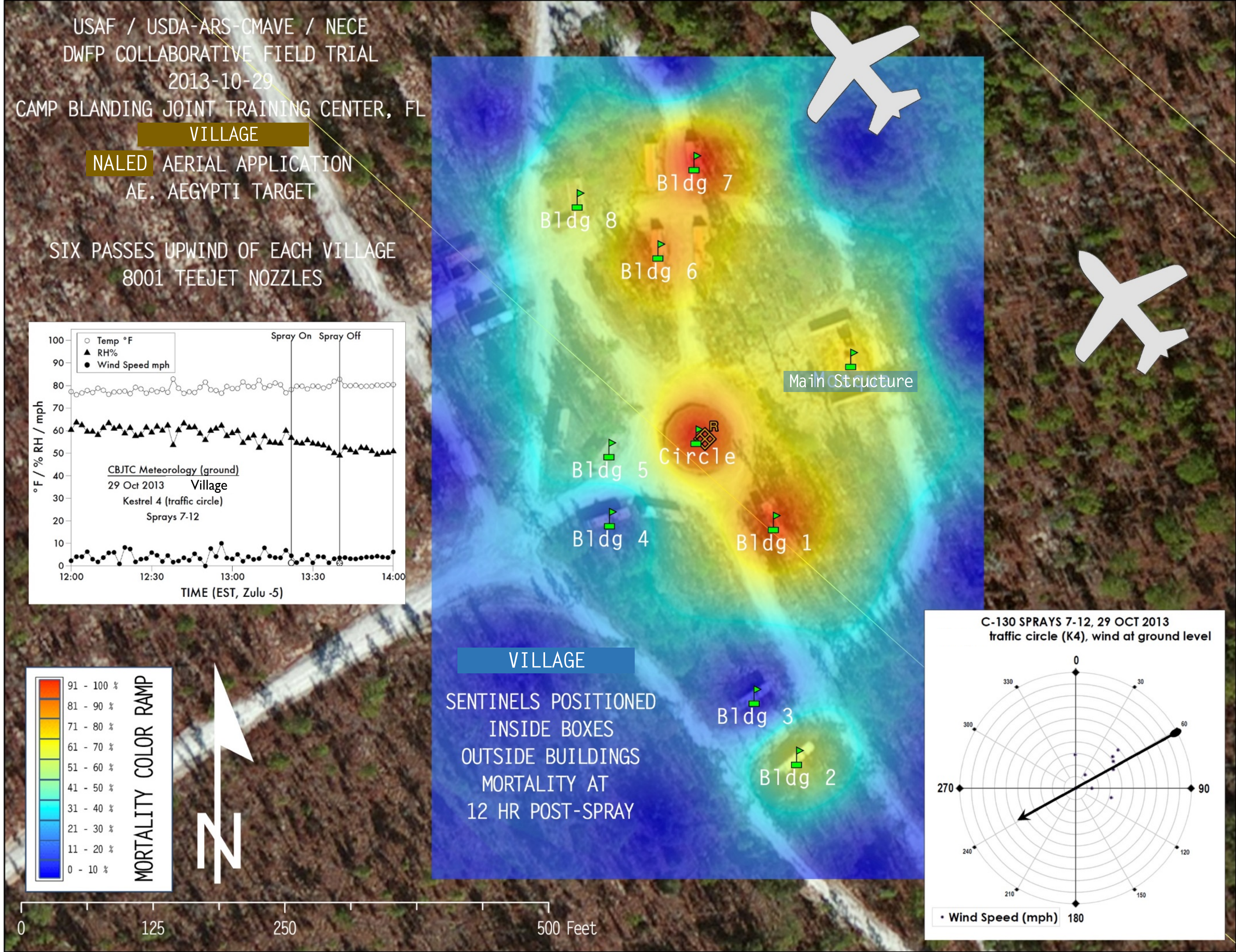

**Figure S28**
29 October 2013
8001 nozzles
Village
1 hr mortality
Outdoors **ON POLE**

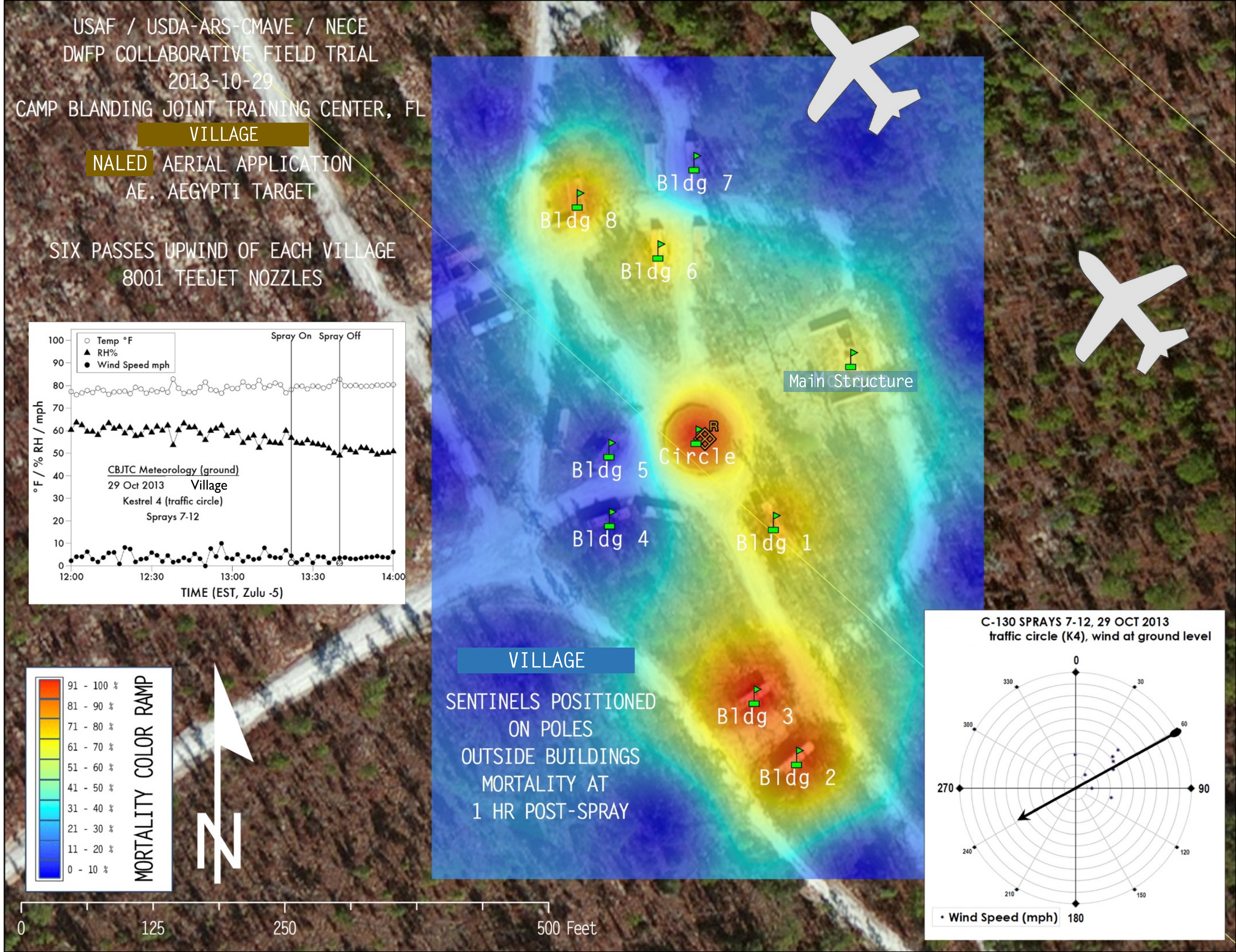

**Figure S29**
29 October 2013
8001 nozzles
Village
4 hr mortality
Outdoors **ON POLE**

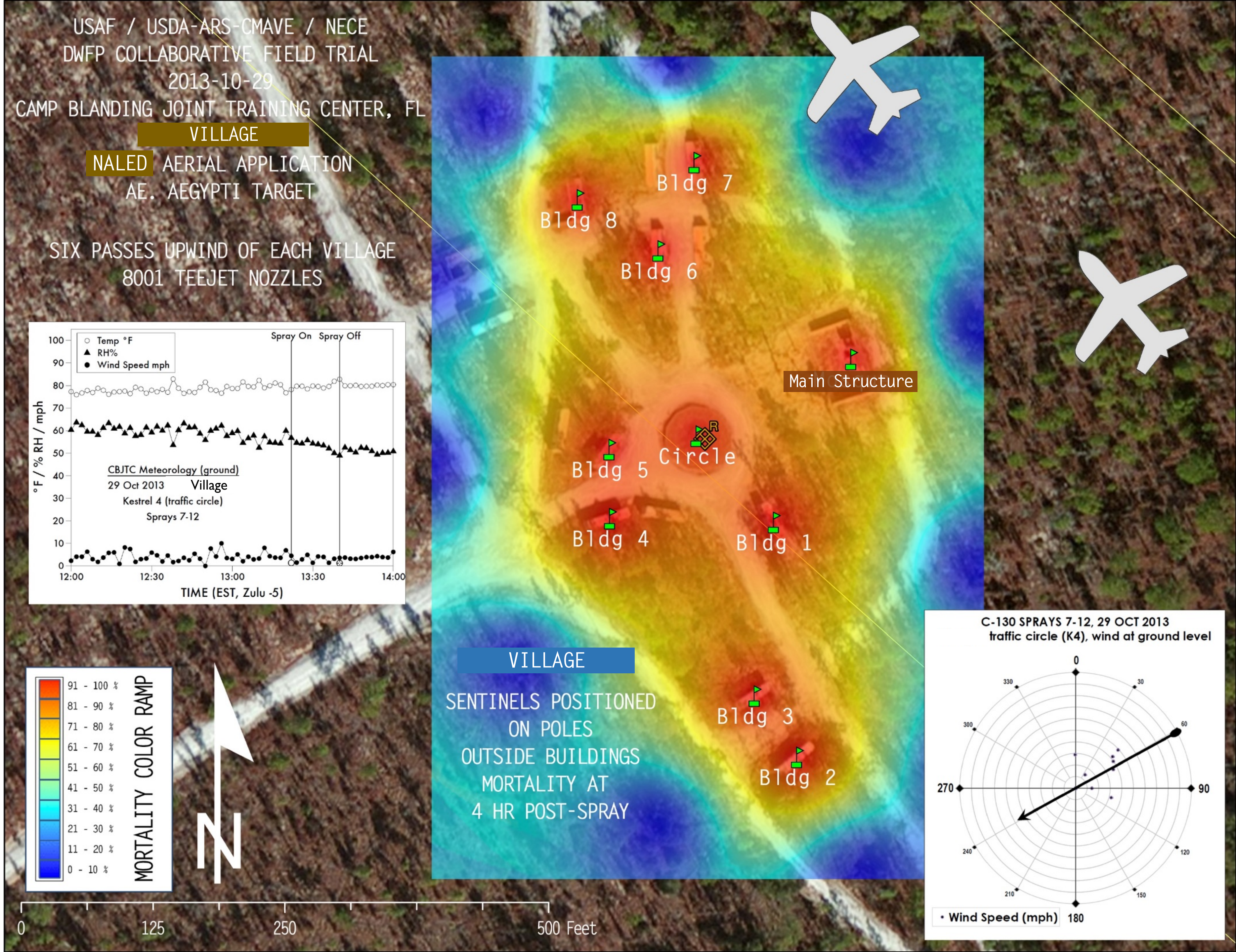

**Figure S30**
29 October 2013
8001 nozzles
Village
12 hr mortality
Outdoors **ON POLE**

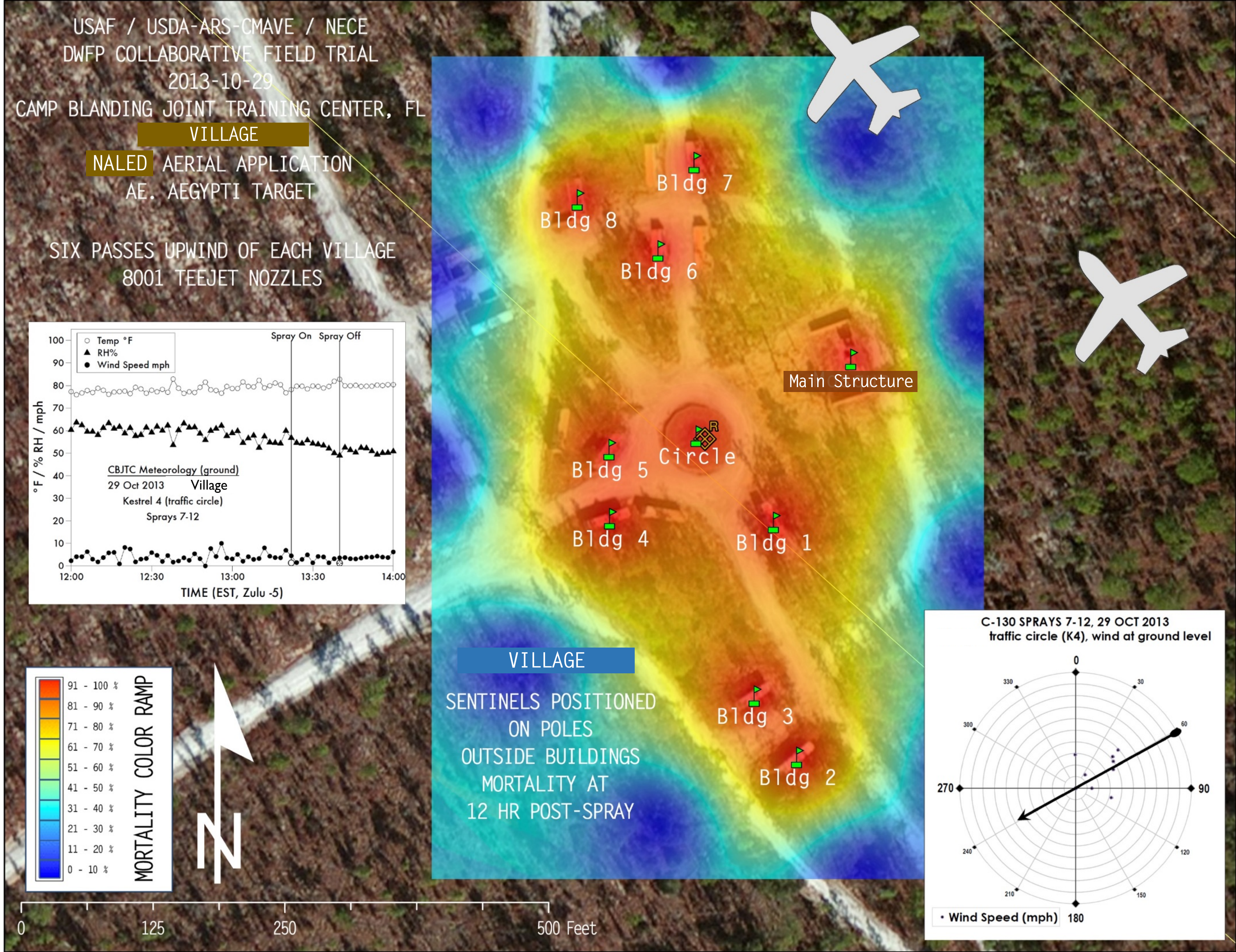

**Figure S31**
29 October 2013
8001 nozzles
Village
1 hr mortality
Indoors **IN BOX**

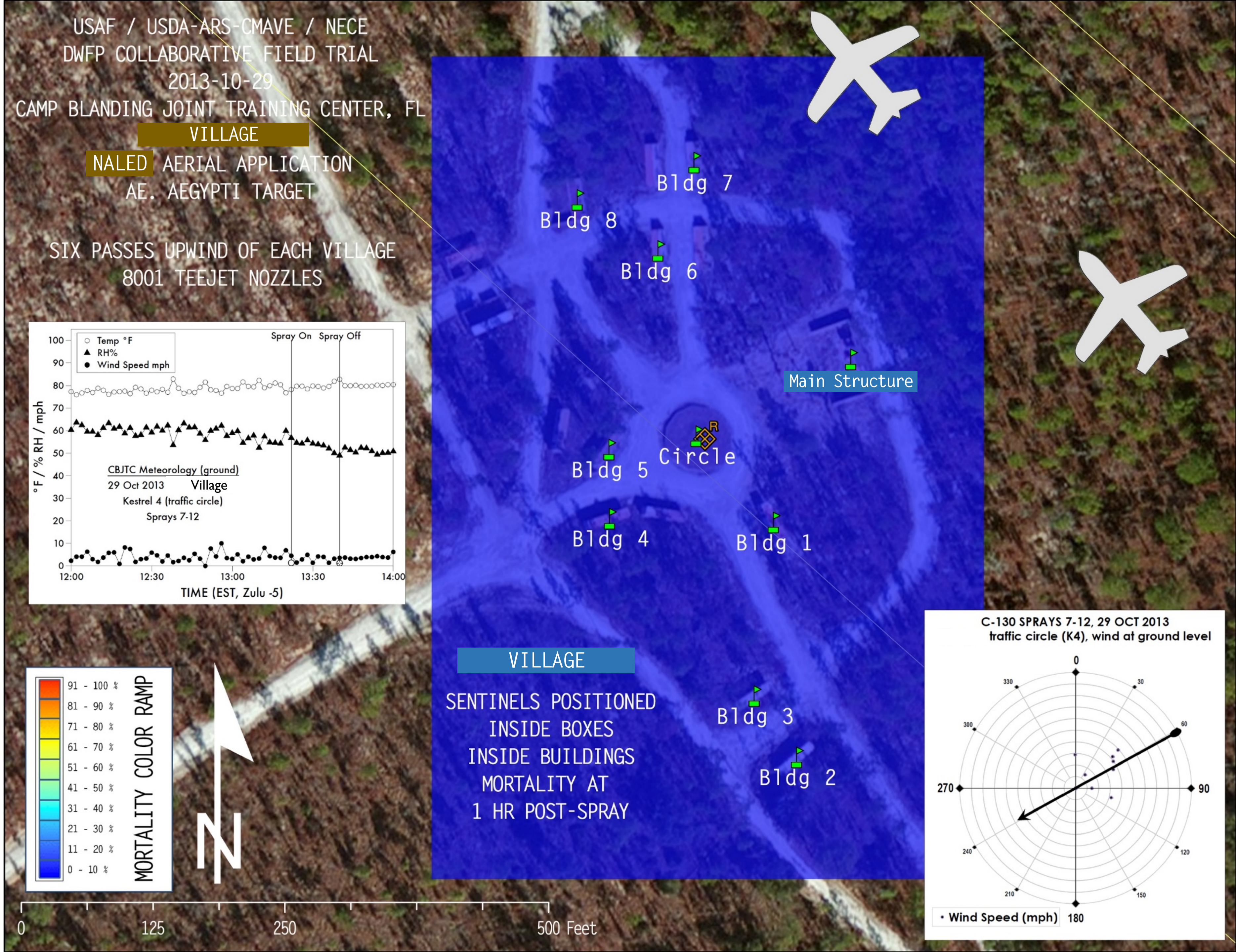

**Figure S32**
29 October 2013
8001 nozzles
Village
4 hr mortality
Indoors **IN BOX**

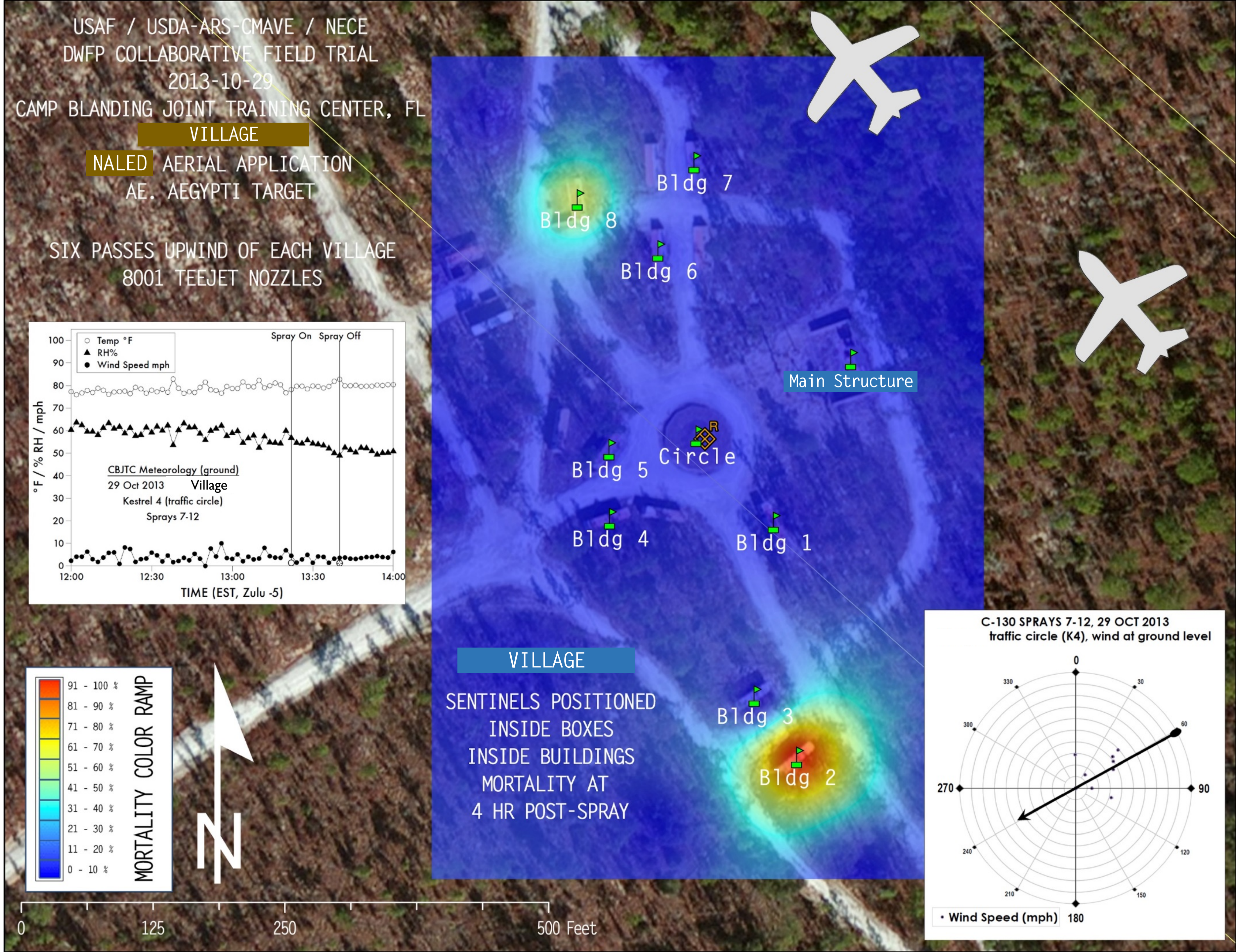

**Figure S33**
29 October 2013
8001 nozzles
Village
12 hr mortality
Indoors **IN BOX**

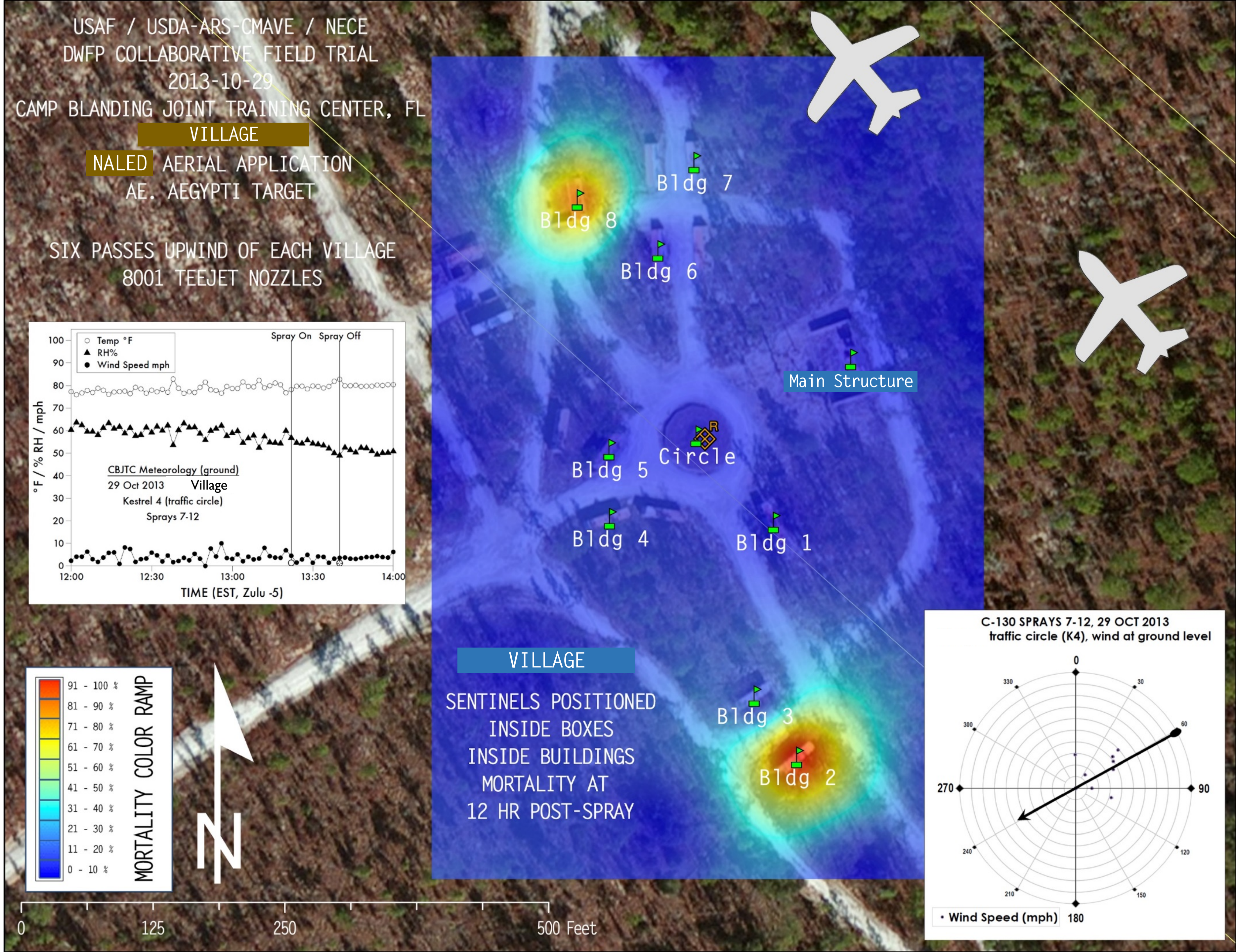

**Figure S34**
29 October 2013
8001 nozzles
Village
1 hr mortality
Indoors **ON FLOOR**

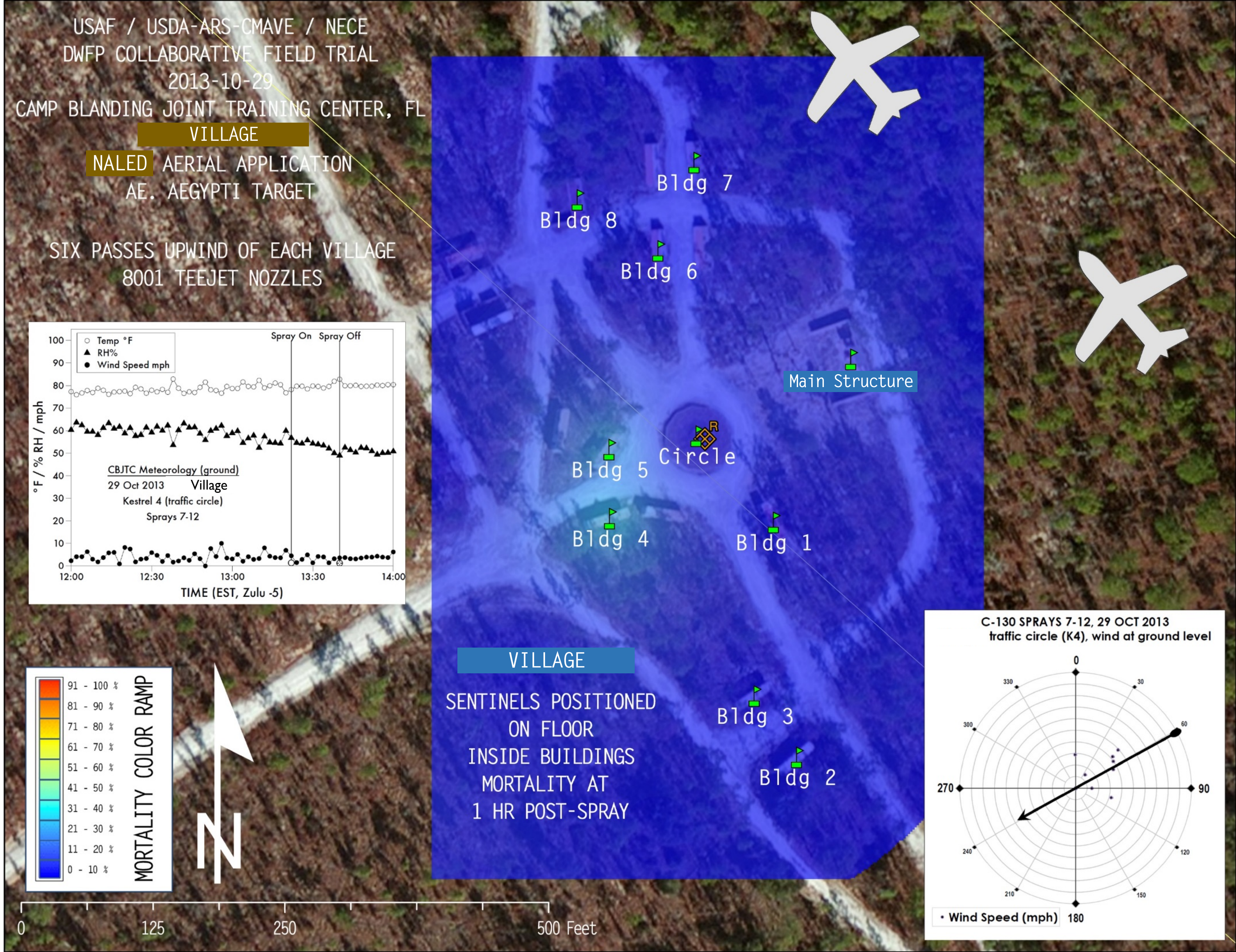

**Figure S35**
29 October 2013
8001 nozzles
Village
4 hr mortality
Indoors **ON FLOOR**

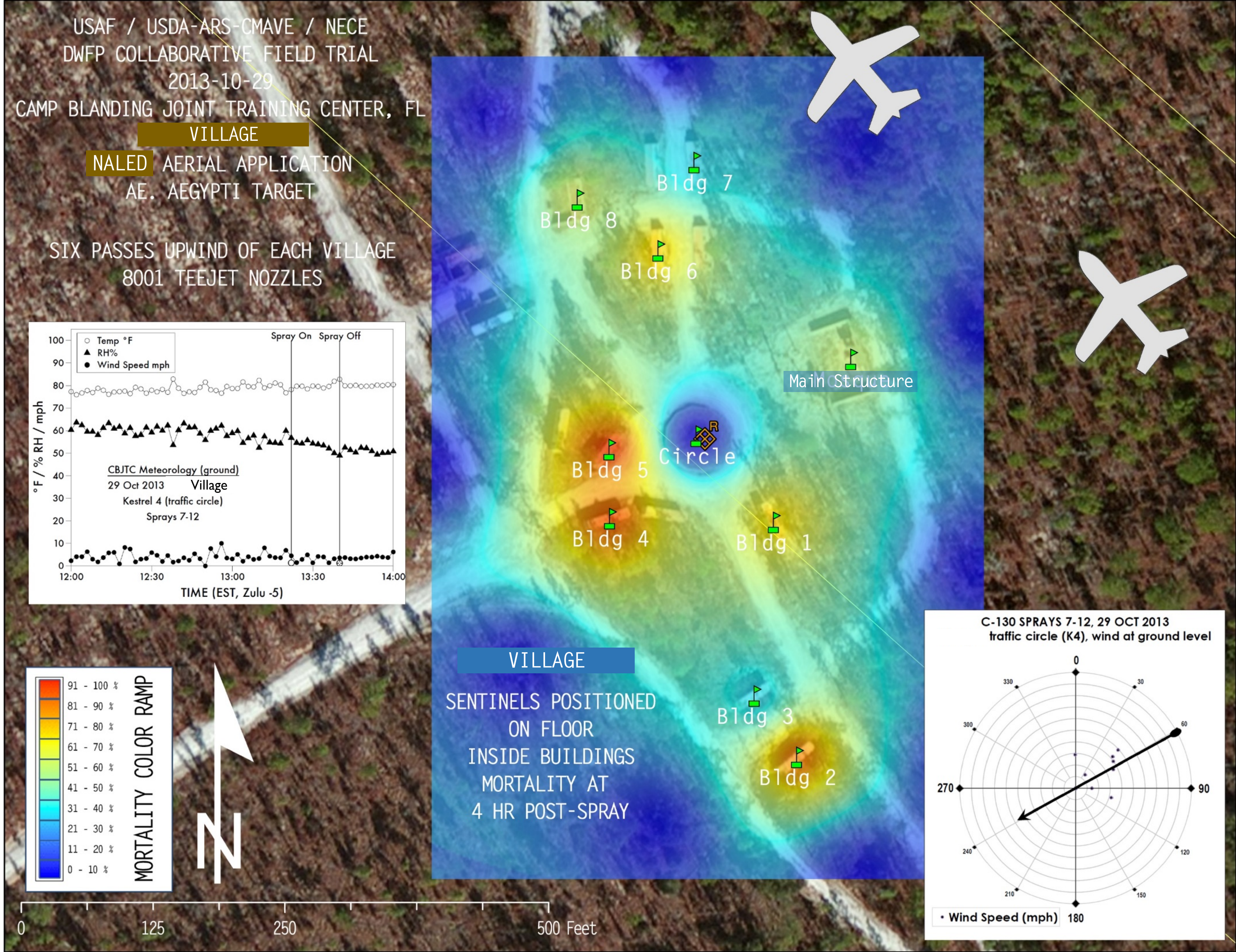

**Figure S36**
29 October 2013
8001 nozzles
Village
12 hr mortality
Indoors **ON FLOOR**

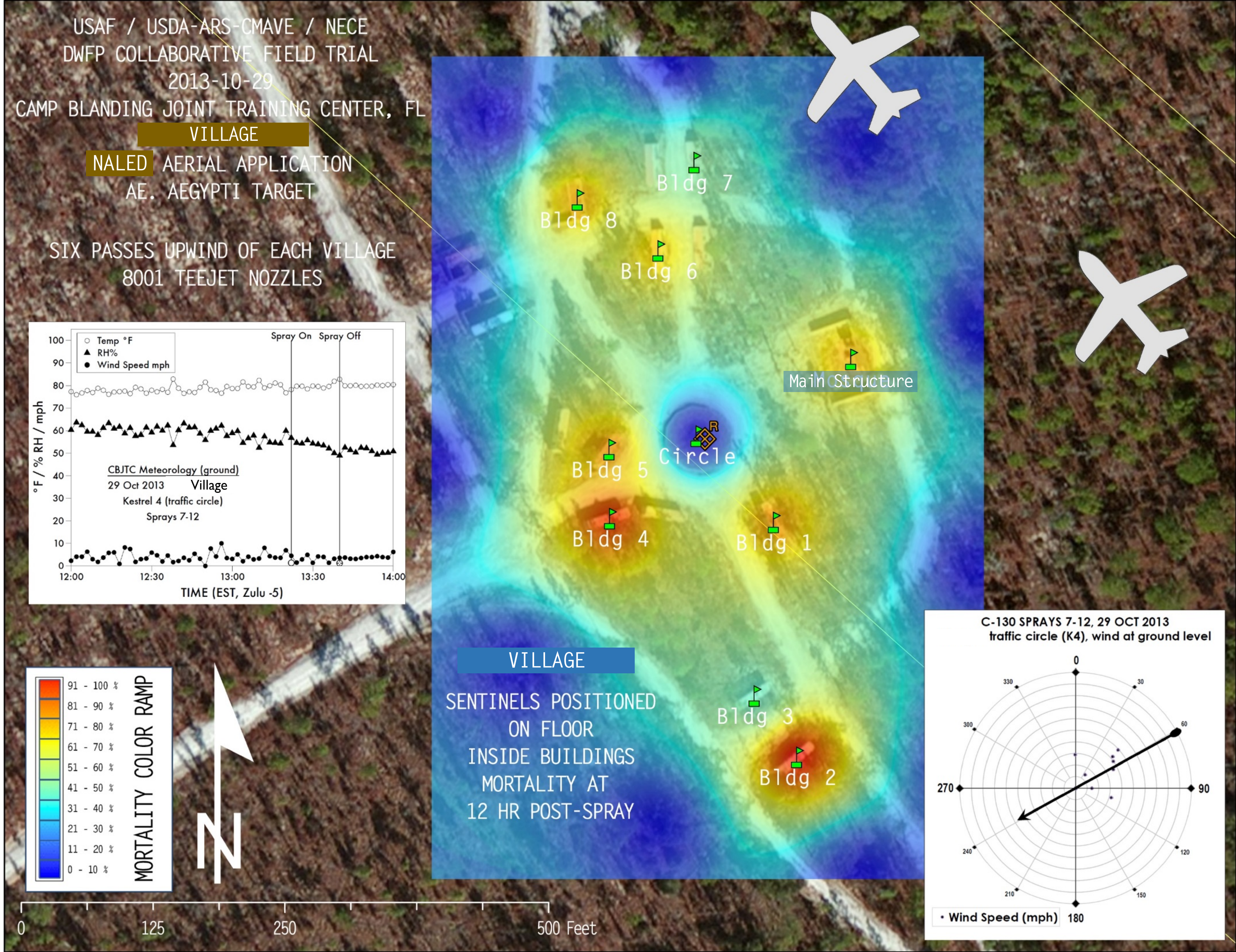

**SET D**

S37–S48 Figs. Naled efficacy (mortality), Village 30 Oct.

# Figure S37

30 October 2013

8003 nozzles

Village

1 hr mortality

Outdoors **IN BOX**

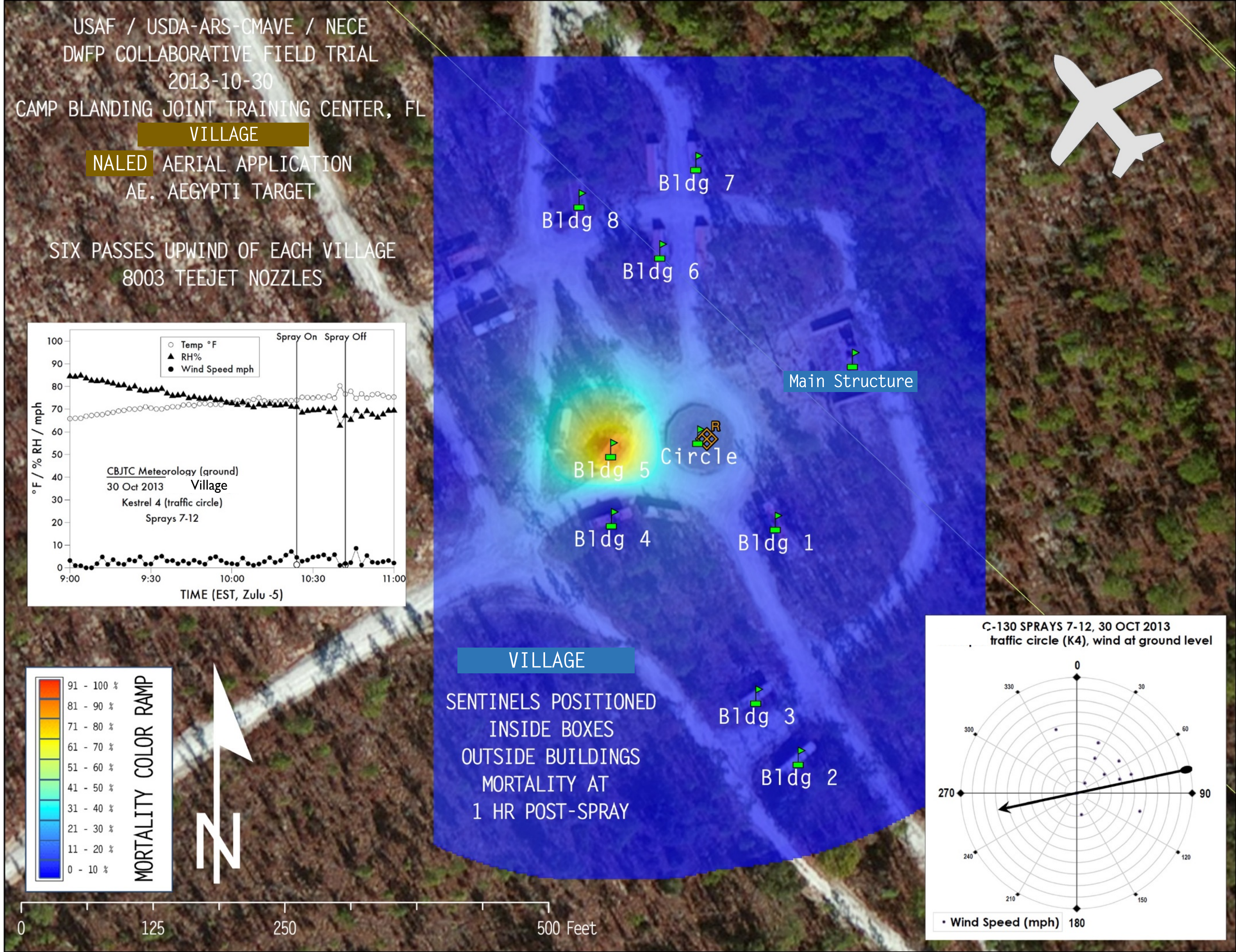

# Figure S38

30 October 2013

8003 nozzles

Village

4 hr mortality

Outdoors **IN BOX**

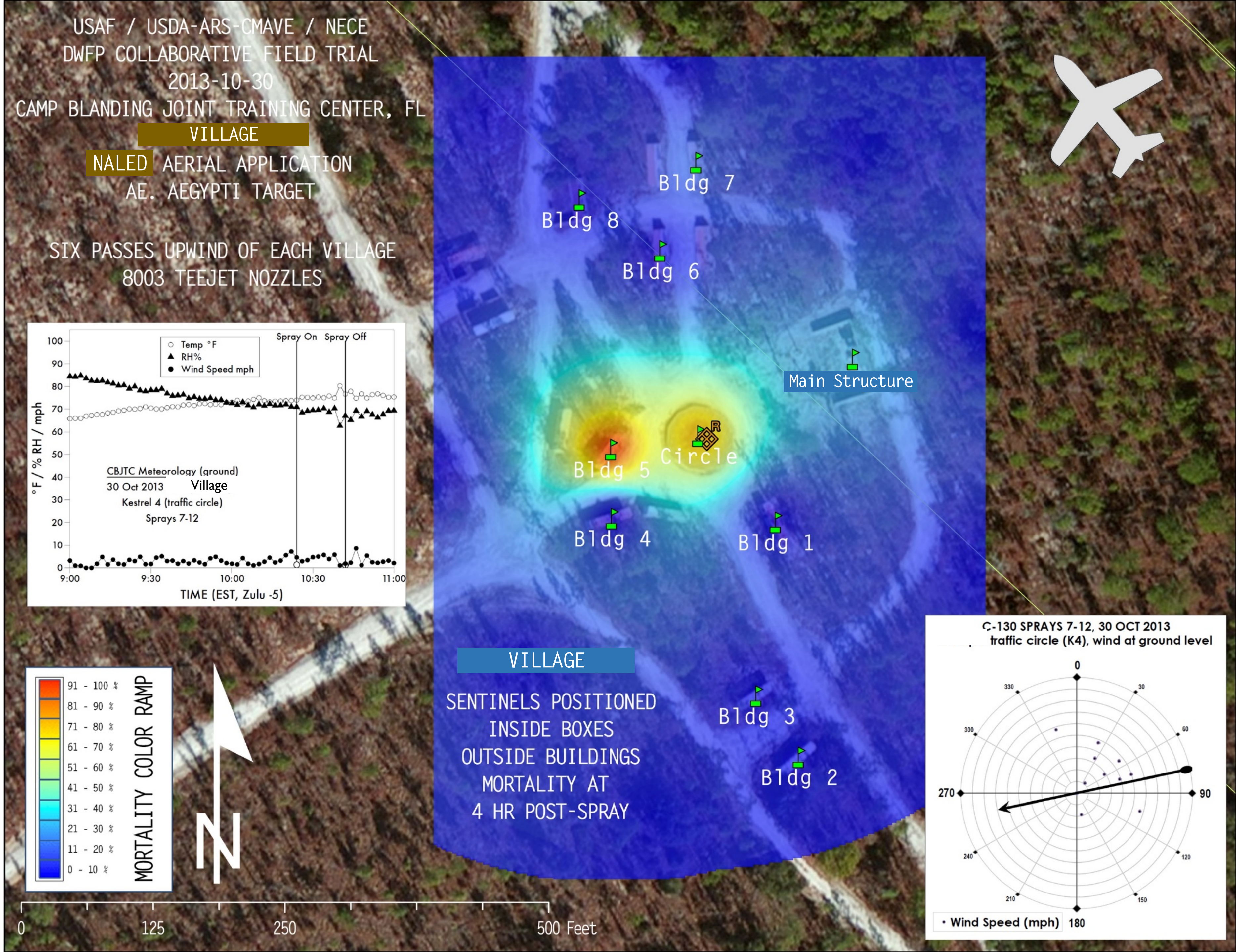

**Figure S39**
30 October 2013
8003 nozzles
Village
12 hr mortality
Outdoors **IN BOX**

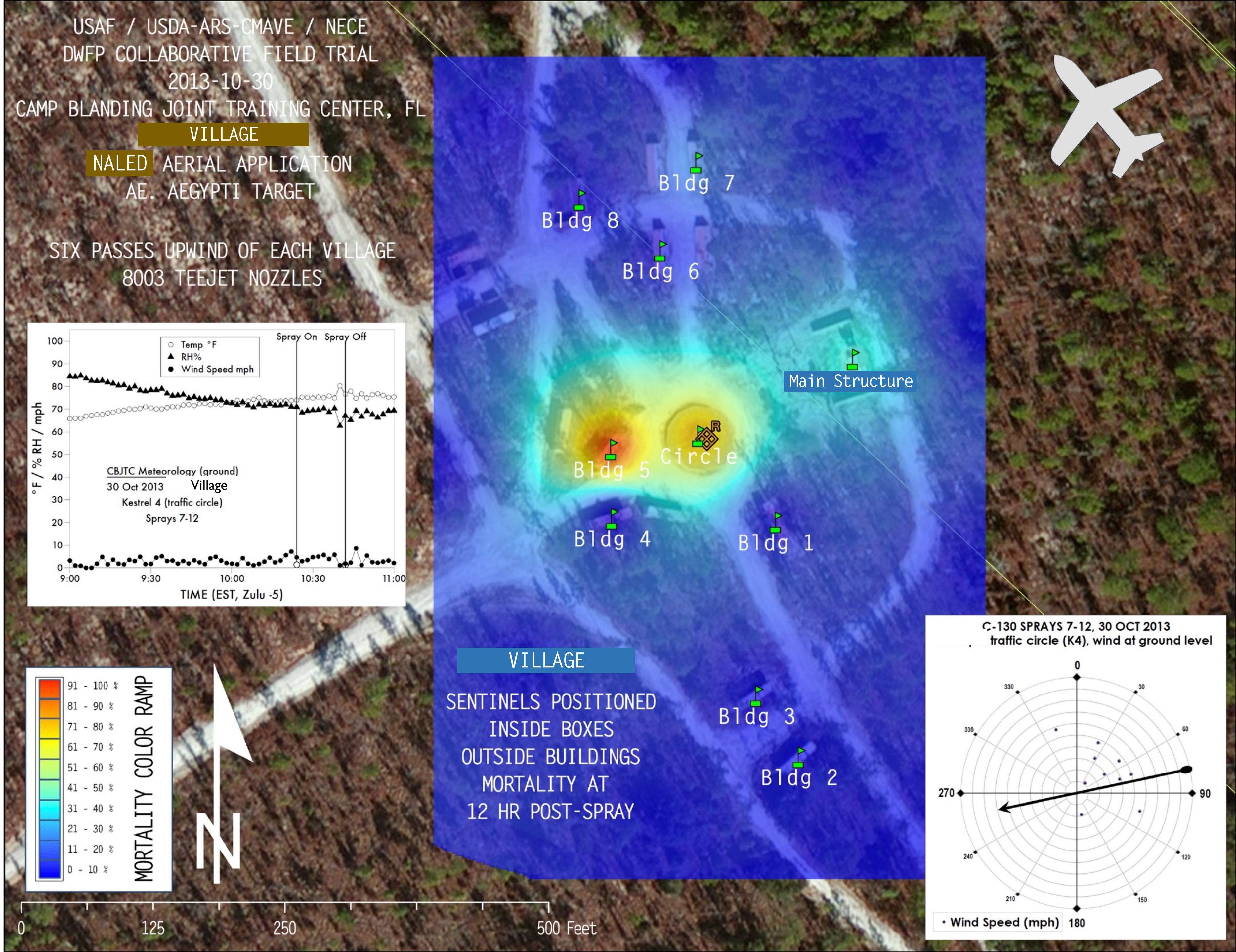

**Figure S40**
30 October 2013
8003 nozzles
Village
1 hr mortality
Outdoors **ON POLE**

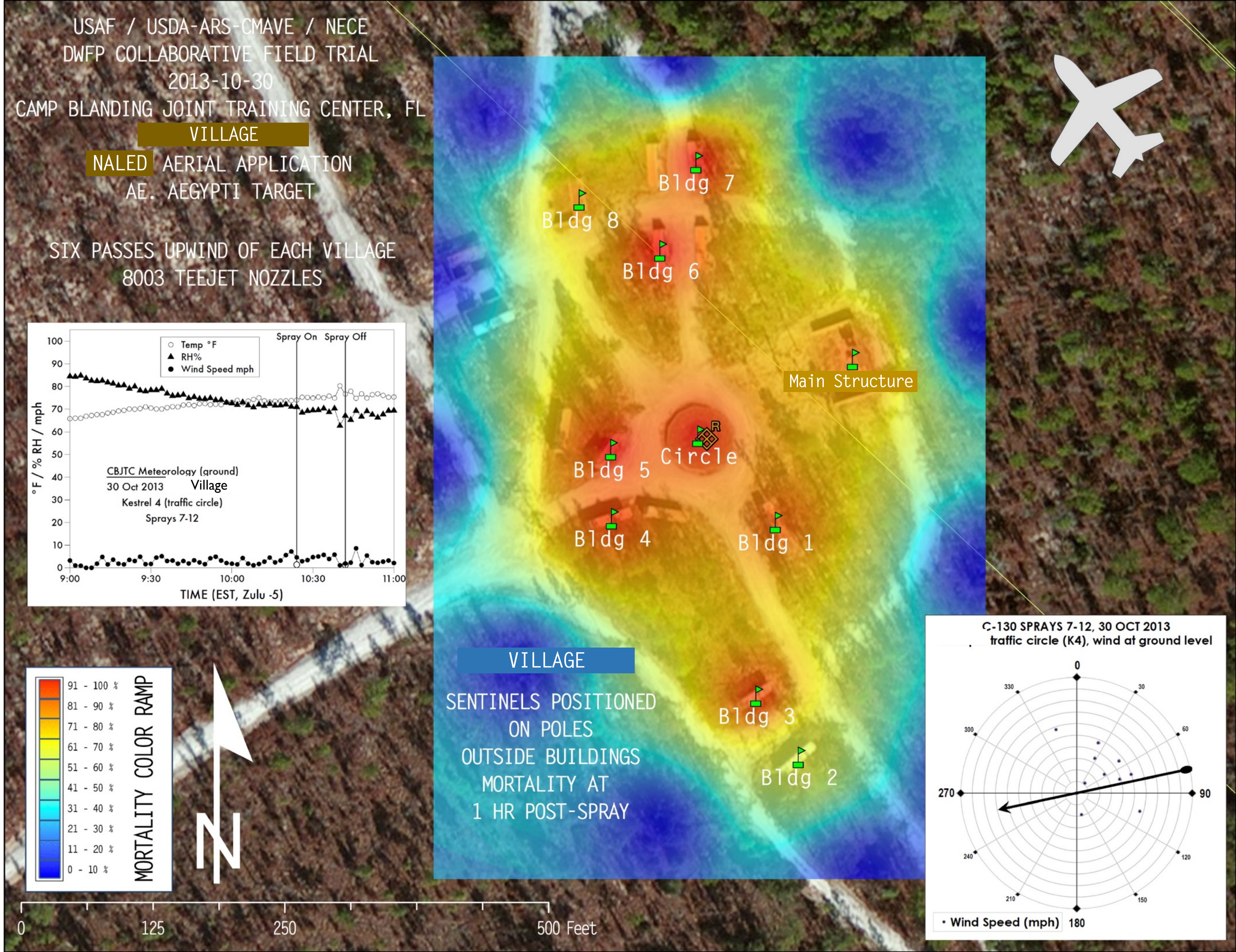

**Figure S41**
30 October 2013
8003 nozzles
Village
4 hr mortality
Outdoors **ON POLE**

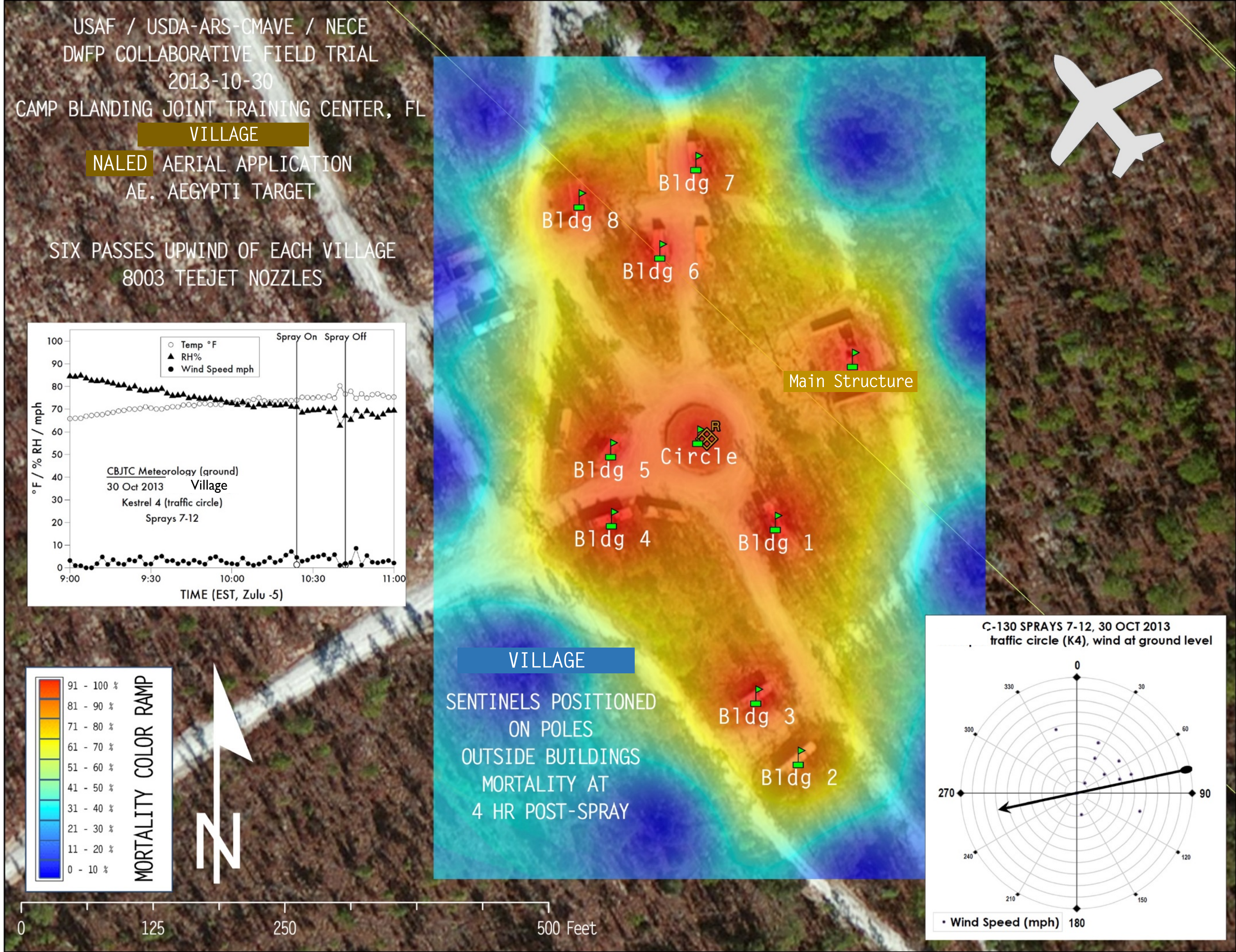

**Figure S42**
30 October 2013
8003 nozzles
Village
12 hr mortality
Outdoors **ON POLE**

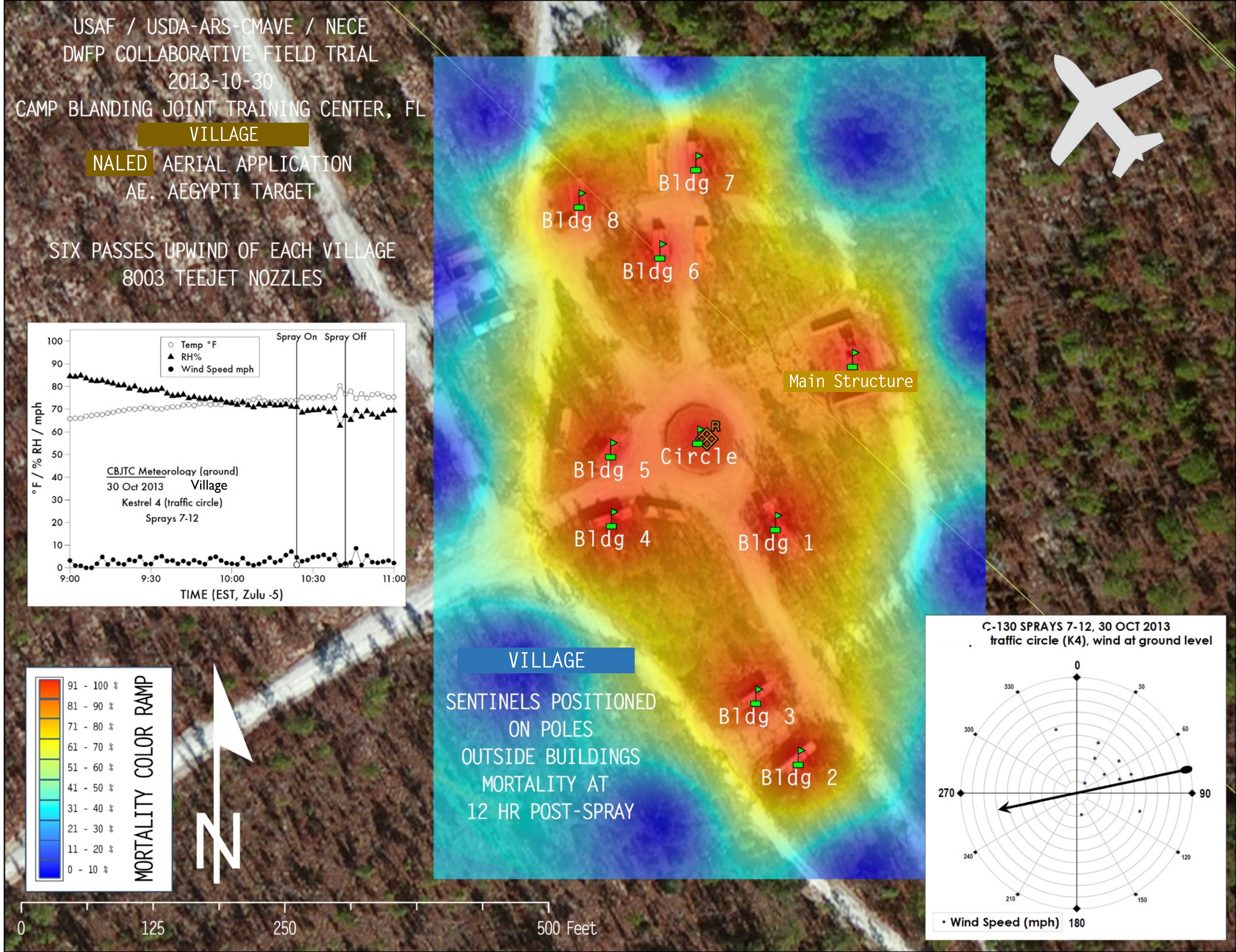

# Figure S43

30 October 2013

8003 nozzles

Village

1 hr mortality

Indoors **IN BOX**

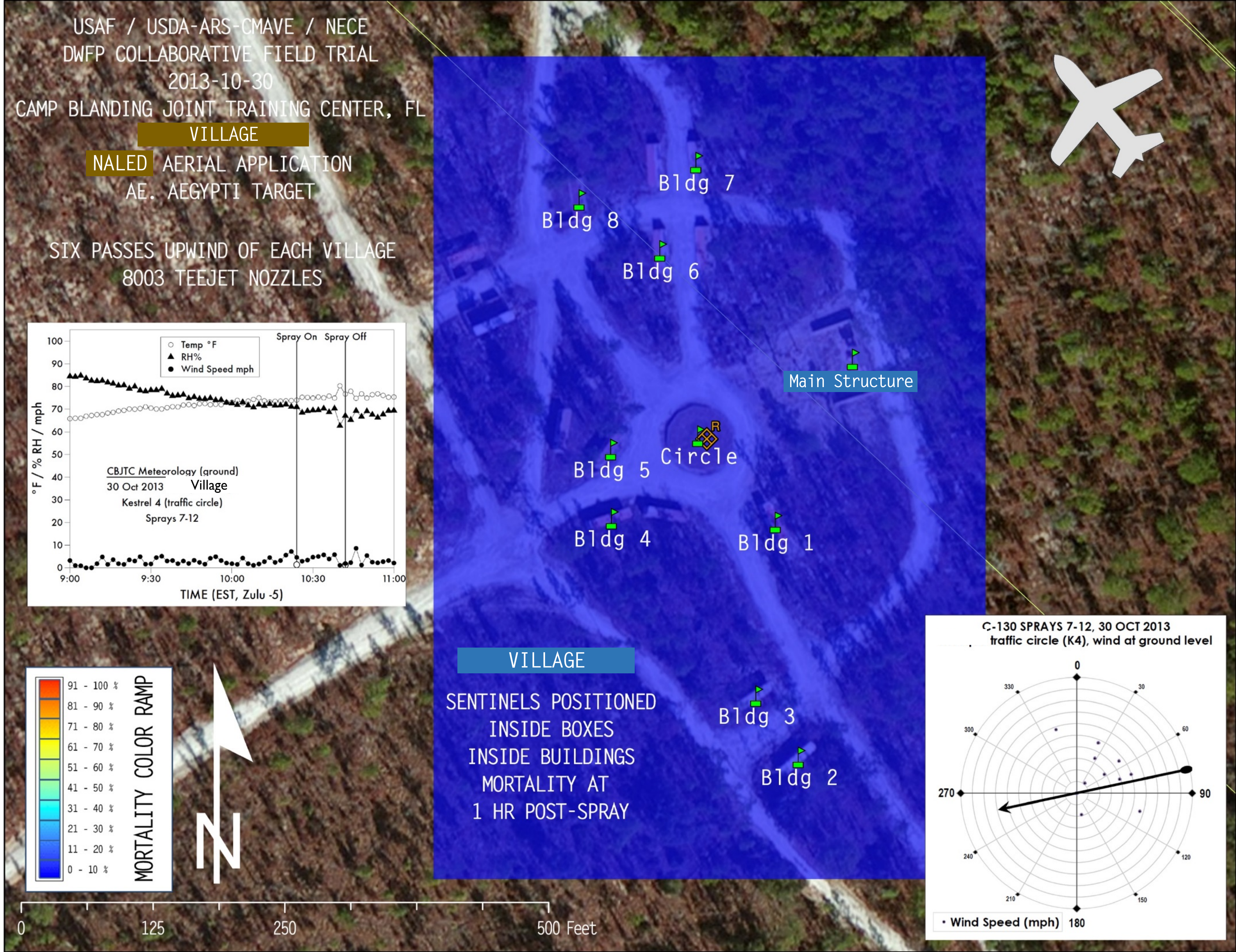

**Figure S44**
30 October 2013
8003 nozzles
Village
4 hr mortality
Indoors **IN BOX**

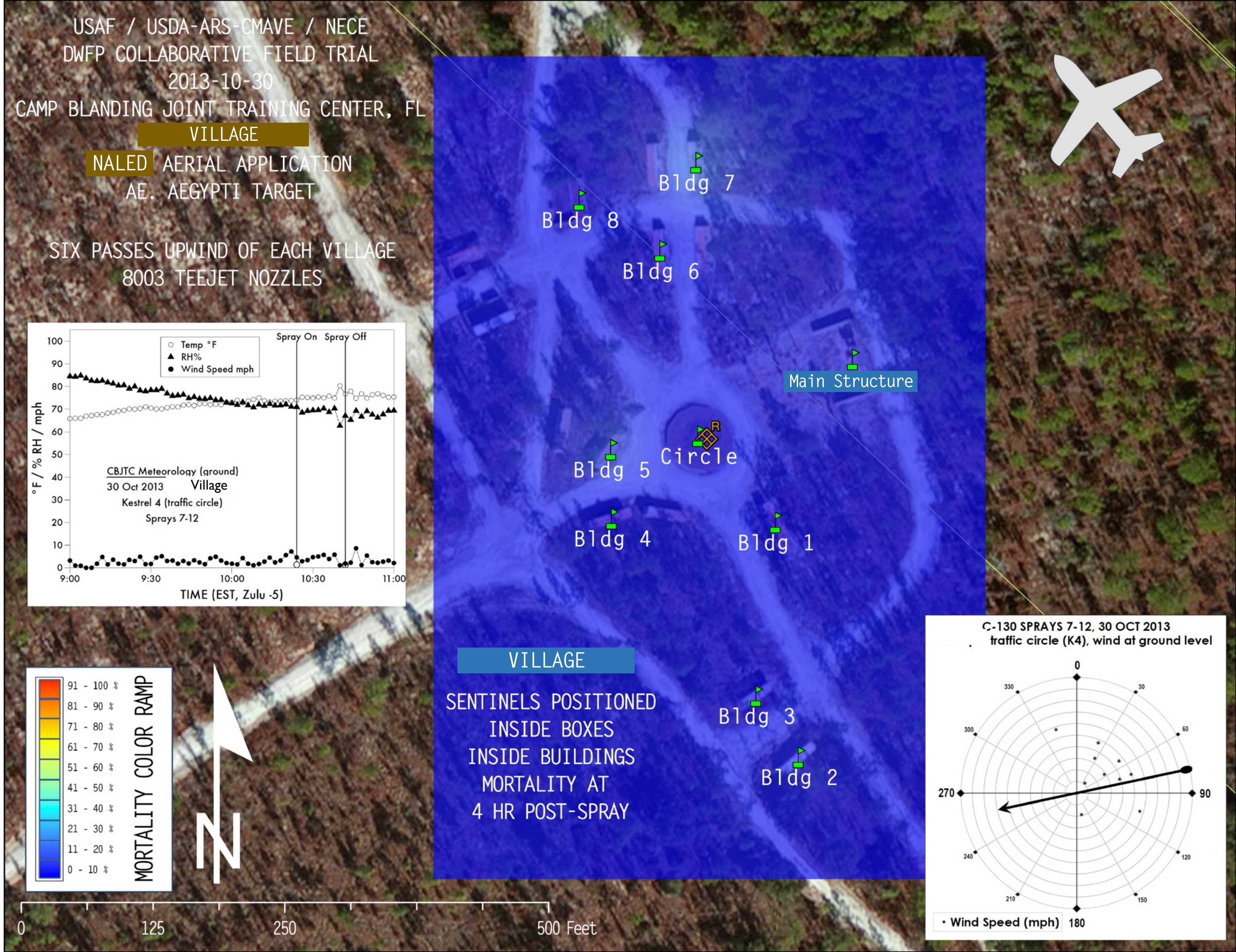

**Figure S45**
30 October 2013
8003 nozzles
Village
12 hr mortality
Indoors **IN BOX**

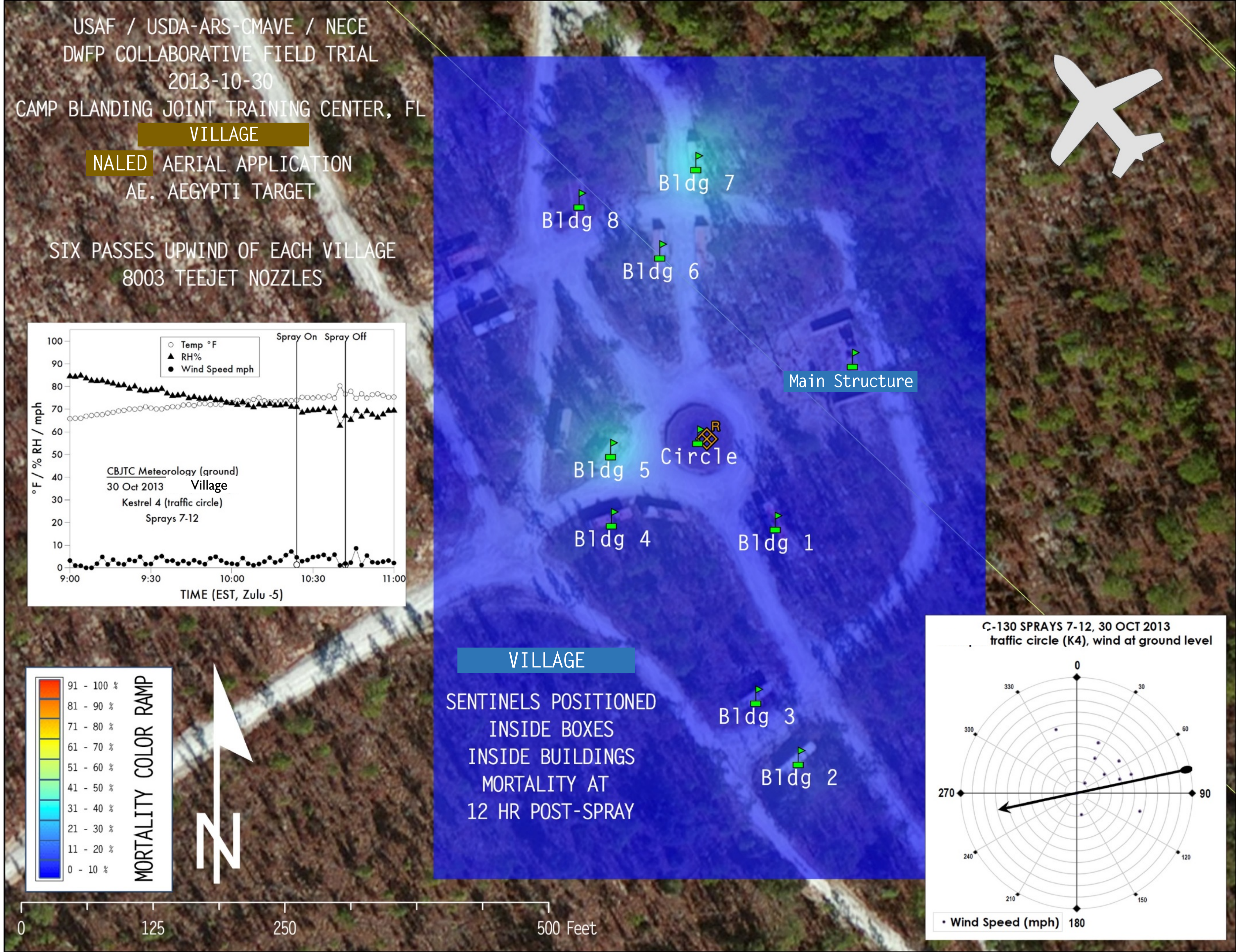

**Figure S46**
30 October 2013
8003 nozzles
Village
1 hr mortality
Indoors **ON FLOOR**

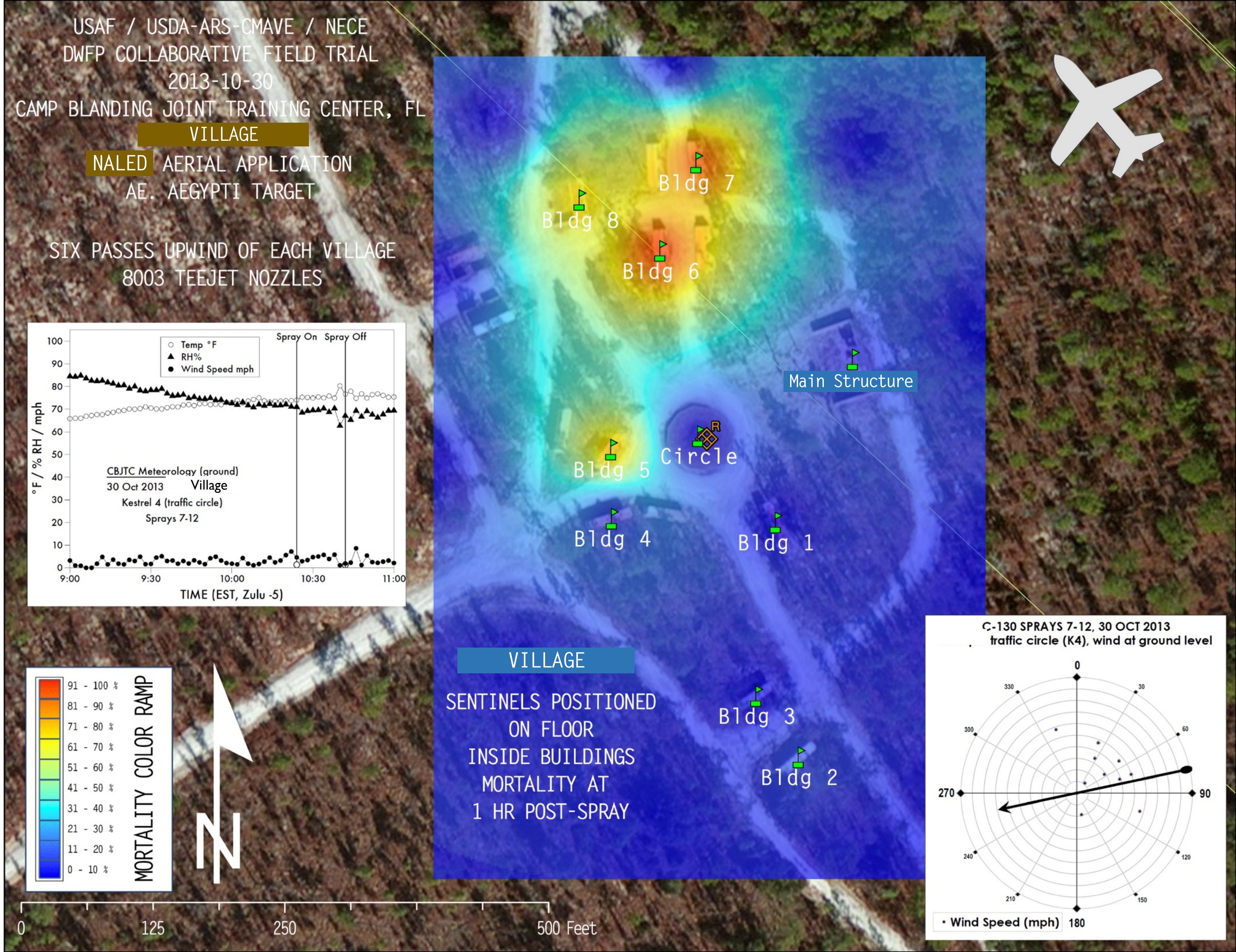

**Figure S47**
30 October 2013
8003 nozzles
Village
4 hr mortality
Indoors **ON FLOOR**

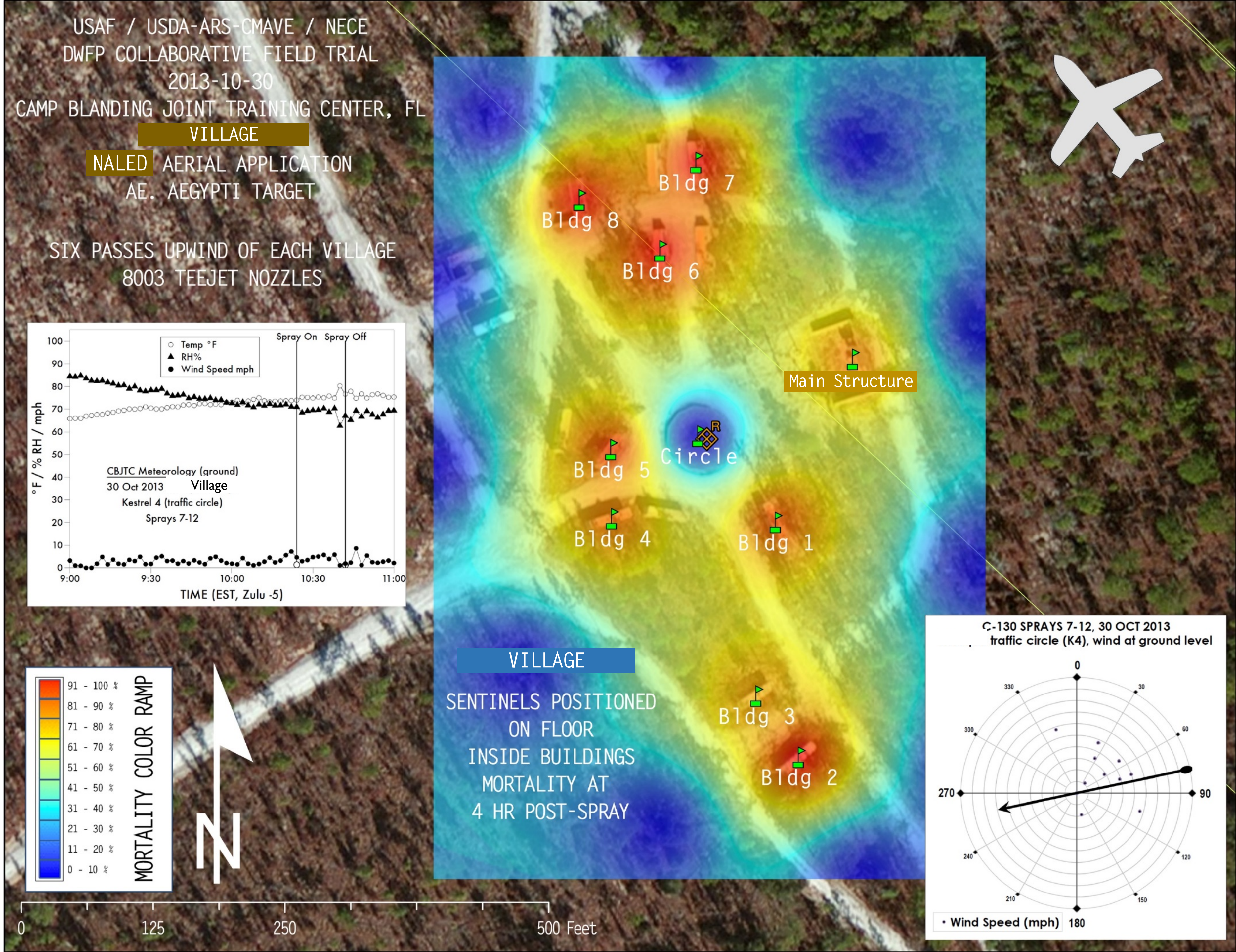

# Figure S48

30 October 2013

8003 nozzles

Village

12 hr mortality

Indoors **ON FLOOR**

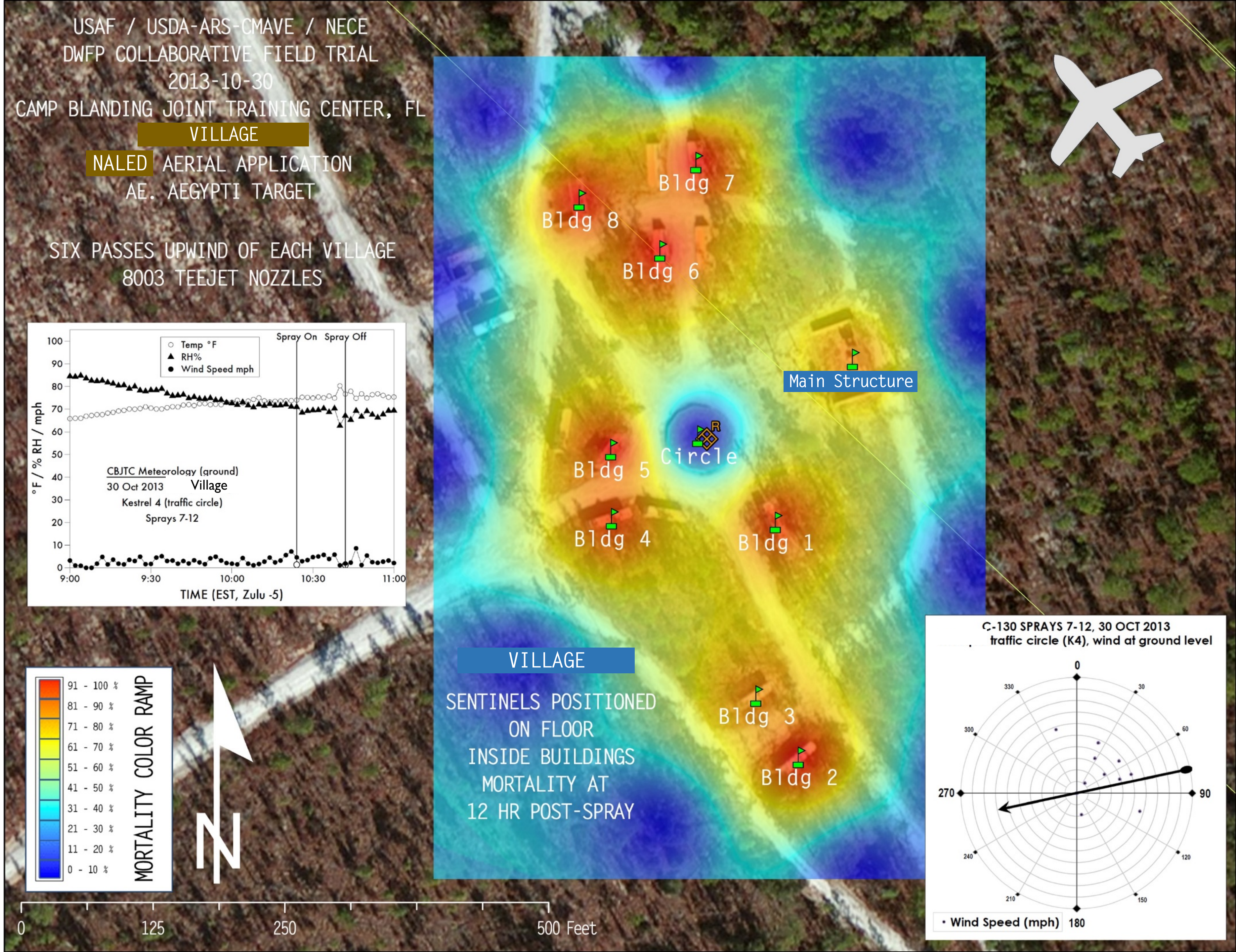

## SET E

S49–S50 Figs. Naled droplet densities, MOUT 29 Oct.

**Figure S49**
29 October 2013
8001 nozzles
MOUT South and nearby road Transect.

Droplet density (uncorrected) on 1/8-in spinning rods mounted on FLB spinners, in droplets per mm$^2$.

Note value of 33.62 in **red** with a black background to distinguish the FLB spinner located on a rooftop. All other values in red (with white background) represent FLB spinners at ground level.

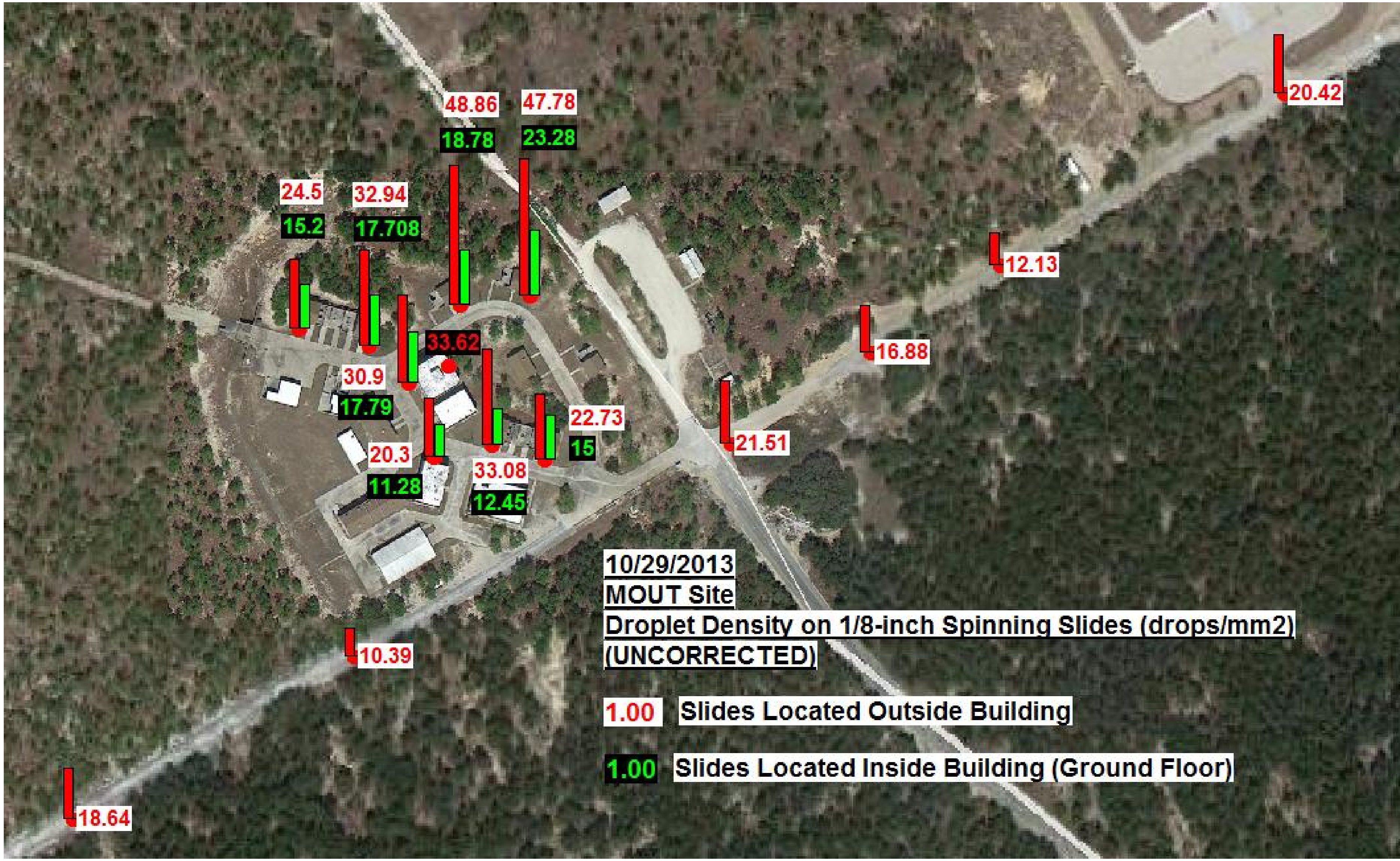

**Figure S50**
29 October 2013
8001 nozzles
MOUT South and nearby road Transect.

Droplet density (uncorrected) on 1/8-in spinning rods mounted on **outdoor** FLB spinners, in droplets per $mm^2$, represented as a 'heat map' to approximate transitions among values at point measurement locations.

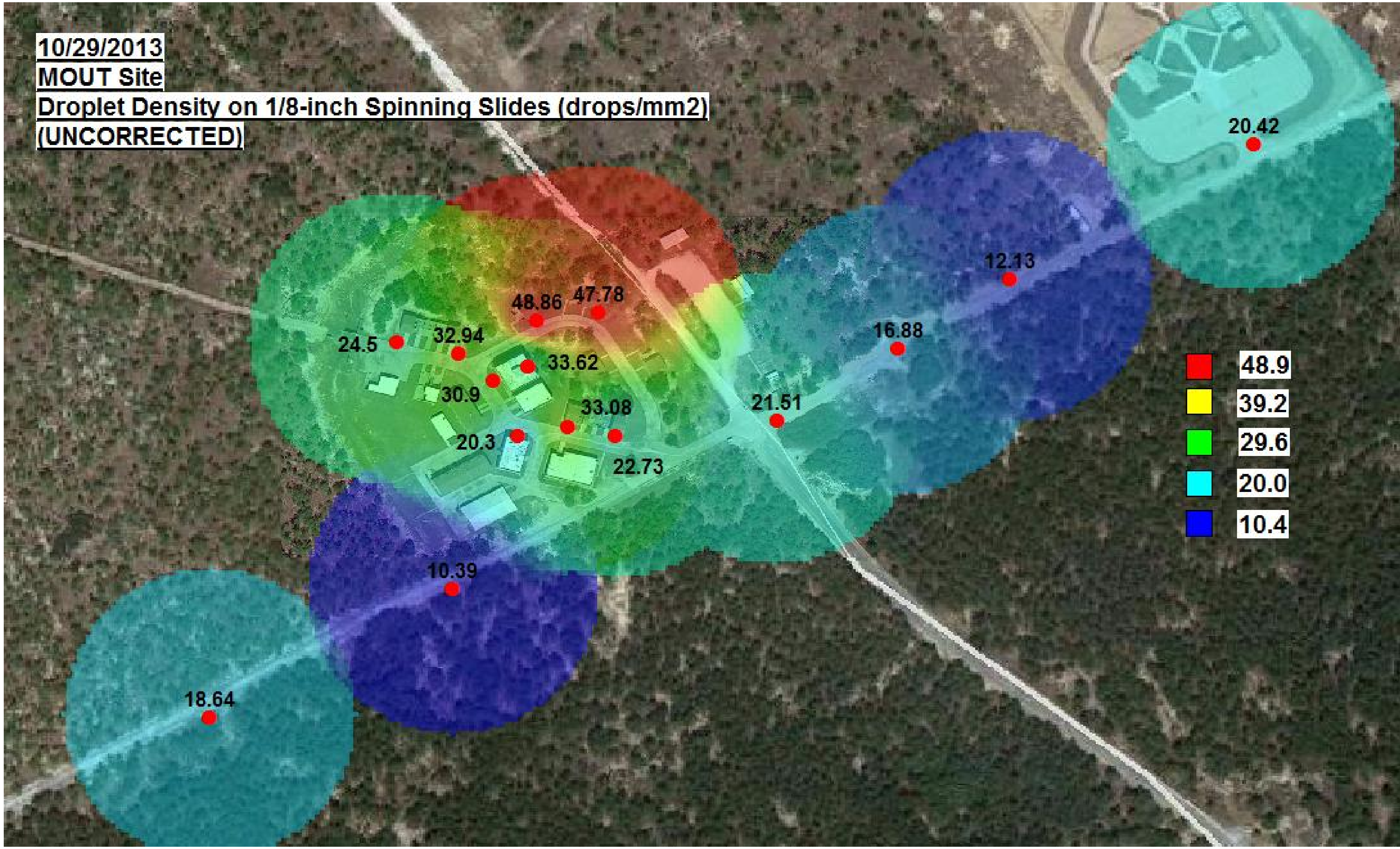

**SET F**

S51–S52 Figs. Naled droplet densities, MOUT 30 Oct.

**Figure S51**
30 October 2013
8003 nozzles
MOUT South and nearby road Transect.

Droplet density (uncorrected) on 1/8-in spinning rods mounted on FLB spinners, in droplets per $mm^2$.

Note value of 33.62 in red with a **yellow** background to distinguish the FLB spinner located on a rooftop. All other values in red (with white background) represent FLB spinners at ground level.

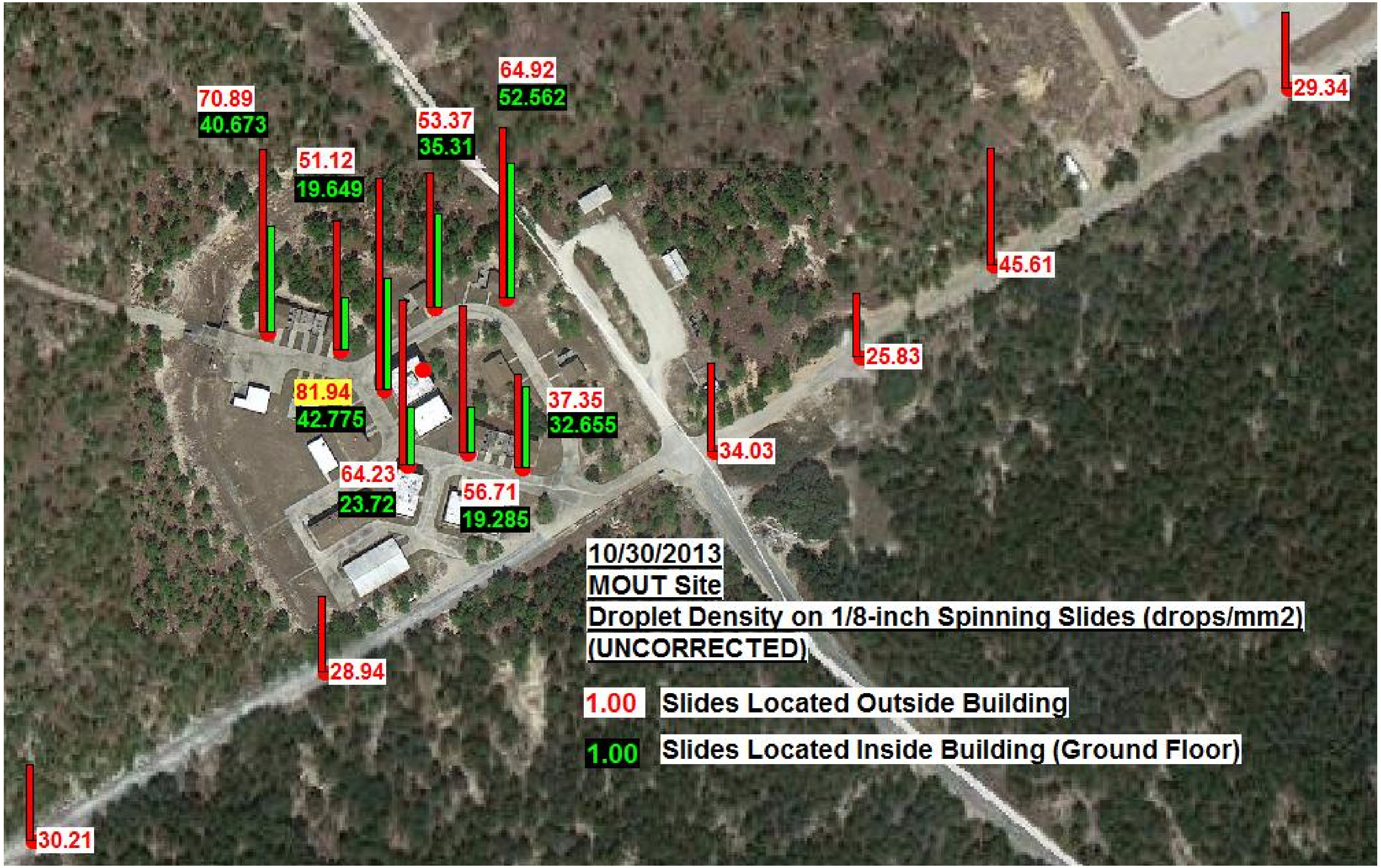

**Figure S52**
30 October 2013
8003 nozzles
MOUT South and nearby road Transect.

Droplet density (uncorrected) on 1/8-in spinning rods mounted on **outdoor** FLB spinners, in droplets per $mm^2$, represented as a 'heat map' to approximate transitions among values at point measurement locations.

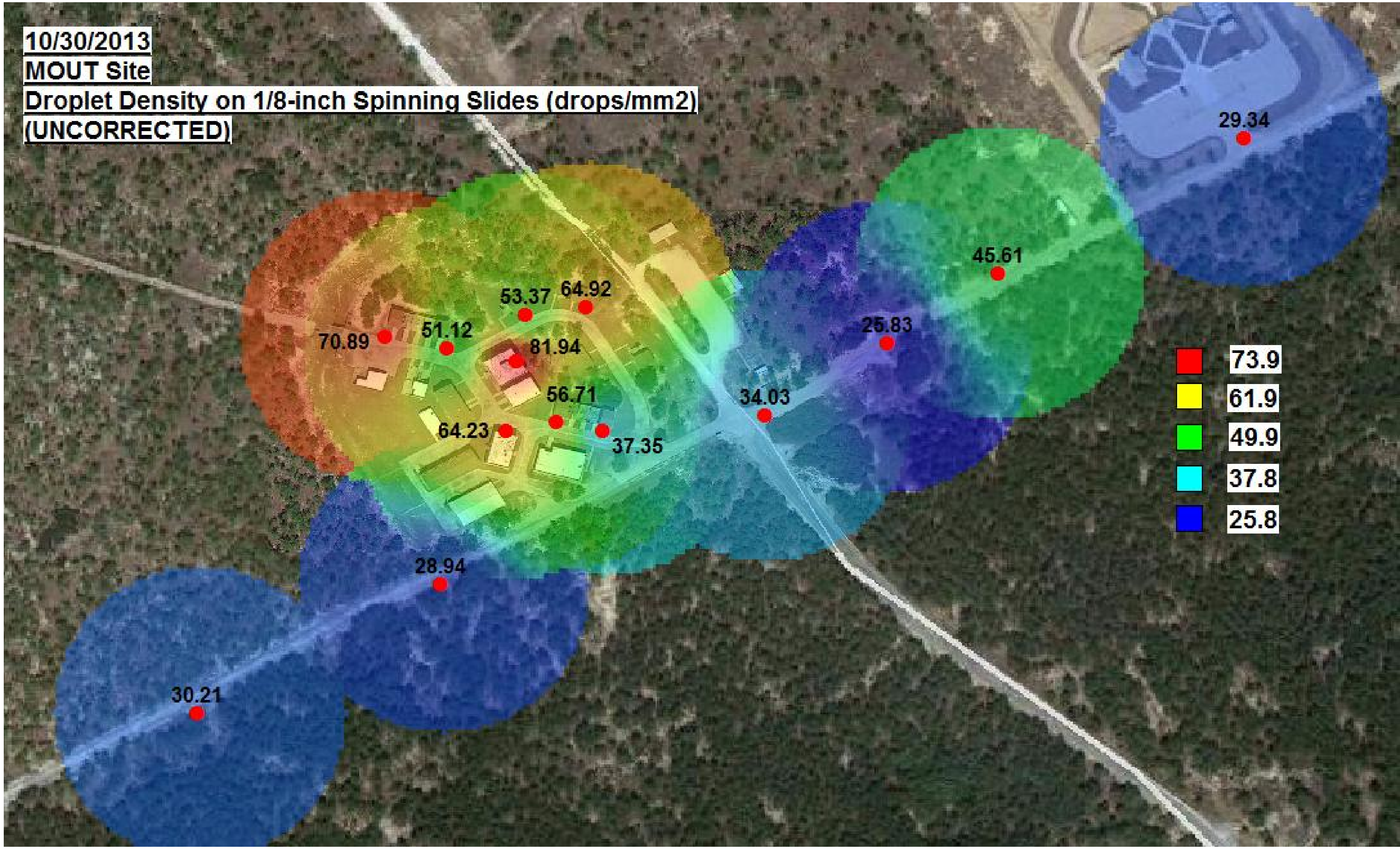

**SET G**

S53–S54 Figs. Naled droplet densities, Village 29 Oct.

**Figure S53**
29 October 2013
8001 nozzles
Village and nearby road
Transect

Droplet density (uncorrected) on 1/8-in spinning rods mounted on FLB spinners, in droplets per $mm^2$.

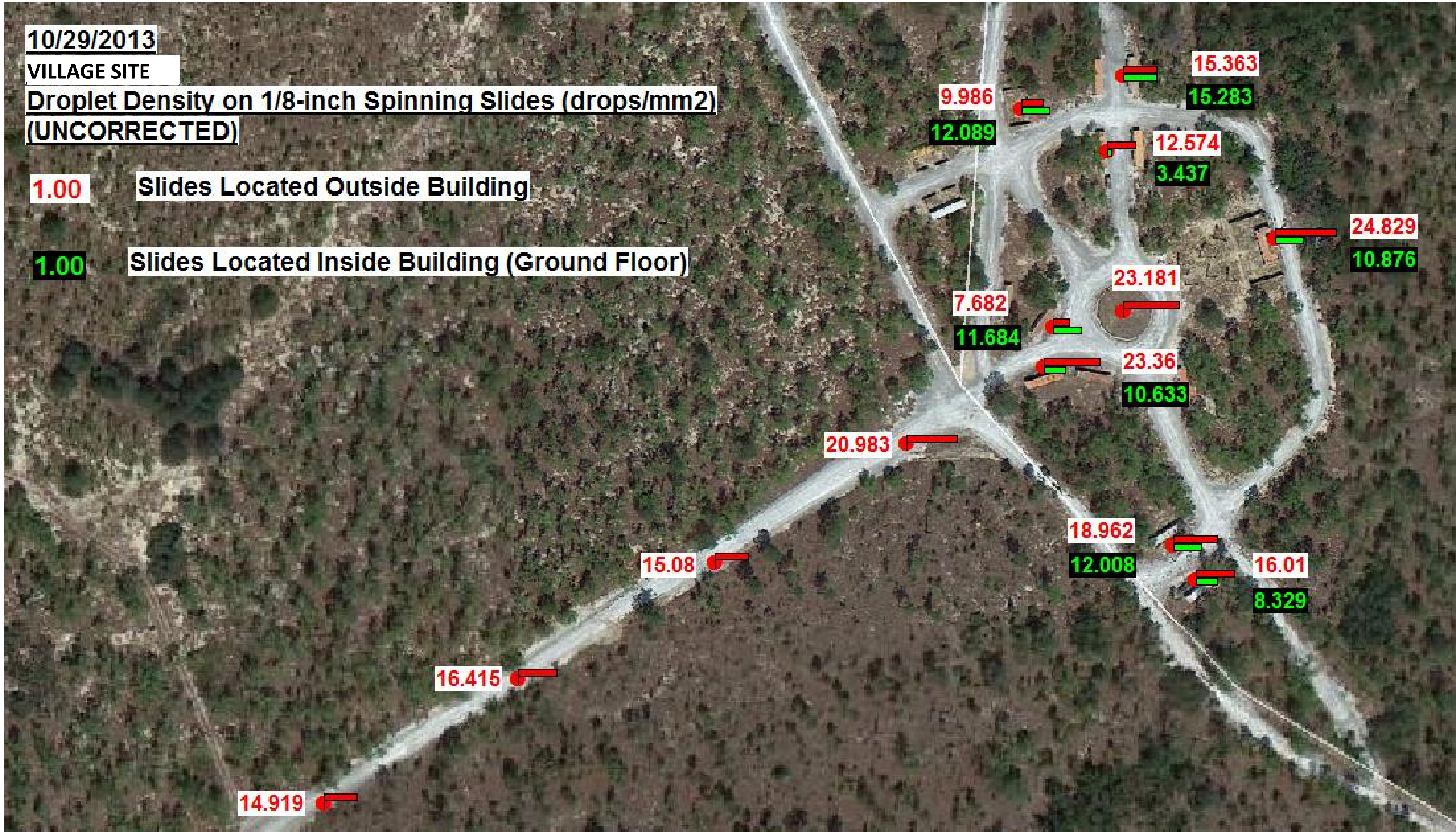

**Figure S54**
29 October 2013
8001 nozzles
Village and nearby road
Transect.

Droplet density (uncorrected) on 1/8-in spinning rods mounted on **outdoor** FLB spinners, in droplets per mm$^2$, represented as a 'heat map' to approximate transitions among values at point measurement locations.

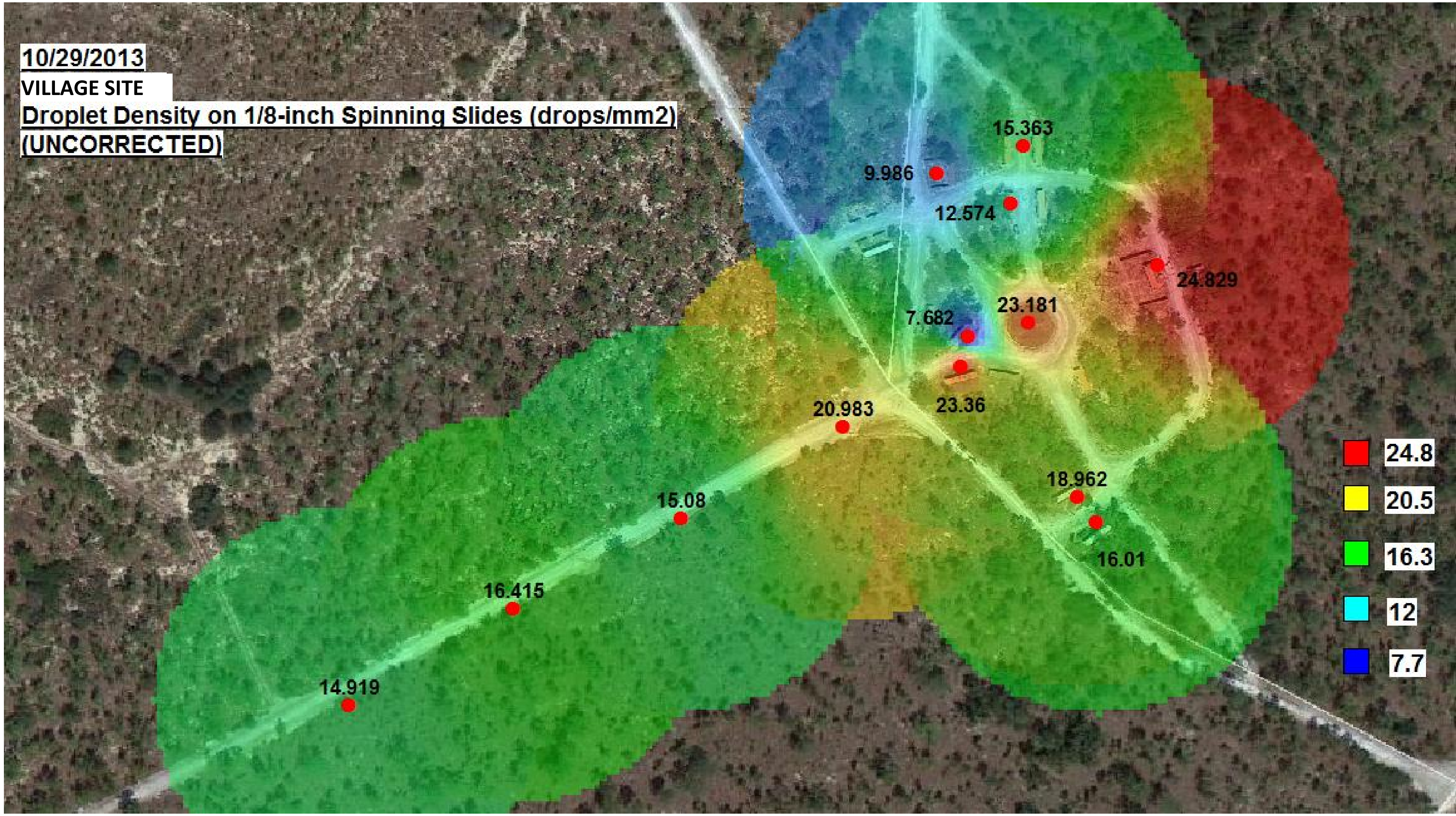

## SET H

S55–S56 Figs. Naled droplet densities, Village 30 Oct.

**Figure S55**
30 October 2013
8003 nozzles
Village and nearby road
Transect

Droplet density (uncorrected) on 1/8-in spinning rods mounted on FLB spinners, in droplets per mm².

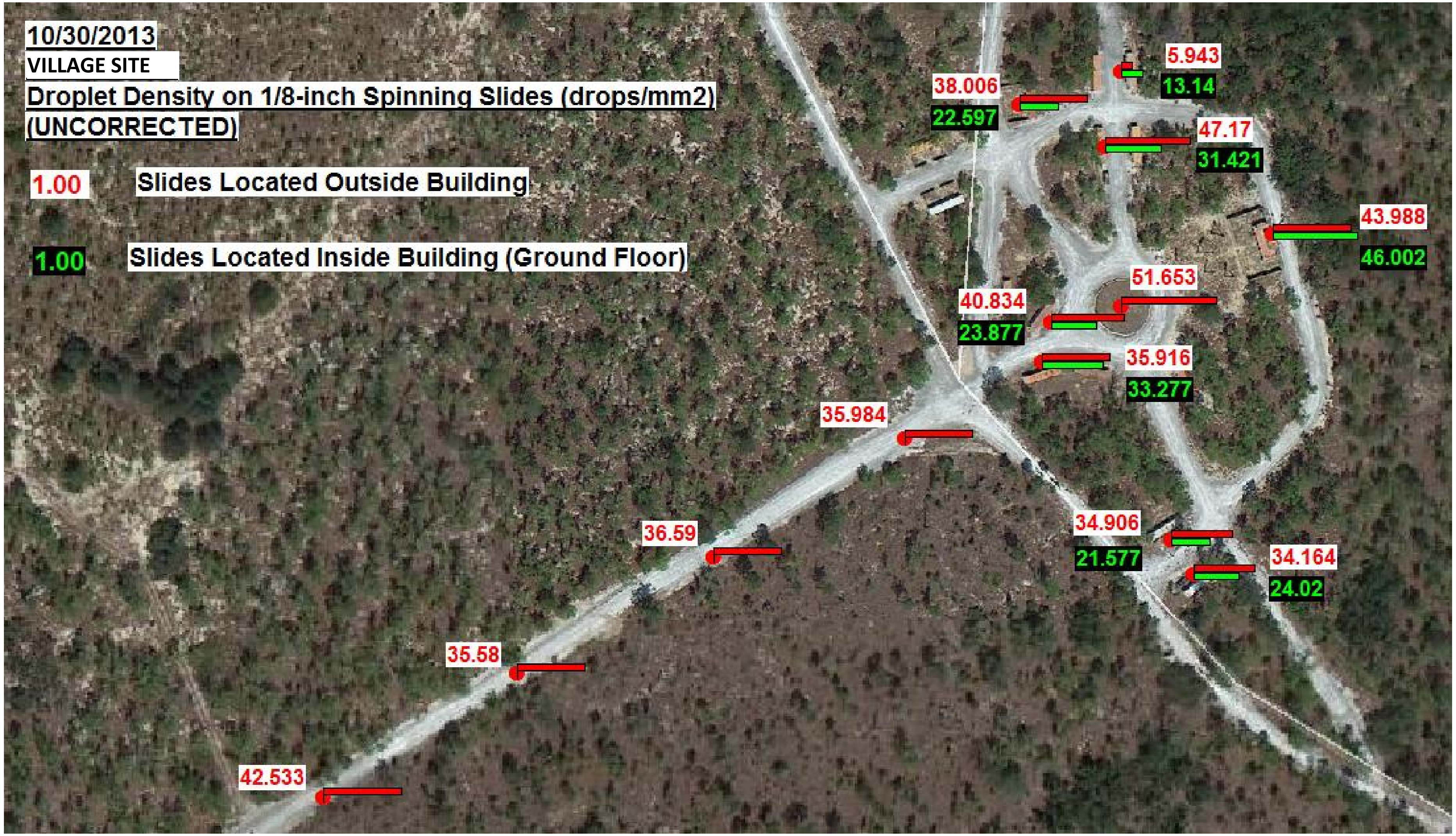

**Figure S56**
30 October 2013
8003 nozzles
Village and nearby road
Transect.

Droplet density (uncorrected) on 1/8-in spinning rods mounted on **outdoor** FLB spinners, in droplets per $mm^2$, represented as a 'heat map' to approximate transitions among values at point measurement locations.

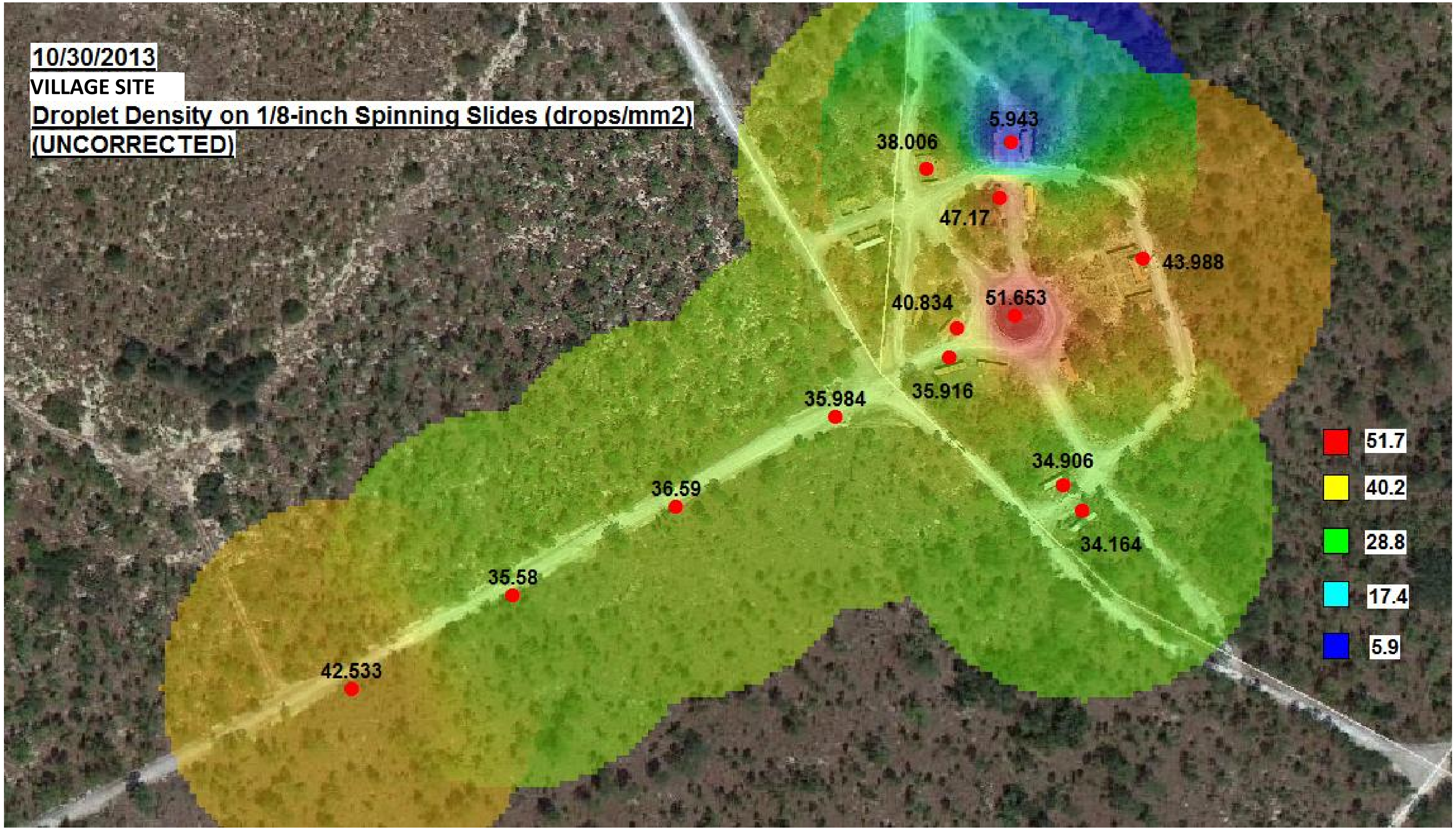

**SET I**

S57–S59 Figs. Naled droplet densities, Transects 29 Oct.

**Figure S57**

29 October 2013

8001 nozzles

Road Transects

1 hr mortality

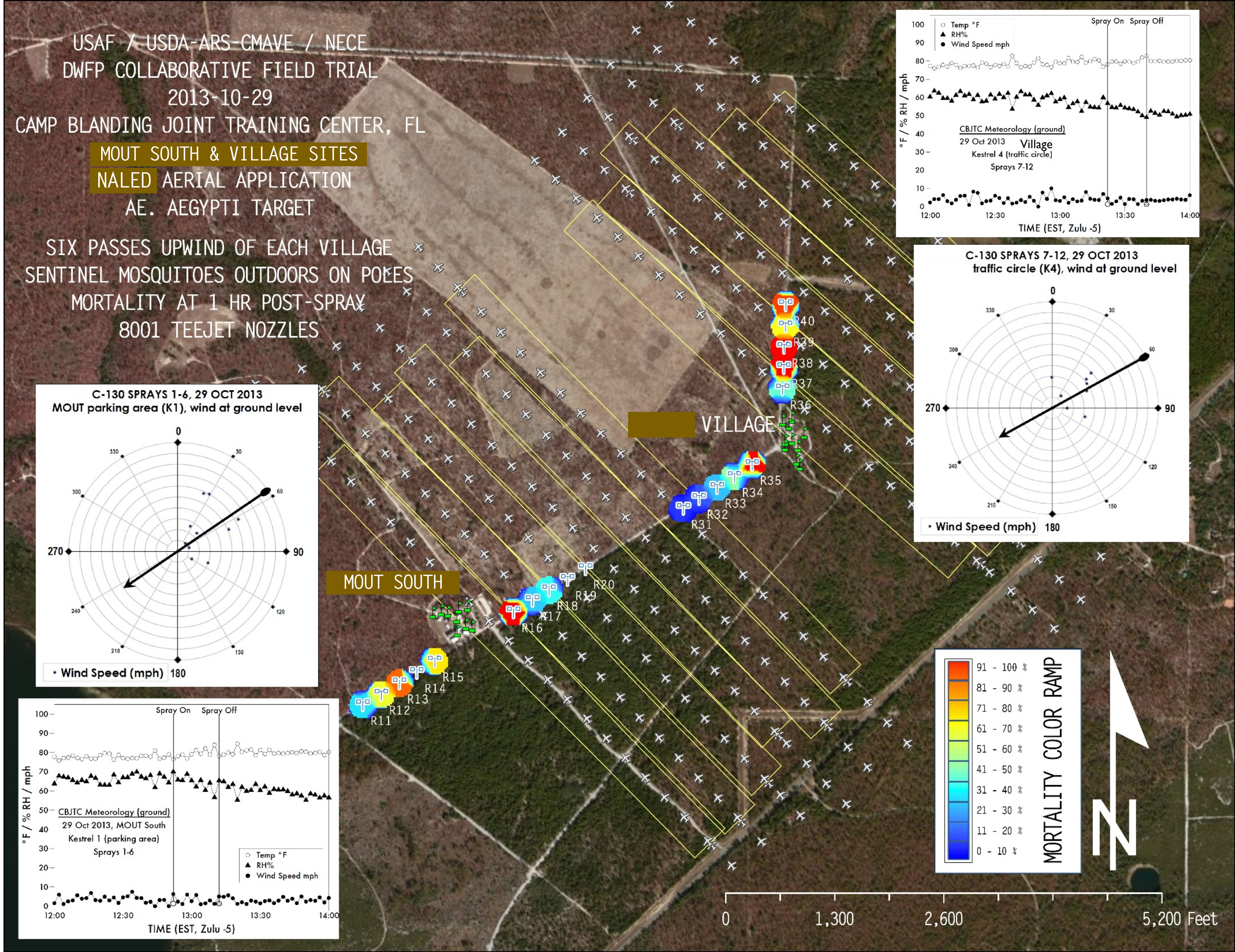

# Figure S58

29 October 2013

8001 nozzles

Road Transects

4 hr mortality

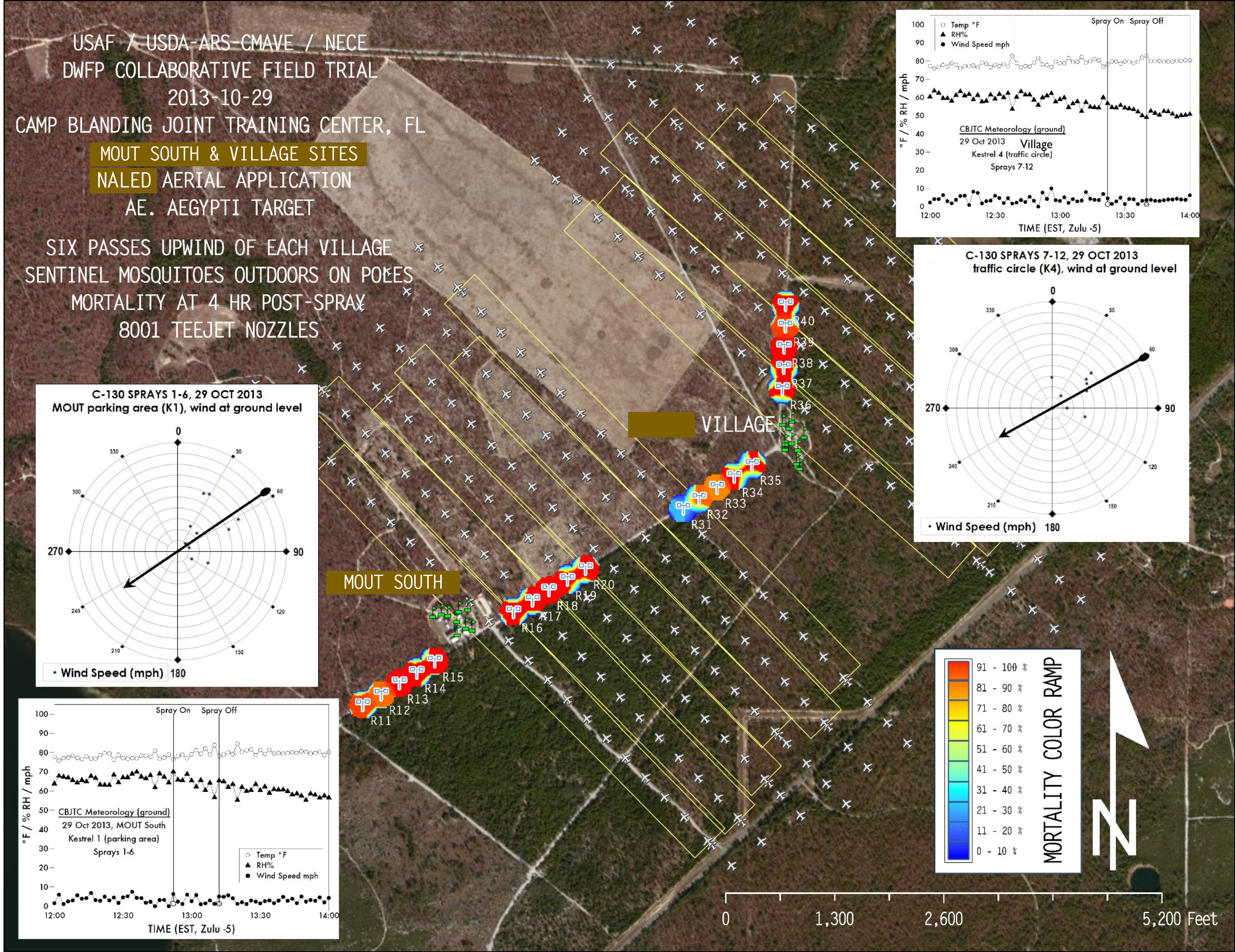

# Figure S59

29 October 2013

8001 nozzles

Road Transects

12 hr mortality

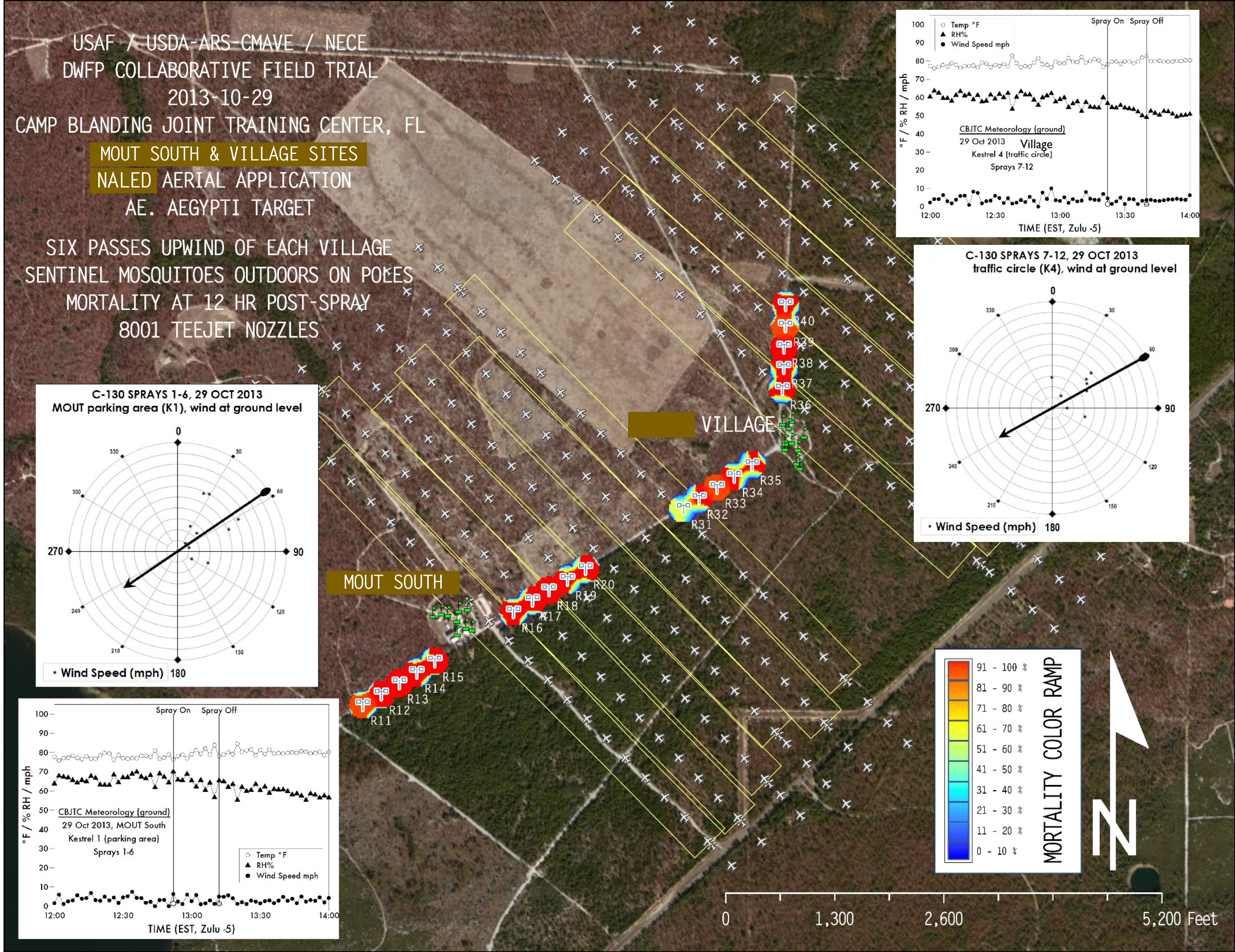

## SET J

S60–S62 Figs. Naled droplet densities, Transects 30 Oct.

**Figure S60**
30 October 2013
8003 nozzles
Road Transects
1 hr mortality

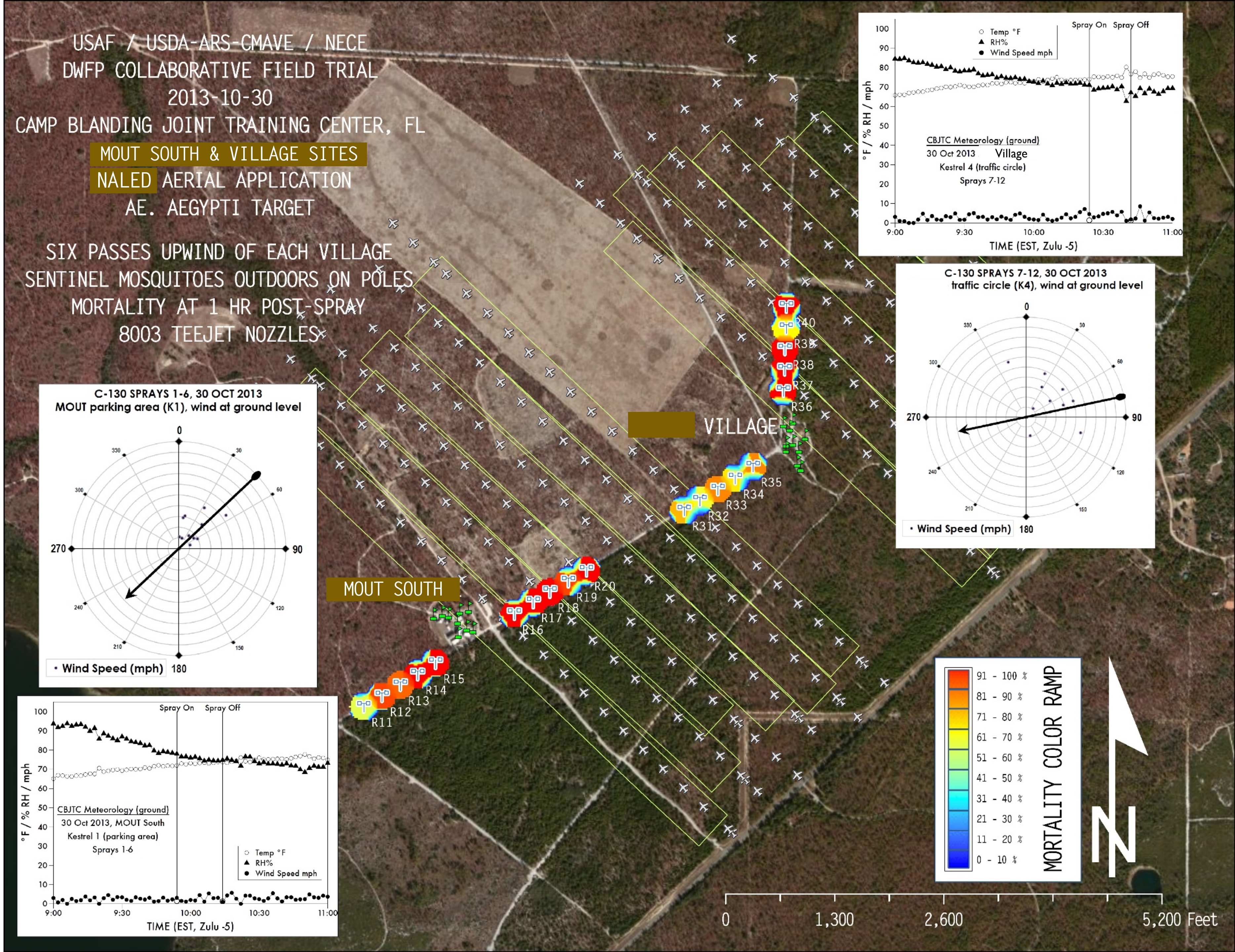

# Figure S61

30 October 2013

8003 nozzles

Road Transects

4 hr mortality

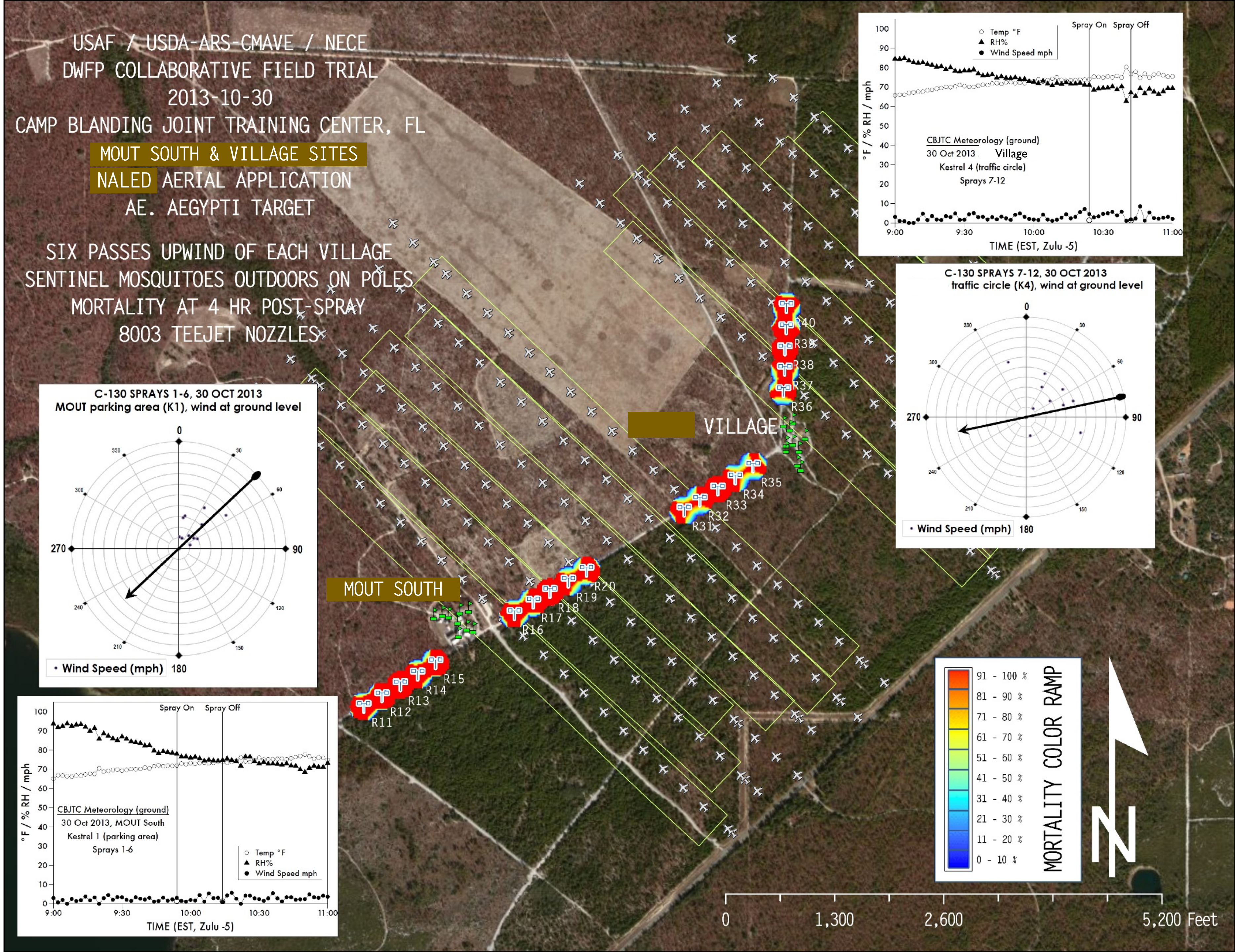

**Figure S62**
30 October 2013
8003 nozzles
Road Transects
12 hr mortality

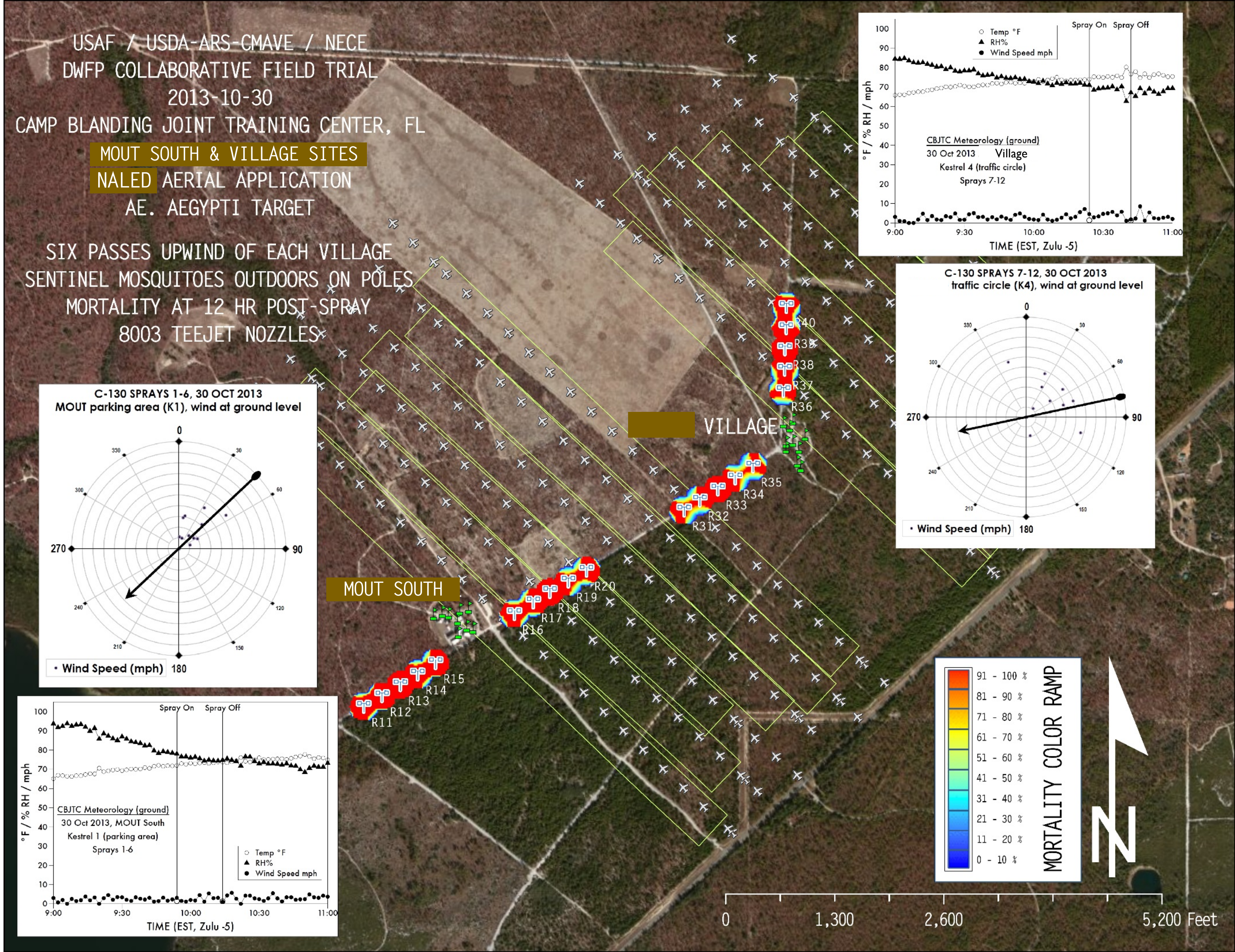

Supplement: S1 Fig — (PDF) [file pone.0191555.s001.pdf]
