# Supplementary material for: Aerial ULV control of Aedes aegypti with naled (Dibrom) inside simulated rural village and urban cryptic habitats
Source: PLoS One. 2018 Jan 19;13(1):e0191555. doi: 10.1371/journal.pone.0191555 (PMC5774805; doi:10.1371/journal.pone.0191555)
Supplement: S1 Table — Doors and windows were propped open at all structures unless indicated; Wax Candle and Bread Market structures had no doors or window shutters present and were left open. Percent mortality at 1 hr marked in bold with dagger indicates ants present at time of sentinel cage pickup. (PDF) [file pone.0191555.s002.pdf]

Supplementary information for Britch et al. Aerial ULV control of *Aedes aegypti* with naled (Dibrom) inside simulated rural village and urban cryptic habitats**.**

S1 Table. **Abbott-corrected sentinel adult *Ae. aegypti* mosquito percent mortality at 1, 4, and 12 hr post-spray following the 29-30 October aerial naled applications over the MOUT South site.** Doors and windows were propped open at all structures unless indicated; Wax Candle and Bread Market structures had no doors or window shutters present and were left open. Percent mortality at 1 hr marked in bold with dagger indicates ants present at time of sentinel cage pickup.

MOUT South location	Type	Position	29 October			30 October		
			1 hr	4 hr	12 hr	1 hr	4 hr	12 hr
**Wax Candle**	outdoors	inside box	5.0	4.5	8.6	0	35.2	51.2
		on pole	40.0	100	100	100	100	100
	indoors	inside box	0	0	13.7	0	2.6	7.3
		on floor	0	24.6	39.1	**0†**	100	100
**Bread Market**	outdoors	inside box	5.0	4.5	8.6	0	100	100
		on pole	100	100	100	100	100	100
	indoors	inside box	0	0	0	0	79.5	79.4
		on floor	**5.0†**	79.9	84.8	**68.0†**	100	100
**Bazaar (closed up)**	outdoors	inside box	0	74.9	89.8	0	2.6	2.1
		on pole	0	100	100	0	100	100
	indoors	inside box	0	24.6	69.5	0	0	0
		on floor	0	34.7	69.5	0	74.4	74.2
**Meat Market**	outdoors	inside box	0	0	3.5	0	2.6	7.3
		on pole	0	100	100	0.0	100	100
	indoors	inside box	0	0	0	0	0	2.1
		on floor	0	39.7	64.5	0	100	100
**Bridge**	surface	inside box	**95.0†**	95.0	94.9	0	69.2	79.4
		on pole	100	100	100	100	100	100
	underneath	inside box	0	89.9	94.9	0	2.8	2.4
		on pole	100	100	100	100	100	100
**Holiday Hotel**	outdoors	inside box	10.0	59.8	64.5	0	54.4	77.1
		on pole	100	100	100	100	100	100
	indoors (1st floor)	inside box	0.0	4.5	3.5	0	48.7	74.2
		on floor	0.0	0	3.5	0	100	100
	indoors (2nd floor)	inside box	5.0	4.5	3.5	0	89.7	89.7
		on floor	5.3	4.8	9.1	4.1	83.8	89.2
**American Hotel (closed up)**	outdoors	inside box	5.0	49.7	74.6	0	100	100
		on pole	90.0	100	100	100	100	100
	indoors (1st floor)	inside box	0	4.5	3.5	0	12.8	27.9
		on floor	0	0	0	0	0	7.3
	indoors (2nd floor)	inside box	0	0	0	0	0	63.9
		on floor	0	27.4	60.5	0	7.7	17.6
**Fruit Market (closed up)**	outdoors	inside box	0	0	0	0	100	100
		on pole	90.0	100	100	100	100	100
	indoors	inside box	0.0	0	23.8	0	94.3	94.3
		on floor	0.0	0	3.5	0	94.9	100.0
**Jose Cantina**	outdoors	inside box	0.0	4.5	8.6	0	100	100
		on pole	90.0	100	100	100	100	100
	indoors	inside box	0.0	4.5	3.5	0	0	22.7
		on floor	0.0	0	3.5	0	43.0	59.9
