# Supplementary material for: Aerial ULV control of Aedes aegypti with naled (Dibrom) inside simulated rural village and urban cryptic habitats
Source: PLoS One. 2018 Jan 19;13(1):e0191555. doi: 10.1371/journal.pone.0191555 (PMC5774805; doi:10.1371/journal.pone.0191555)
Supplement: S2 Table — All structures were unmodified with windows and doors completely open. Percent mortality at 1 hr marked in bold with dagger indicates ants present at time of sentinel cage pickup. (PDF) [file pone.0191555.s003.pdf]

Supplementary information for Britch et al. Aerial ULV control of *Aedes aegypti* with naled (Dibrom) inside simulated rural village and urban cryptic habitats**.**

S2 Table. Abbott-corrected sentinel adult *Ae. aegypti* mosquito percent mortality at 1, 4, and 12 hr post-spray following the 29-30 October aerial naled applications over the Village site.

All structures were unmodified with windows and doors completely open. Percent mortality at 1 hr marked in bold with dagger indicates ants present at time of sentinel cage pickup.

			29 October			30 October		
**Village Location**	**Type**	**Position**	**1 hr**	**4 hr**	**12 hr**	**1 hr**	**4 hr**	**12 hr**
**Roundabout**	outdoors	inside box	15.0	100	100	13.9	74.4	74.2
		on pole	100	100	100	100	100	100
**Main Structure**	outdoors (courtyard)	inside box	**0.0†**	19.6	69.5	0	24.4	34.9
		on pole	**60.0†**	100	100	**87.3†**	100	100
	indoors (1st floor)	inside box	**0†**	0	0	3.8	2.6	2.1
		on floor	**0†**	29.6	54.3	0	65.8	65.7
	indoors (2nd floor)	inside box	0	0	0	0	2.6	2.1
		on floor	10.0	74.9	94.9	8.9	100	100
**Building #1 residence**	outdoors	inside box	**95.0†**	95.0	94.9	0	0	0
		on pole	75.0	100	100	89.3	100	100
	indoors	inside box	0	0	0	0	0	0
		on floor	0	68.3	78.6	0	89.7	94.8
**Building #2 residence**	outdoors	inside box	**50.0†**	49.7	59.4	0	0	0
		on pole	95.0	100	100	60.6	88.6	100
	indoors	inside box	0	100	100	0	2.6	7.3
		on floor	0	84.9	100	9.4	100	100
**Building #3 residence**	outdoors	inside box	**0†**	0	0	0	0	0
		on pole	100	100	100	94.7	100	100
	indoors	inside box	0	0	8.6	0	0	0
		on floor	0	29.6	39.1	0	78.4	83.7
**Building #4 residence**	outdoors	inside box	0	0	13.7	0	0	0
		on pole	0	100	100	94.4	100	100
	indoors	inside box	0	0	8.6	0	0	2.1
		on floor	25.0	89.9	94.9	8.9	84.6	94.8
**Building #5 residence**	outdoors	inside box	**10.0†**	29.6	44.1	**84.0†**	94.6	94.6
		on pole	0	100	100	**100†**	100	100
	indoors	inside box	0	0	0	0	7.7	27.9
		on floor	20.0	95.0	79.7	71.9	94.3	100
**Building #6 outdoor market**	outdoors	inside box	**95.0†**	84.9	84.8	0	0	2.1
		on pole	68.4	100	100	100	100	100
	indoors	inside box	0	0	0	0	0	12.4
		on floor	0	69.8	69.5	**94.7†**	100	100
**Building #7 outdoor market**	outdoors	inside box	**0†**	100	100	0	12.8	17.6
		on pole	5.0	100	100	**100†**	100	100
	indoors	inside box	0	0	13.7	0	13.6	29.5
		on floor	0	29.6	44.1	**89.9†**	100	100
**Building #8 residence with courtyard**	outdoors	inside box	0	14.6	59.4	**0†**	0	0
		on pole	80.0	100	100	**79.7†**	100	100
	indoors	inside box	0	54.8	84.8	0	0	2.4
		on floor	**0†**	54.8	74.6	68.0	100	100
