# Supplementary material for: Aerial ULV control of Aedes aegypti with naled (Dibrom) inside simulated rural village and urban cryptic habitats
Source: PLoS One. 2018 Jan 19;13(1):e0191555. doi: 10.1371/journal.pone.0191555 (PMC5774805; doi:10.1371/journal.pone.0191555)
Supplement: S3 Table — All Transect sentinels were placed outdoors unprotected in the open at the top of 48-in high poles. (PDF) [file pone.0191555.s004.pdf]

S3 Table. **Abbott-corrected sentinel adult *Ae. aegypti* mosquito percent mortality at 1, 4, and 12 hr post-spray following the 29-30 October aerial naled applications over the Transect sites**. All Transect sentinels were placed outdoors unprotected in the open at the top of 48-in high poles.

Road Transect Location	Nearest Area	Description	Spinner Present?	29 October			30 October		
				1 hr	4 hr	12 hr	1 hr	4 hr	12 hr
**R 20**	MOUT South	1250 ft NE of MOUT	-	30.0	95.0	94.9	59.5	100	100
**R 19**	MOUT South	1000 ft NE of MOUT	-	63.2	89.4	100	94.9	100	100
**R 18**	MOUT South	750 ft NE of MOUT	**Yes**	90.0	100	100	89.9	100	100
**R 17**	MOUT South	500 ft NE of MOUT	**Yes**	0.0	100	100	100	100	100
**R 16**	MOUT South	250 ft NE of MOUT	**Yes**	70.0	100	100	100	100	100
**R 15**	MOUT South	250 ft SW of MOUT	**Yes**	100	100	100	100	100	100
**R 14**	MOUT South	500 ft SW of MOUT	-	26.3	100	100	100	100	100
**R 13**	MOUT South	750 ft SW of MOUT	-	35.0	100	100	100	100	100
**R 12**	MOUT South	1000 ft SW of MOUT	-	0.0	100	100	94.9	100	100
**R 11**	MOUT South	1250 ft SW of MOUT	-	0.0	100	100	100	100	100
**R 40**	Village	1250 ft N of Village	-	5.0	24.6	59.4	77.5	100	100
**R 39**	Village	1000 ft N of Village	-	10.0	89.9	100	62.7	100	100
**R 38**	Village	750 ft N of Village	-	25.0	84.9	94.9	84.8	100	100
**R 37**	Village	500 ft N of Village	-	45.0	100	100	69.6	100	100
**R 36**	Village	250 ft N of Village	-	100	100	100	84.8	100	100
**R 35**	Village	250 ft SW of Village	**Yes**	40.0	100	100	100	100	100
**R 34**	Village	500 ft SW of Village	**Yes**	100	100	100	100	100	100
**R 33**	Village	750 ft SW of Village	**Yes**	100	100	100	100	100	100
**R 32**	Village	1000 ft SW of Village	-	65.0	95.0	94.9	66.2	100	100
**R 31**	Village	1250 ft SW of Village	-	95.0	100	100	100	100	100
