# Supplementary material for: Aerial ULV control of Aedes aegypti with naled (Dibrom) inside simulated rural village and urban cryptic habitats
Source: PLoS One. 2018 Jan 19;13(1):e0191555. doi: 10.1371/journal.pone.0191555 (PMC5774805; doi:10.1371/journal.pone.0191555)
Supplement: S4 Table — The droplet spectrum for each location is partitioned into DV10, DV50 (VMD), and DV90. The symbol r denotes droplet density in droplets per mm2. (PDF) [file pone.0191555.s005.pdf]

S4 Table. Droplet analysis data from the 29-30 October aerial naled applications over the MOUT South, Village, and Control sites. The droplet spectrum for each location is partitioned into DV_{10}, DV_{50} (VMD), and DV_{90}. The symbol ρ denotes droplet density in droplets per mm^2.

			29 October				30 October			
**Site**	**Location**	**Type**	ρ	DV_{10}	DV_{50}	DV_{90}	ρ	DV_{10}	DV_{50}	DV_{90}
MOUT South	Wax Candle	outdoors	47.8	3.7	5.3	7.8	64.9	4.1	5.9	9.8
		indoors	23.3	3.9	5.5	8.1	52.6	4.3	6.2	10.3
	Bread Market	outdoors	48.9	3.8	5.7	9.5	53.4	4.7	6.8	11.8
		indoors	18.8	4.2	5.8	10.0	35.3	4.7	6.6	12.0
	Bazaar	outdoors	32.9	4.5	5.9	9.0	51.1	4.5	6.9	11.9
		indoors	17.7	4.1	5.7	8.0	19.7	4.2	6.1	9.1
	Meat Market	outdoors	24.5	4.4	6.1	9.6	70.9	4.5	6.5	11.6
		indoors	15.2	4.5	6.3	8.7	40.7	4.9	6.4	9.9
	Holiday Hotel	outdoors	30.9	4.0	5.9	9.2	18.8	5.0	7.1	11.6
		indoors (1st floor)	17.8	4.5	6.2	8.9	42.8	4.8	6.6	9.8
		indoors (2nd floor)	19.1	4.2	5.9	8.4	31.4	4.3	6.3	9.6
		outdoors (roof A)	33.6	4.0	5.9	9.0	81.9	4.2	6.4	11.7
		outdoors (roof B)	18.8	4.4	6.4	14.2	65.8	4.0	5.9	10.6
	American Hotel	outdoors	20.3	4.7	6.6	9.5	64.2	4.3	6.5	11.2
		indoors (1st floor)	11.3	4.2	5.7	8.0	23.7	3.7	5.1	7.8
		indoors (2nd floor)	11.2	4.6	6.2	9.0	15.5	3.8	5.5	7.9
	Fruit Market	outdoors	33.1	3.8	5.3	8.4	56.7	4.5	6.7	11.3
		indoors	12.5	3.5	5.9	8.6	19.3	4.0	5.8	8.9
	Jose Cantina	outdoors	22.7	3.8	5.6	10.0	37.4	4.6	6.6	11.4
		indoors	15.0	4.2	6.0	9.5	32.7	4.1	5.9	9.1
Village	Traffic Circle	outdoors	23.2	4.1	6.5	9.5	51.7	4.4	6.7	11.8
	Main Structure	indoors (1st floor)	10.9	4.6	6.5	9.6	46.0	3.9	5.8	9.9
		indoors (2nd floor)	11.2	4.4	5.9	9.3	26.7	4.6	6.8	10.3
		outdoors (roof)	24.8	4.0	5.5	7.9	44.0	4.3	6.4	10.9
	Building #2	outdoors	16.0	4.1	5.7	10.1	34.2	4.5	6.9	11.8
		indoors	8.3	4.0	5.6	9.6	24.0	4.1	6.1	9.9
	Building #3	outdoors	19.0	4.0	5.6	9.3	34.9	4.3	6.5	11.0
		indoors	12.0	4.4	5.9	8.3	21.6	4.4	6.7	10.8
	Building #4	outdoors	23.4	4.0	5.7	9.2	35.9	3.9	6.0	10.0
		indoors	10.6	4.0	5.8	9.2	33.3	4.2	6.4	11.5
	Building #5	outdoors	7.7	4.7	6.5	11.6	40.8	4.4	6.7	11.8
		indoors	11.7	3.9	5.6	8.4	23.9	4.1	6.2	9.6
	Building #6	outdoors	12.6	4.3	5.9	9.2	47.2	4.1	6.3	11.6
		indoors	3.4	4.6	6.5	9.7	31.4	4.3	6.6	11.6
	Building #7	outdoors	15.4	3.8	5.4	8.1	5.9	4.4	7.4	12.8
		indoors	15.3	3.8	5.3	7.8	13.1	5.1	8.4	15.4
	Building #8	outdoors	10.0	4.2	6.0	11.9	38.0	4.3	6.3	9.9
		indoors	12.1	4.1	5.8	8.2	22.6	4.3	6.7	11.5
Control	Spinner 1	outdoors	19.1	4.3	6.0	14.1	33.2	4.3	6.4	9.8
	Spinner 2	outdoors	19.3	4.2	5.7	7.5	24.5	4.2	6.3	10.5
