# Supplementary material for: Aerial ULV control of Aedes aegypti with naled (Dibrom) inside simulated rural village and urban cryptic habitats
Source: PLoS One. 2018 Jan 19;13(1):e0191555. doi: 10.1371/journal.pone.0191555 (PMC5774805; doi:10.1371/journal.pone.0191555)
Supplement: S5 Table — The droplet spectrum for each location is partitioned into DV10, DV50 (VMD), and DV90. The symbol r denotes droplet density in droplets per mm2. (PDF) [file pone.0191555.s006.pdf]

Supplementary information for Britch et al. Aerial ULV control of *Aedes aegypti* with naled (Dibrom) inside simulated rural village and urban cryptic habitats**.**

S5 Table. **Droplet analysis data from the 29-30 October aerial naled applications over the Transect and Control sites (all outdoors, unprotected).** The droplet spectrum for each location is partitioned into DV_{10}, DV_{50} (VMD), and DV_{90}. The symbol ρ denotes droplet density in droplets per mm^2.

			29 October				30 October			
**Site**	**Location**	**Description**	ρ	DV_{10}	DV_{50}	DV_{90}	ρ	DV_{10}	DV_{50}	DV_{90}
**Road transects adjacent to MOUT South**	**R18**	750 ft E of MOUT	20.4	3.9	5.8	8.8	29.3	4.5	6.6	11.5
	**R17**	500 ft E of MOUT	12.1	4.0	6.1	8.7	45.6	4.0	6.1	10.0
	**R16**	250 ft E of MOUT	16.9	3.8	5.2	7.1	25.8	4.4	6.6	11.0
	**R16A**	0 ft E of MOUT	21.5	4.0	5.4	8.8	34.0	3.8	6.2	11.3
	**R15A**	0 ft W of MOUT	10.4	4.4	6.4	10.6	28.9	4.0	6.3	11.6
	**R15**	250 ft W of MOUT	18.6	4.0	5.7	8.4	30.2	4.1	6.3	11.5
**Road transects adjacent to Village**	**R35A**	0 ft W of Village	21.0	4.0	5.8	8.7	36.0	4.0	6.4	11.2
	**R35**	250 ft W of Village	15.1	3.8	5.6	8.7	36.6	4.2	6.4	11.8
	**R34**	500 ft W of Village	16.4	4.3	6.1	9.9	35.6	4.5	6.9	12.9
	**R33**	750 ft W of Village	14.9	3.7	5.7	8.3	42.5	4.2	6.5	11.4
**Control**	**Spinner 1**	-	19.1	4.3	6.0	14.1	33.2	4.3	6.4	9.8
	**Spinner 2**	-	19.3	4.2	5.7	7.5	24.5	4.2	6.3	10.5
