# Supplementary material for: Aerial ULV control of Aedes aegypti with naled (Dibrom) inside simulated rural village and urban cryptic habitats
Source: PLoS One. 2018 Jan 19;13(1):e0191555. doi: 10.1371/journal.pone.0191555 (PMC5774805; doi:10.1371/journal.pone.0191555)
Supplement: S1 File — (PDF) [file pone.0191555.s007.pdf]

Britch et al. Aerial ULV control of *Aedes aegypti* with naled (Dibrom) inside simulated rural village and urban cryptic habitats.

Supplementary References

Referenced in the Introduction: "A survey of primary and gray literature shows that the great majority of both operational and experimental ground and aerial ULV and thermal fog applications against *Ae. aegypti* has been conducted with three organophosphate pesticides":

Malathion (37 publications):

Andis MD, Sackett SR, Carroll MK, Bordes ES. Strategies for the emergency control of arboviral epidemics in New Orleans. J Am Mosq Control Assoc. 1987; 3(2):125–10.

Andrighetti MTM, Macoris MLG, Takaku L, Galvani KC, Cardoso RP, Scandar SS, et al. Evaluation of the effect of insecticide malathion applied as ultra low volume with portable and vehicle-mounted machines on *Aedes aegypti* control. Revista de Patologia Tropical. 2013; 42(1):81–95.

Anon. Surveillance and control of *Aedes aegypti* in Bolivia. Epidemiol Bull. 1982; 3:7–7.

Bang YH, Gratz N, Pant CP. Suppression of a field population of *Aedes aegypti* by malathion thermal fogs and Abate larvicide. Bull World Health Organ. 1972; 46(4):554–558.

Brody MS. Report on *Aedes aegypti* surveillance and control in New Orleans, Louisiana. Newsletter on Dengue, Yellow Fever, and *Aedes aegypti* in the Americas. 1979; 8(1):8–11.

Brown JR, Melson RO, Tetreault GE. United States Navy pesticide aerial unit. Journal of the Florida Anti-Mosquito Assoc. 1989; 60:4–6.

Castle T, Amador M, Rawlins S, Figueroa JP, Reiter P. Absence of impact of aerial malathion treatment on *Aedes aegypti* during a dengue outbreak in Kingston, Jamaica. Rev Panam Salud Publica. 1999; 5(2):100–105.

Chadee DD. An evaluation of malathion ultralow volume spraying against caged and natural populations of *Aedes aegyp*ti in Trinidad, West Indies. Cahiers ORSTOM (Office de la Recherche Scientifique et Technique Outre-Mer) Serie Entomologie Medicale et Parasitologie. 1985; 23:71–4.

Conlon JM, Wooster MT, Calborn DM, Beck AF, Zimmerman J. Aerial spray operations during a dengue epidemic in Venezuela, 1990. Journal of the Florida Mosquito Control Association. 1990; 61(1):19–22.

Echevers G[†], Moura Lima M, Miranda Franco R, Calheiros LB. Results of spraying with ultra-low-volume malathion at ground level in Panama City. Bull Pan Am Health Organ. 1975; 9:232–237.

[†]These same data were presented also in Echevers G, Moura Lima M, Miranda Franco R, Calheiros LB. Ultralow volume malathion sprayings in Panama. Boletin De La Oficina Sanitaria Panamericana. 1975; 78:405–412.

Eliason DA, Kilpatrick JW, Babbitt MF. Evaluation of the effectiveness of the ultra low volume aerial application of insecticides against *Aedes aegypti* (L.) in Florida. Mosq News. 1970; 30(3):430–436.

Febles CR, Romney H. Follow-up on dengue - Puerto Rico. MMWR Surveill Summ. 1976; 25(9):65–66.

Focks DA, Kloter KO, Carmichael GT. The impact of sequential ultra-low volume ground aerosol applications of malathion on the population dynamics of *Aedes aegypti* (L.). Am J Trop Med Hyg. 1987; 36(3):639–647.

Fox I. Evaluation of ultra-low volume aerial and ground applications of malathion against natural populations of *Aedes aegypti* in Puerto Rico. Mosq News. 1980; 40(2):280–283.

Gratz NG. Emergency control of *Aedes aegypti* as a disease vector in urban areas. J Am Mosq Control Assoc. 1991; 7(3):353–365.

Hudson JE. The 1982 emergency ultralow volume spray campaign against *Aedes aegypti* adults in Paramaribo, Suriname. Bull Pan Am Health Organ. 1986; 20(3):294–303.

Kilpatrick JW, Eliason DA, Babbitt MF. Studies of the potential effectiveness of ultra low volume aerial applications of insecticides against *Aedes aegypti* (L.) larvae. Mosq News. 1970; 30(2):250–258.

Kilpatrick JW, Tonn RJ, Jatanasen S. Evaluation of ultra-low-volume insecticide dispensing systems for use in single-engined aircraft and their effectiveness against *Aedes aegypti* populations in South-East Asia. Bull World Health Organ. 1970; 42(1):1–14.

Lam WK, Tham AS. A field evaluation of the effectiveness of ULV application of malathion 96% technical grade and sumithion L-40S against *Aedes aegypti* (Linnaeus) and *Aedes albopictus* (Skuse) in Ipoh municipality, Perak, Malaysia. Trop Biomed. 1988; 5:81–88.

Lofgren CS, Ford HR, Tonn RJ, Jatanase.S. The effectiveness of ultra-low-volume applications of malathion at a rate of 6 US fluid ounces per acre in controlling *Aedes aegypti* in a large-scale test at Nakhon Sawan, Thailand. Vol. 42, Bull. World Health Organ. 1970; 42:15-25.

Lofgren CS, Ford HR, Tonn RJ, Bang YH, Siribodhi P. The effectiveness of ultra-low-volume applications of malathion at a rate of 3 US fluid ounces per acre in controlling *Aedes aegypti* in Thailand. Bull World Health Organ. 1970; 42(1):27–35.

Novak RJ, Gubler DJ. The effect of ground-applied ULV malathion on natural *Aedes aegypti* populations in Puerto Rico. Proceedings of the 72nd Annual Meeting of the New Jersey Mosquito Control Association. 1985; 212.

Obudho WO, Smith A, Awawo MA, Esozed S, Myamba J, Tarimo CS, et al. Annual Report of the Tropical Pesticides Research Institute. 1973; 93 pp.

Pant CP, Mount GA, Jatanasen S, Mathis HL. Ultra-low-volume ground aerosols of technical malathion for the control of *Aedes aegypti* L. Bull World Health Organ. 1971; 45(6):805–817.

Pant CP, Mathis HL. Residual effectiveness of ULV aerosol against *Aedes aegypti* in Bangkok; a study of sumithion and malathion applied by a portable ULV machine. Unpublished document, Geneva, World Health Organization. 1972; WHO/VBC/72.340.

Pant CP, Self LS, Gunawan S, Nelson MJ, Usman S, Wiseso G. Aerial spraying with malathion ULV using a single engine aircraft to control *Aedes aegypti* during an epidemic of dengue haemorrhagic fever at Semarang, Indonesia. Unpublished document, Geneva, World Health Organization. 1974; WHO/VBC/74.489.

Parker JD, Kahumbura JM, Tonn RJ, Shikonyi EN, Bang YH. Ultra-low volume applications of malathion for the control of *Aedes simpsoni* and *Aedes aegypti* in East Africa. East African Journal of Medical Research. 1977; 4(2-3):113–128.

Perich MJ, Davila G, Turner A, Garcia A, Nelson M. Behavior of resting *Aedes aegypti* (Culicidae: Diptera) and its relation to ultra-low volume adulticide efficacy in Panama City, Panama. J. Med. Entomol. 2000: 37:541–546.

Perich MJ, Tidwell MA, Williams DC, Sardelis MR, Pena CJ, Mandeville D, et al. Comparison of ground and aerial ultra-low volume applications of malathion against *Aedes aegypti* in Santo Domingo, Dominican Republic. J Am Mosq Control Assoc. 1990; 6(1):1–6.

Ribeiro H. The control of *Aedes aegypti* during the yellow fever epidemic in Luanda, Angola, in 1971. Bull World Health Organ. 1973; 48(4):504–505.

Self LS, Nelson MJ, Theos B, Wiseso G. A reduction in hospitalized cases of dengue haemorrhagic fever in Menado (Sulawesi), Indonesia after aerial spraying with ULV malathion to control *Aedes aegypti*. J Med Assoc Thai. 1977; 60(10):482–492.

Uribe LJ, Campos Garrido G, Nelson M, Tinker ME, Moquillaza J. Experimental application from aircraft of ULV malathion against *Aedes aegypti*, in a town in Colombia. Boletin de la Oficina Sanitaria Panamericana. 1983; 94:546–559.

Uribe LJ, Garrido GC, Nelson M, Tinker ME, Moquillaza J. Aerial ULV application trial of malathion against *Aedes aegypti* in a city of Colombia. Unpublished document, Geneva, World Health Organization. 1980; WHO/VBC/80.768.

Uribe LJ, Campos Garrido G, Nelson M, Tinker ME, Mowuillaza J. Experimental aerial spraying with ultra-low-volume (ULV) malathion to control *Aedes aegypti* in Buga, Colombia. Bull. Pan Am. Health Organ. 1984; 18:43–57.

Vythilingam I, Panart P. A field trial on the comparative effectiveness of malathion and Resigen by ULV application on *Aedes aegypti*. Southeast Asian J Trop Med Public Health. 1991; 22(1):102–107.

Yap HH, Khoo TC, Tan HT, Chung KK, Yahaya AM, Narayanan VS. Comparative adulticidal and larvicidal effects of thermal fogging formulations of Resigen and malathion against *Aedes aegypti* (Linnaeus) and *Culex quinquefasciatus* (Say) in urban areas, Malaysia. Trop Biomed. 1988; 5:125–130.

Yap HH, Khoo TC, Chee CS, Cheong WH. Comparative adulticidal and larvicidal effects of thermal fogging formulations of Dursban, malathion, and bioresmethrin against *Aedes aegypti* (Linnaeus) and *Culex quinquefascia*tus (Say) on Penang Island, Malaysia. Proceedings of the International Conference on Dengue/Dengue Haemorrhagic Fever, 1-3 September 1983, University of Malaya, Kuala Lumpur, Malaysia. 1983; 231–246.

Fenitrothion (11 publications):

Lam WK, Tham AS. A field evaluation of the effectiveness of ULV application of malathion 96% technical grade and sumithion L-40S against *Aedes aegypti* (Linnaeus) and *Aedes albopictus* (Skuse) in Ipoh municipality, Perak, Malaysia. Trop Biomed. 1988; 5:81–88.

Lee HL, Seleena P, Chiang YF. Preliminary field evaluation of the combined adulticidal and larvicidal activity of ULV-applied fenitrothion against mosquitoes. Trop Biomed. 1997; 14:147–149.

Loke SR, Sing KW, Teoh GN, Lee HL. Evaluation of Sumithion L-40 against *Aedes aegypti* (L.) and *Aedes albopictus* Skuse. Trop Biomed. 2015; 32(1):76–83.

Mathis HL, Self LS, Shim JC, Ree H-I. Ground aerosols of ultra-low-volume fenitrothion for the control of *Culex tritaeniorhynchus* in Pusan, Korea. Unpublished document, Geneva, World Health Organization. 1974; WHO/VBC/74.498.

Pant CP, Mathis HL, Nelson MJ, Phanthumachinda B. A large-scale field trial of ULV sumithion applied by a portable mist blower for the control of *Aedes aegypti*: a novel approach utilizing limited residual action and larviciding principle. Unpublished document, Geneva, World Health Organization. 1974; WHO/VBC/74.500.

Pant CP, Mathis HL, Nelson MJ, Phanthumachinda B. A large-scale field trial of ultra-low-volume fenitrothion applied by a portable mist blower for the control of *Aedes aegypti*. Bull World Health Organ. 1974; 51(4):409–415.

Pant CP, Mathis HL. Residual effectiveness of ULV aerosol against *Aedes aegypti* in Bangkok; a study of sumithion and malathion applied by a portable ULV machine. Unpublished document, Geneva, World Health Organization. 1972; WHO/VBC/72.340.

Pant CP, Nelson MJ, Mathis HL. Sequential application of ultra-low-volume ground aerosols of fenitrothion for sustained control of *Aedes aegypti*. Bull World Health Organ. 1973; 48(4):455–459.

Samutrapongse W, Pant CP. Sequential application of ULV Sumithion for sustained control of *Aedes aegypti* Linn. Use of Fontan, a back pack portable mist blower. Unpublished document, Geneva, World Health Organization. 1973;WHO/VBC/73.432.

Sánchez IAM, Tonn DR, Uribe LJ, Calheiros LB. A comparison of the effectiveness of different ways of applying insecticides to control or eradicate *Aedes aegypti* in Colombia. Bol of Sanit Panam. 1978; 84(1):24–37.

Vythilingham I. A field trial of the comparative effectiveness of fenitrothion and Resigen on *Aedes aegypti* by thermal fog application. Mosquito-Borne Diseases Bulletin. 1988; 4(4):99–102.

Naled (7 publications):

Altman RM, Berry RA, Joseph SR, Ludlam KW, Mallack J. Evaluation of seven insecticides applied as ground ULV aerosols for adult mosquito control. Proceedings of the 59th Annual Meeting of the New Jersey Mosquito Extermination Assoc. 1974; 59:177–179.

Brody MS. Report on *Aedes aegypti* surveillance and control in New Orleans, Louisiana. Newsletter on Dengue, Yellow Fever, and *Aedes aegypti* in the Americas. 1979; 8(1):8–11.

Brown JR, Melson RO, Tetreault GE. United States Navy pesticide aerial unit. Journal of the Florida Anti-Mosquito Assoc. 1989; 60:4–6.

Clark GG*, Reiter P, Gubler DJ. *Aedes aegypti* control trials using aerial ULV applications of naled in Puerto Rico. Arbovirus research in Australia, Proceedings from the 5th Symposium, August 28-September 1, 1989, Brisbane, Australia. 1989; 192–193.

**Data discussed in Clark et al. (1989) were originally presented in:* US Centers for Disease Control and Prevention. Efficacy of aerial application of Dibrom 14 against *Aedes aegypti* in San Juan, Puerto Rico. US CDC, San Juan, Puerto Rico; 1987. Dengue Surveillance Summary 47.

Jakob WL. Thermal fog tests against *Aedes aegypti* (L.) and *Anopheles albimanus* (Wied.). Mosq News. 1966; 26:118–121.

Lofgren C, Scanlon J, Israngura V. Evaluation of insecticides against *Aedes aegypti* (L.) and *Culex pipiens quinquefasciatus* Say (Diptera: Culicidae) in Bangkok, Thailand. Mosq News. 1967; 27:16–21.

Marcombe S, Carron A, Darriet F, Etienne M, Agnew P, Tolosa M, et al. Reduced efficacy of pyrethroid space sprays for dengue control in an area of Martinique with pyrethroid resistance. Am J Trop Med Hyg. 2009; 80(5):745–751.
