# Supplementary material for: Aerial ULV control of Aedes aegypti with naled (Dibrom) inside simulated rural village and urban cryptic habitats
Source: PLoS One. 2018 Jan 19;13(1):e0191555. doi: 10.1371/journal.pone.0191555 (PMC5774805; doi:10.1371/journal.pone.0191555)
Supplement: S2 File — Esri guidance on citing basemaps and maps created in ArcGIS. (PDF) [file pone.0191555.s008.pdf]

FAQ: What is the correct way to cite an ArcGIS Online basemap?

Question

What is the correct way to cite an ArcGIS Online basemap?

Answer

There are several ways to cite or attribute the ArcGIS Online basemaps that are used for publication, depending on the medium of publication. For each ArcGIS Online basemap, the proper attributions for the basemaps are as follows:

Map	Attribution
World Imagery	Sources: Esri, DigitalGlobe, GeoEye, i-cubed, USDA FSA, USGS, AEX, Getmapping, Aerogrid, IGN, IGP, swisstopo, and the GIS User Community
World Street Map	Sources: Esri, DeLorme, HERE, USGS, Intermap, iPC, NRCAN, Esri Japan, METI, Esri China (Hong Kong), Esri (Thailand), MapmyIndia, TomTom
World Topo Map	Sources: Esri, DeLorme, HERE, TomTom, Intermap, increment P Corp., GEBCO, USGS, FAO, NPS, NRCAN, GeoBase, IGN, Kadaster NL, Ordnance Survey, Esri Japan, METI, Esri China (Hong Kong), swisstopo, MapmyIndia, and the GIS User Community
National Geographic	Sources: National Geographic, Esri, DeLorme, HERE, UNEP-WCMC, USGS, NASA, ESA, METI, NRCAN, GEBCO, NOAA, iPC
Light Gray Canvas Map	Sources: Esri, DeLorme, HERE, MapmyIndia
USA Topo Maps	Copyright: © 2014 National Geographic Society, i-cubed
Ocean Basemap	Sources: Esri, GEBCO, NOAA, National Geographic, DeLorme, HERE, Geonames.org, and other contributors

To use Bing Maps as the basemap, users must adhere to Microsoft Print Rights. The logo must not be altered, and copyright notices must be included on the map.

The following are the correct ways to cite an ArcGIS Online basemap used for publication.

• ArcGIS Online basemaps used on ArcGIS Online content

When an ArcGIS Online basemap is used in ArcGIS Online content that is published, the acceptable method of attribution is by crediting the sources found in the Credits field of the item details for each ArcGIS Online map, task, or application being used. Credits should also be displayed in the lower right corner of the map or image.

Figure 1: Example of the Credit field

Access and Use Constraints

esri This work is licensed under the Web Services and API Terms of Use.
View Summary | View Terms of Use

Map Contents

Topographic:
http://services.arcgisonline.com/ArcGIS/rest/services/World_Topo_Map/MapServer

Properties

Tags	world, topo maps, topographic, topography, maps, topo, community, shaded relief, land cover, administrative boundaries, cities, water, parks, landmarks, transportation, roads, highways, airports, railroads, buildings, community map, content sharing program, basemap, current, elevation
Credits	Sources: Esri, DeLorme, NAVTEQ, TomTom, Intermap, increment P Corp, GEBCO, USGS, FAO, NPS, NRCAN, GeoBase, Kadaster NL, Ordnance Survey, Esri Japan, METI, and the GIS User Community
Size	1 KB
Extent	Left: -180 Right: 180<br>Top: 90 Bottom: -90

• ArcGIS Online basemaps used in printed material

When using an ArcGIS Online basemap in printed materials, reference the use of the basemap with a statement such as:

'Maps throughout this book were created using ArcGIS® software by Esri. ArcGIS® and ArcMap™ are the intellectual property of Esri and are used herein under license. Copyright © Esri. All rights reserved. For more information about Esri® software, please visit www.esri.com.'

• ArcGIS Online basemaps used at public exhibitions

When an ArcGIS Online basemap is used in a public exhibition, the attribution is included in the lower right corner of the map or image.

In some instances, users place all attributions in an exhibit on a single 'credit panel' at the end of each gallery or exhibition, instead of placing attributions on each map used. As long as this credit panel is located near the maps being used, there is no need to individually attribute each map. Nevertheless, to properly cite a publication, the copyright attribution must be on or near the map or image being used.

Related Information

- ArcGIS Online Content Help: Displaying copyrights on ArcGIS Online content
- ArcGIS Online Content Help: Using static maps in reports
- ArcGIS Online Basemaps Information Guide
- ArcGIS Blog: Using and citing Esri data

Last Modified: 5/8/2016

Article ID: 000012040

Software: ArcGIS Online Current
